# A Bayesian Inference Model for Metamemory

**DOI:** 10.1037/rev0000270

**Published:** 2021-05-27

**Authors:** Xiao Hu, Jun Zheng, Ningxin Su, Tian Fan, Chunliang Yang, Yue Yin, Stephen M. Fleming, Liang Luo

**Affiliations:** 1 Institute of Developmental Psychology, Beijing Normal University; 2 Collaborative Innovation Center of Assessment Toward Basic Education Quality, Beijing Normal University; 3 Department of Experimental Psychology, University College London; 4 Wellcome Centre for Human Neuroimaging, University College London; 5 Max Planck UCL Centre for Computational Psychiatry and Ageing Research, University College London

**Keywords:** metamemory, metacognition, processing experience, prior belief, Bayesian

## Abstract

The dual-basis theory of metamemory suggests that people evaluate their memory performance based on both processing experience during the memory process and their prior beliefs about overall memory ability. However, few studies have proposed a formal computational model to quantitatively characterize how processing experience and prior beliefs are integrated during metamemory monitoring. Here, we introduce a Bayesian inference model for metamemory (BIM) which provides a theoretical and computational framework for the metamemory monitoring process. BIM assumes that when people evaluate their memory performance, they integrate processing experience and prior beliefs via Bayesian inference. We show that BIM can be fitted to recall or recognition tasks with confidence ratings on either a continuous or discrete scale. Results from data simulation indicate that BIM can successfully recover a majority of generative parameter values, and demonstrate a systematic relationship between parameters in BIM and previous computational models of metacognition such as the stochastic detection and retrieval model (SDRM) and the meta-*d*′ model. We also show examples of fitting BIM to empirical data sets from several experiments, which suggest that the predictions of BIM are consistent with previous studies on metamemory. In addition, when compared with SDRM, BIM could more parsimoniously account for the data of judgments of learning (JOLs) and memory performance from recall tasks. Finally, we discuss an extension of BIM which accounts for belief updating, and conclude with a discussion of how BIM may benefit metamemory research.

Metamemory refers to the processes for monitoring and controlling memory activities (Nelson & Narens, 1990). Many studies have shown that metamemory monitoring significantly influences subsequent learning processes, and understanding the mechanisms underlying metamemory monitoring is important for improving learning performance (for a review, see Bjork et al., 2013). Previous studies have shown that people cannot directly monitor their memory strength when they give metamemory judgments (or predictions) about their memory performance. Instead, they infer their memory performance based on a variety of cues, such as item difficulty, study duration, and perceptual features of study materials (Frank & Kuhlmann, 2017; Koriat, 1997; Rhodes & Castel, 2008).

One of the most influential theories in the metamemory literature is the dual-basis theory, which suggests that a cue can affect metamemory judgments through processing experience, prior beliefs, or both (Koriat et al., 2004). Experience-based metamemory judgments are assumed to rely on the experience derived from the processing of study materials. One important type of processing experience is processing fluency, which refers to the subjective experience of ease or difficulty with which we are able to process the items (Oppenheimer, 2008). For example, people ascribe higher confidence in their memory performance to word pairs when the semantic relatedness between cue and target words is higher because the subjective experience of processing high-relatedness word pairs is more fluent than that of low-relatedness word pairs (Undorf & Erdfelder, 2015). In contrast, belief-based metamemory judgments are assumed to rely on people’s prior beliefs about how a cue can affect memory performance. For example, people give higher confidence to high-relatedness than low-relatedness word pairs because they believe that high-relatedness word pairs are easier to remember (Mueller et al., 2013). Although most researchers agree that both processing experience and prior beliefs affect metamemory monitoring, there is a still debate about the degree to which each contributes to metamemory judgments (Frank & Kuhlmann, 2017; Hu et al., 2015; Mueller & Dunlosky, 2017; Mueller, Dunlosky, Tauber, et al., 2014; Mueller et al., 2013, 2016; Su et al., 2018; Undorf & Erdfelder, 2015; Undorf et al., 2017, Yang, Huang, et al., 2018).

Although the dual-basis theory suggests that metamemory monitoring depends on both current processing experience and people’s prior beliefs about their overall memory ability, it does not indicate how people combine processing experience and prior beliefs to evaluate their memory performance. Recent studies on metamemory monitoring mainly focus on whether processing experience or prior beliefs can mediate the cue effect on metamemory judgments (Frank & Kuhlmann, 2017; Su et al., 2018; Undorf & Erdfelder, 2015; Undorf et al., 2017; Yang, Huang, et al., 2018). However, few have characterized the cognitive process about how processing experience and prior beliefs are integrated during metamemory monitoring. In addition, there is a lack of formal computational models to quantitatively explain the role of processing experience and prior beliefs in metamemory judgments. Compared with descriptive theories, computational models can more precisely characterize the cognitive processes underlying behavior, allowing more detailed empirical predictions (Lewandowsky & Farrell, 2011). While previous descriptive theories only suggest that both processing experience and prior beliefs contribute to metamemory monitoring, formal computational models may quantitatively predict to what extent experience and beliefs affect metamemory judgments, and whether these effects vary across experimental conditions.

To date, relatively few studies have developed formal computational models to explain the metamemory monitoring process (Jang et al., 2012; Sikström & Jönsson, 2005). For example, Jang et al. (2012) propose the stochastic detection and retrieval model (SDRM) to describe how people give metamemory judgments based on processing experience. The SDRM assumes that people give their metamemory judgments based on their experience during the memory process, which is correlated with (but not the same as) the objective memory strength. People compare their processing experience with a set of confidence criteria, according to which they evaluate their performance in the memory test. SDRM is an important computational model that attempts to explain the metamemory process. It emphasizes the role of processing experience in metamemory monitoring and suggests potential dissociation between the objective memory strength determining memory performance and the subjective processing experience utilized in the metamemory process, which is consistent with the recent second-order model for metacognition (Fleming & Daw, 2017). Although SDRM does not explicitly explain how prior beliefs about memory ability feed into this metamemory monitoring process, it is possible that people may set confidence criteria based on their prior beliefs. For example, they may set liberal confidence criteria and frequently give high-confidence ratings when they believe they have high memory ability, or use conservative criteria if they believe their memory performance should be low. However, SDRM does not formally and quantitatively characterize how people integrate current processing experience and their prior beliefs about memory ability when they evaluate their memory performance.

In this article, we present a Bayesian inference model for metamemory (BIM) to quantitatively explain how people make metamemory judgments based on both processing experience and prior beliefs. Similar to SDRM, BIM assumes that people are not able to monitor their objective memory strength, and instead only gain access to their processing experience during learning, which in turn is correlated with memory strength. However, in contrast to SDRM, BIM assumes that people need to integrate their processing experience and prior beliefs through a Bayesian inference to evaluate their memory performance. Previous computational models on perceptual confidence judgments suggest that people’s confidence judgments are grounded in a Bayesian inference that integrates observed evidence and prior beliefs (Fleming & Daw, 2017; Pouget et al., 2016). Similarly, BIM assumes that during metamemory monitoring, people infer the posterior distribution of their memory strength through a Bayesian inference process in which they integrate their current processing experience and prior beliefs about their overall memory ability. BIM makes predictions about the distribution of metamemory judgments, allowing direct empirical evaluation. BIM can also estimate to what extent prior beliefs and processing experience each contribute to metamemory judgments. BIM is consistent with the dual-basis theory and emphasizes an important role for both experience and beliefs in metamemory monitoring.

The remainder of this article is organized as follows. First, we describe the basic ideas and mathematical structures of BIM. We discuss the relationship between BIM and SDRM, and introduce in detail how people integrate processing experience and prior beliefs to evaluate their memory performance under the assumptions of BIM. We will show that BIM can be fitted to data from both recall and recognition tests with confidence ratings on either a continuous or discrete scale. Next, we simulate data from BIM with different parameter values to investigate how the change in parameter values could predict the change in distributions of confidence and performance. We also examine whether we can successfully recover the parameters in BIM via fits to the simulated data, and whether there are significant relationships between parameters in BIM and other computational models of confidence such as SDRM and the meta-*d*′ model (Jang et al., 2012; Maniscalco & Lau, 2012). We then fit BIM to confidence rating and memory performance data obtained from four studies. In Studies 1–3, we fit BIM to data sets from several experiments on judgments of learning (JOLs), a canonical form of prospective metamemory monitoring (Nelson & Narens, 1990). We compare the results from BIM in Studies 1–2 and predictions based on previous studies on JOLs to examine if BIM is consistent with previous theories of metamemory. In Study 3, we conduct a stronger test of the assumptions about the Bayesian inference process in BIM. We also compare the fit of BIM and SDRM to ask which model could better account for the data. In Study 4, we demonstrate an example of how to apply BIM to a recognition memory test with retrospective confidence ratings. Finally, we use simulated data sets to explore a potential extension of BIM which accounts for belief updating.

## Details of BIM

In this section, we describe the ideas and mathematical equations behind BIM. BIM is closely related to SDRM, and here we first discuss the qualitative similarities and differences between BIM and SDRM. Then, we introduce the mathematical details of BIM to illustrate how people integrate processing experience and prior beliefs through a Bayesian inference to evaluate their memory performance.

### Relationship Between BIM and SDRM

BIM is similar to SDRM in assuming that the processing experience utilized in the metamemory monitoring process and the objective memory strength that determines memory performance arise from distinct distributions (Jang et al., 2012). Both models assume that there is a correlation between the distributions of objective memory strength and subjective processing experience,^1^ which can be represented by a free parameter ρ in the model. In addition, both models make assumptions that memory performance depends on whether the objective memory strength is higher than a criterion for recall, and that confidence ratings rely on the current processing experience for each trial sampled from a processing experience distribution.

However, BIM also differs from SDRM in four important ways. First, SDRM assumes that people give their confidence ratings based on a comparison between the current processing experience with a set of confidence criteria, which are free parameters in SDRM. However, SDRM does not formally and quantitatively explain how people set the confidence criteria in the metamemory process, or why the mean and variance of the confidence criteria may differ across experimental conditions. In contrast, BIM offers a formal explanation of how people use the confidence scale based on a Bayesian inference aimed at evaluating their memory performance. BIM assumes that during the metamemory process, people integrate the current processing experience and their prior beliefs about overall memory ability through a Bayesian inference to infer the posterior probability that the current item can be correctly answered during the memory test, and this posterior probability represents people’s confidence about their performance. If people’s confidence about performance mainly relies on prior beliefs, then the confidence ratings reported in memory tasks should be closely distributed around the prior beliefs about memory ability, and the variance of reported confidence ratings should be low. However, if processing experience plays an important role in the metamemory process, then the variance of the reported confidence ratings should be high because confidence ratings closely track the variation of processing experience across trials.

Second, in SDRM, confidence can be rated on an *n*-point rating scale when there are (*n* − 1) confidence criteria. Thus, SDRM can only be applied to confidence data on a discrete rating scale. In contrast, BIM is originally designed to account for confidence ratings on a continuous scale. BIM assumes that people’s confidence about memory performance is represented by the estimated probability that each item can be correctly answered during the memory test, which is based on the posterior distribution of memory strength inferred via Bayesian inference. This posterior probability should be between 0 and 1, and thus the confidence ratings predicted by BIM are on a 0–1 *continuous scale*. Furthermore, BIM assumes that when people report their confidence in a task using a continuous scale, the reported confidence is simply the sum of the posterior probability obtained via Bayesian inference and random noise, and there is a tight linear relationship between reported confidence ratings and the posterior probability predicted by BIM. However, BIM can also be applied to confidence ratings on a discrete scale. BIM assumes that when people report their confidence on an *n*-point scale, they divide the 0–1 *continuous scale* for the posterior probability into *n* intervals with equal lengths, and each interval can be seen as a point on the *n*-point scale. Thus, BIM can be fitted to confidence data on either a continuous or discrete scale.

Third, BIM assumes that in a certain experimental condition, the recall criterion is constant for a participant. In addition, when people rate confidence on a discrete scale, they divide the 0–1 *continuous scale* into equal-length bins, and thus the criteria on the confidence scale are also constant in BIM. In contrast, SDRM assumes that the recall criterion (*C*_*M*_) and confidence criteria (the *C*_*i*_s) may be variable across trials, and use two standard deviation parameters (σ_*M*_ and σ_*C*_) to characterize the variation of these criteria (Jang et al., 2012). However, when SDRM is fitted to data in a single experimental condition, σ_*M*_ and σ_*C*_ are typically nonidentifiable. These two parameters are meaningful when we fit SDRM to data from two or more different experimental conditions, in which we may constrain one or both of these parameters to be the same across conditions, and see whether allowing these standard deviation parameters to vary across conditions can change the model predictions.^2^ BIM is more similar to SDRM in which σ_*M*_ and σ_*C*_ are set to 0 and thus the variation of recall and confidence criteria is prohibited.

Finally, SDRM is suitable for data from recall tasks but not recognition tasks. SDRM assumes that the objective memory strength for each trial comes from a single distribution, and people correctly answer a trial when their memory strength is higher than a recall criterion. These assumptions in SDRM are suitable for the retrieval process in recall tasks. However, in a recognition test such as a Yes/No task or 2-alternative forced-choice (2AFC) task, we often assume that there are two types of stimuli with different distributions of memory strength, which determine people’s Type I response based on signal detection theory (SDT). These assumptions about recognition memory are outside the scope of SDRM. However, BIM can be extended to account for data from recognition tasks, in which we can combine SDT and BIM to explain the retrospective confidence rating process after a Type I response. Specifically, BIM assumes that people integrate their prior beliefs and processing experience related to either type of stimulus and then rate their confidence via Bayesian inference.

In the following sections, we first introduce the mathematical details of BIM for recall tasks with (prospective or retrospective) confidence ratings on a continuous or discrete scale. Then, we illustrate how to extend BIM to account for retrospective confidence ratings in recognition tasks.

### BIM for Recall Tasks With Continuous Confidence

The basic form of BIM is applicable to recall tasks with confidence ratings on a continuous scale from 0 to 1. In these tasks, people need to learn a series of stimuli (e.g., words) during a learning phase and are then required to recall an answer for each trial during the memory test. Before or after the test for each item, people are asked to give a (prospective or retrospective) confidence rating about memory performance, in which they need to estimate the probability of correctly recalling the item in the memory test based on an inference about their memory strength for this item (Dunlosky & Metcalfe, 2009). BIM makes a simple assumption that the distribution of objective memory strength *m* is a normal distribution for each individual with an unknown mean μ_*m*_ (which may vary between individuals) and a standard deviation of 1. The recall performance for an item depends on the sampled memory strength relative to the criterion for recall, which is set to the arbitrary constant 0. An item can be correctly recalled only when its memory strength is higher than 0. These assumptions are similar to those in SDRM except that the mean of the memory strength distribution is fixed in SDRM while the recall criterion is variable (Jang et al., 2012).

Consistent with the dual-basis theory for metamemory (Koriat, 1997; Koriat et al., 2004), BIM assumes that people do not have direct access to the objective memory strength for an item when they give a confidence rating. Instead, they infer their memory performance according to their subjective processing experience during the memory process. In other words, BIM assumes that while actual memory performance solely depends on the objective memory strength, people’s confidence ratings rely on their subjective processing experience but not the objective memory strength. This assumption is consistent with similar assumptions inherent to both SDRM (Jang et al., 2012) and the second-order model in the Bayesian framework for perceptual metacognition proposed by Fleming and Daw (2017).

**Figure 1 fig1:**
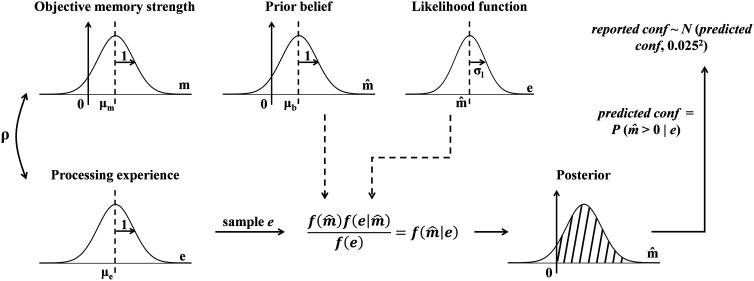
Illustration of BIM for Recall Tasks With Continuous Confidence Ratings

In BIM, the subjective processing experience for each item is assumed to arise from a distribution of processing experience for each individual with a mean of μ_*e*_ (which may vary between individuals) and a standard deviation of 1. We note that BIM makes no assumptions regarding whether people infer their memory performance based on the processing experience before or after the recall test, and thus BIM should be applicable to either prospective or retrospective confidence ratings in recall tasks. In addition, as in SDRM and the second-order model (Fleming & Daw, 2017; Jang et al., 2012), the distributions of objective memory strength and processing experience in BIM are assumed to be correlated and drawn from a bivariate normal distribution with a correlation coefficient ρ (see Figure 1).[Fig fig1]

According to previous studies on metamemory, the mean of the objective memory strength distribution (μ_*m*_) may differ from that of the processing experience distribution (μ_*e*_). For example, when rating prospective confidence [i.e., the judgment of learning (JOL)] about memory performance in a future recall test for words presented in large or small font size during learning, people often predict that large words will be easier to recall at test than small words (Hu et al., 2015; Mueller, Dunlosky, Tauber, et al., 2014; Su et al., 2018; Yang, Huang, et al., 2018). This is partly due to a more fluent processing experience for large than small fonts during the perceptual identification of each word in the learning process, suggesting that the strength of processing experience should be higher for large than small words (Yang, Huang, et al., 2018). However, objective memory performance typically does not differ between large and small words, suggesting that objective memory strength may remain similar across words with different font size (Hu et al., 2015; Mueller, Dunlosky, Tauber, et al., 2014; Su et al., 2018; Yang, Huang, et al., 2018). These results indicate that the distributions of objective memory strength and processing experience in BIM may have different means.

For each item, participants take a sample from the processing experience distribution as the subjective experience for the current item. The sampling of processing experience is conditional on the objective memory strength for the item because the objective memory strength and processing experience are correlated with a correlation coefficient ρ. Let us suppose that the processing experience for an item is *e*. Participants then need to infer their memory strength $\hat{m}$ based on this processing experience. We should note that here the notation $\hat{m}$ refers to participants’ subjective estimates of memory strength, which may differ from their objective memory strength *m*. BIM assumes that participants apply Bayesian inference to infer the memory strength (see Figure 1):

$$f(\hat{m}|e)=\frac{f\left(\hat{m}\right)f\left(e|\hat{m}\right)}{f\left(e\right)}$$ 1

In Equation 1, $f(\hat{m}|e)$ is the probability density function (PDF) of the posterior distribution of the memory strength, and reflects participants’ inference about the memory strength for the current item given the processing experience *e*. $f(\hat{m}$) is the PDF of the prior distribution of the memory strength and reflects participants’ prior belief about their overall memory performance in the test. BIM assumes that this prior belief about memory strength can be characterized by a normal distribution for each individual with a mean of μ_*b*_ (which may vary between individuals) and a standard deviation of 1 (see Figure 1). The area under the prior belief distribution above the criterion for recall, 0, reflects participants’ prior belief about the proportion of items they can recall in the memory test. Thus, participants believe they have better memory ability when μ_*b*_ is higher. For example, if participants are asked to predict their future memory performance in an immediate recall test and a delayed test (e.g., after 1 week), μ_*b*_ should typically be higher when participants rate their confidence for an immediate than a delayed test because they usually believe that their overall memory strength will be higher for the immediate test.

$f(e|\hat{m})$ in Equation 1 is the likelihood function, and reflects the probability that participants obtain a processing experience *e* given the estimated memory strength $\hat{m}$. This likelihood function encodes participants’ knowledge about the relationship between $\hat{m}$ and *e*. BIM assumes that the likelihood function is another normal distribution with a mean of $\hat{m}$ and a standard deviation of σ_*l*_, which is a free parameter and may vary between individuals (see Figure 1). *e* is more likely to be close to $\hat{m}$ when σ_*l*_ is small, suggesting that participants suppose memory strength can accurately predict the processing experience. In contrast, the processing experience is uncertain for a given memory strength $\hat{m}$ when σ_*l*_ is large, indicating that participants do not assume there is a close relationship between memory strength and processing experience. Finally, *f(e)* is the normalizing constant in Bayes’ theorem.

Based on the assumptions above, we can obtain the posterior distribution for the inferred memory strength $\hat{m}$ given the current processing experience *e* (i.e., $f(\hat{m}|e$), which is a normal distribution with mean and variance as follows (see Section S1 in Supplemental Materials, for the calculation):

$$E(\hat{m}|e)=\frac{e+\sigma_{l}^{2}\mu_{b}}{1+\sigma_{l}^{2}}$$ 2

$$\mathrm{Var}(\hat{m}|e)=\frac{\sigma_{l}^{2}}{1+\sigma_{l}^{2}}$$ 3

The mean of the posterior distribution for memory strength, $E(\hat{m}|e$), represents the overall memory strength for the current item inferred by participants. From Equation 2, we can see that the posterior mean is a weighted average of the current processing experience and the mean of the prior distribution (i.e., prior belief; Ma, 2019). Thus, we can obtain the relative weights (or proportions) for the contribution of processing experience (*P*_exp_) and prior beliefs (*P*_belief_) to participants’ inference on memory strength:

$$P_{\exp}=\frac{1}{1+\sigma_{l}^{2}}$$ 4

$$P_{\mathrm{belief}}=\frac{\sigma_{l}^{2}}{1+\sigma_{l}^{2}}$$ 5

These proportions can be estimated from empirical data, and the sum of the two proportions is 1.

From Equations 4 and 5, we should note that processing experience contributes more (compared with prior beliefs) to participants’ inference on memory strength when σ_*l*_ (the standard deviation of the likelihood function) is lower (and vice versa). This is easy to understand according to the model assumptions: Participants do not assume there is a close relationship between objective memory strength and processing experience when σ_*l*_ is very high. Thus, they will not rely on processing experience when they infer their memory performance. In order to predict the objective memory strength, they can only rely on their prior beliefs about their overall memory ability. In contrast, when σ_*l*_ is low, participants assume that objective memory strength can accurately predict processing experience, and thus should rely more on processing experience when inferring their memory performance. The contribution of processing experience and prior beliefs to participants’ inference on memory is the same when σ_*l*_ = 1.

After participants infer the posterior distribution for memory strength $\hat{m}$ based on the current processing experience *e*, they can give a (prospective or retrospective) confidence rating for the current item which reflects the probability of correctly recalling the item at test. Because the recall criterion is fixed to 0 in BIM, this confidence rating is just the probability that $\hat{m}$ is higher than the recall criterion 0 obtained from the posterior distribution^3^:

$$\text{predicted conf}=P(\hat{m}>0|e)=\Phi (\frac{E\left(\hat{m}|e\right)}{\sqrt{\mathrm{Var}\left(\hat{m}|e\right)}})=\Phi (\frac{e+\sigma_{l}^{2}\mu_{b}}{\sigma_{l}\sqrt{1+\sigma_{l}^{2}}})$$ 6

in which Φ is the cumulative density function for the standard normal distribution.

The posterior probability that $\hat{m}$ is higher than 0 represents the confidence rating predicted by BIM. We then need a function to link the predicted confidence from BIM and the reported confidence ratings from empirical data, through which we could build the likelihood function for confidence ratings and estimate the parameters in BIM (for further discussion about the intermediate function that links model predictions and empirical data, see Chapter 4.3.2 in Lewandowsky & Farrell, 2011). One simple way of linking the predicted and reported confidence is to assume that the reported confidence is the sum of the predicted confidence and a random noise term drawn from a normal distribution with a mean of 0 and a standard deviation of σ_noise_ (note that σ_noise_ cannot be reduced to 0 as this leads the likelihood of reported confidence to be 0 or infinite). Previous computational model fits to confidence data have revealed that nonspecific noise may be included into confidence ratings when confidence is reported on a continuous scale (Fleming et al., 2018; Hu et al., 2019). In line with a previous computational model of perceptual confidence (Fleming et al., 2018), BIM sets the value of σ_noise_ to .025, such that any given rating is made with a precision of approximately ±5%^4^:

$$\text{reported conf}˜N(\text{predicted conf},0.025^{2})$$ 7

In a typical metamemory task, participants need to learn many items and report a confidence rating for each item, allowing the construction of a distribution of confidence ratings. We are able to fit BIM to the empirical confidence distribution to estimate the model parameters. The free parameters in BIM are μ_*m*_ (mean of the distribution of objective memory strength), ρ (correlation between the distribution of objective memory strength and processing experience), μ_*e*_ (mean of the distribution of processing experience), μ_*b*_ (mean of the prior belief distribution), and *P*_exp_ (proportion for the contribution of processing experience to the inference on memory strength). BIM predicts that the mean of the confidence distribution is related to the model parameters as follows (see Section S1 in Supplemental Materials, for the calculation):

$$M_{\mathrm{conf}}=\Phi (\frac{\mu_{e}P_{\exp}+\mu_{b}P_{\mathrm{belief}}}{\sqrt{1-P_{\exp}P_{\mathrm{belief}}}})$$ 8

in which *P*_belief_ is equal to 1 minus *P*_exp_.

We should note that the mean confidence is related to a linear combination of the mean for the distributions of processing experience (μ_*e*_) and prior belief (μ_*b*_). The effect of μ_*e*_ on mean confidence is larger (compared with μ_*b*_) when *P*_exp_ is higher (i.e., when participants rely more on processing experience when they infer their memory strength), and vice versa. Thus, the contribution of processing experience and prior beliefs to confidence ratings depends on how much participants utilize experience and beliefs during their inference on memory strength.

During model development, we found that we could estimate most of the free parameters from empirical data except for μ_*e*_ and μ_*b*_ because these two parameters are nonidentifiable. Mean confidence is related to a linear combination of μ_*e*_ and μ_*b*_, and the effect of a change in μ_*e*_ on mean confidence can be mimicked by a change in μ_*b*_. However, we can use the mean confidence *M*_conf_ as a free parameter (instead of two parameters μ_*e*_ and μ_*b*_), and *M*_conf_ can be estimated directly from confidence data. Thus, when we fit BIM to empirical data sets, the free parameters are *P*_exp_, *M*_conf_, μ_*m*_, and ρ.

BIM can be fitted to empirical data using maximum likelihood estimation. In a recall task, when we obtain the data of recall performance (0 or 1, *representing an incorrect* or *correct answer*) and confidence rating (on 0–1 *continuous scale*) for each trial, we can compute the likelihood for a trial with a certain confidence rating and recall performance, and then find the value of four free parameters in BIM that maximizes the sum of log-likelihood for all trials (see Section S2 in Supplemental Materials, for the computation of likelihood function for BIM with continuous confidence ratings).

In BIM, we set the standard deviations to 1 for three separate distributions, including the distributions of objective memory strength, processing experience, and prior beliefs. These standard deviation parameters are constrained to make the model identifiable. We note that when we allow the standard deviations to vary, the effect of a change in these standard deviation parameters on model predictions can be mimicked by the effect of a change in other free parameters in BIM. For example, the standard deviation of objective memory strength distribution is simply a scaling parameter for μ_*m*_, and a change in the standard deviation of processing experience and prior beliefs can be traded off by a change in *P*_exp_ (or σ_*l*_), μ_*e*_, and μ_*b*_ (although μ_*e*_ and μ_*b*_ are not identifiable). In Section S3 of Supplemental Materials, we discuss the trade-off between these parameters in more detail.

### BIM for Recall Tasks With Discrete Confidence

**Figure 2 fig2:**
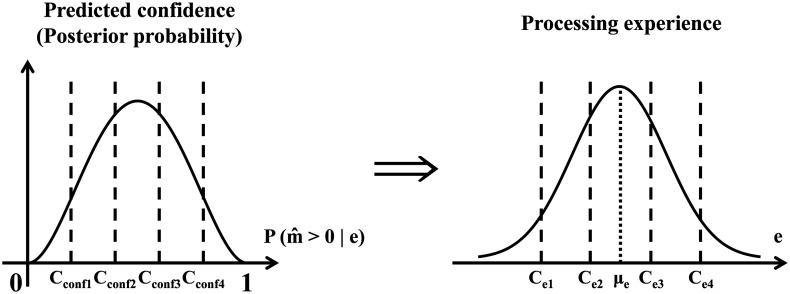
Illustration of Transformation From the Criteria on the Distribution of Predicted Confidence (Or the Distribution of the Posterior Probability That Estimated Memory Strength is Higher Than 0) Into Confidence Criteria on the Distribution of Processing Experience. A 5-Point Scale is Used as an Example

In many studies, participants are asked to rate their confidence on an *n*-point discrete scale rather than a continuous scale (Rahnev et al., 2020). For mathematical simplicity, BIM assumes that when people report their confidence on an *n*-point scale, they divide the posterior probability that $\hat{m}$ is higher than 0 [i.e., *P* ($\hat{m}$ > 0 | *e*), which is on a 0–1 *continuous scale*] into *n* intervals with equal lengths, and each interval can be seen as a point on the *n*-point scale. For example, when confidence is rated on a 5-point scale, people divide the 0–1 *continuous scale* for the posterior probability into five equal-length bins: 0–.2, .2–.4, .4–.6, and so on. Thus, BIM assumes that there are (*n* − 1) confidence criteria (denoted by *C*_conf(1)_, *C*_conf(2)_, … *C*_conf(*n* − 1)_) on the 0–1 *continuous scale* of posterior probability and these confidence criteria are fixed (see Figure 2),^5^ which can be computed as:

$$C_{\mathrm{conf}\left(i\right)}=\frac{i}{n}\;1\leq i\leq n-1$$ 9

[Fig fig2]

We already know that the posterior probability $P(\hat{m}>0|e)$ is computed based on the processing experience *e* (see Equation 6). Thus, the (*n* − 1) confidence criteria on the 0–1 *continuous scale* for $P(\hat{m}>0|e)$ can be transformed into (*n* − 1) confidence criteria on the distribution of processing experience (denoted by *C*_*e*(1)_, *C*_*e*(2)_, … *C*_*e*(n − 1)_) (see Figure 2):

$$C_{\mathrm{conf}\left(i\right)}=\Phi (\frac{C_{e\left(i\right)}+\sigma_{l}^{2}\mu_{b}}{\sigma_{l}\sqrt{1+\sigma_{l}^{2}}})\;1\leq i\leq n-1$$ 10

$$C_{e(i)}=\sigma_{l}\sqrt{1+\sigma_{l}^{2}}\Phi^{-1}(C_{\mathrm{conf}\left(i\right)})-\sigma_{l}^{2}\mu_{b}\;1\leq i\leq n-1$$ 11

in which Φ^−1^ represents the inverse of cumulative density function for the standard normal distribution. Thus, people give a rating of 1 on the *n*-point confidence scale when processing experience is lower than *C*_*e(1)*_, and 2 when processing experience is between *C*_*e(1)*_ and *C*_*e(2)*_, and so on.

From Equation 11, we can see that the parameter μ_*b*_ affects the mean of all confidence criteria on the processing experience distribution. For example, confidence criteria are more liberal (i.e., with a lower mean) when μ_*b*_ is higher, suggesting that people believe they have better memory ability. The parameter σ_*l*_ (or *P*_exp_, see Equation 4) can also influence the mean of confidence criteria, and a higher value of σ_*l*_ (i.e., lower *P*_exp_, suggesting that people rely more on prior beliefs to rate their confidence) makes the confidence criteria more liberal (with a lower mean) when μ_*b*_ is high, or more conservative (with a higher mean) when μ_*b*_ is low. In addition, σ_*l*_ also affects the variability of confidence criteria on the processing experience distribution. When σ_*l*_ is lower (or *P*_exp_ is higher), confidence criteria are more closely distributed around the mean, suggesting that people are more likely to give extreme values for confidence ratings.

BIM assumes that the objective memory strength and processing experience for each individual follow a bivariate normal distribution with correlation coefficient ρ. We can partition the area under this bivariate normal distribution using the recall criterion and the confidence criteria on the processing experience distribution, and calculate the probability of each confidence bin for recalled and unrecalled items. Then we can derive the likelihood function, and fit BIM to empirical data using maximum likelihood estimation to estimate the free parameters (see Section S4 in Supplemental Materials, for the computation of the likelihood function for BIM with discrete confidence ratings). As in the BIM developed for continuous confidence ratings, when fitted to confidence on the discrete scale, BIM also includes five free parameters: μ_*m*_, ρ, *P*_exp_, μ_*e*_, and μ_*b*_. In addition, μ_*e*_ and μ_*b*_ are nonidentifiable and instead we estimate the parameter *M*_conf_ from empirical data.^6^

We should note that BIM is similar to SDRM when both models are fitted to empirical data of performance in recall tasks and confidence ratings on a discrete scale. Both BIM and SDRM put a recall criterion on the distribution of objective memory strength, and a set of confidence criteria on the distribution of processing experience (Jang et al., 2012). However, BIM differs from SDRM in that each confidence criterion is a free parameter in SDRM, while in BIM all of the confidence criteria on the processing experience distribution are determined by only two parameters including people’s prior beliefs about overall memory ability (μ_*b*_) and how much experience and beliefs contribute to confidence (*P*_exp_ or σ_*l*_). In addition, another difference between BIM and SDRM is that the criteria for recall and confidence are constant across trials in BIM but allowed to vary across trials in SDRM, and SDRM uses two free parameters (σ_*M*_ and σ_*C*_) to characterize the variability of these criteria. Thus, we can conclude that BIM is identical to SDRM if (a) the confidence criteria in SDRM are represented by only a scale and a location parameter rather than (*n* − 1) free parameters, and (b) the confidence and recall criteria in SDRM are not allowed to vary across trials (i.e., σ_*M*_ and σ_*C*_ are set to 0).

### BIM for Recognition Tasks

In the previous two sections, we introduced the BIM for recall tasks, in which we assume that the objective memory strength for each trial is sampled from a single distribution. However, in recognition memory tests, there are typically two types of stimuli presented during the test. For example, in an Old/New test (or Yes/No task) participants see either an old or new word in each trial. In a 2AFC test, they see an old and a new word at the same time, and the stimulus type depends on which of the two choices is the old word. Data from recognition tasks are often analyzed with SDT, which defines one of the two stimuli as “signal” and the other as “noise.” The signal and noise come from two separate distributions with a different mean of memory strength, and participants’ response in the test depends on the comparison between the memory strength for the current trial and a criterion.

**Figure 3 fig3:**
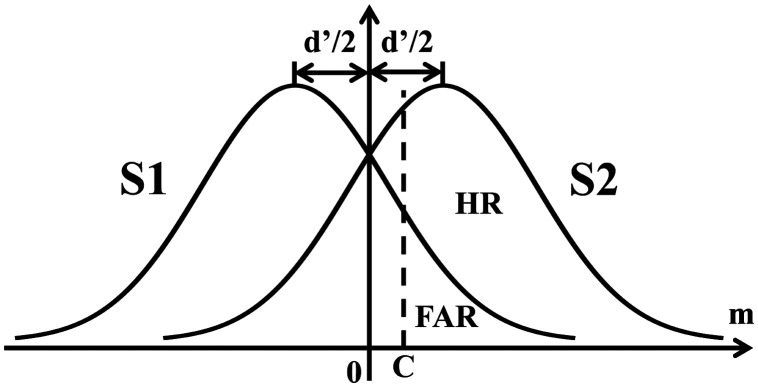
Illustration of the Application of Type I Signal Detection Theory to Recognition Memory Tasks. HR Represents Hit Rate, and FAR Represents False Alarm Rate

In this section, we introduce how BIM explains retrospective confidence ratings given after Type I response in a recognition test. We define the two types of stimuli in recognition tasks as S1 and S2, in which S1 represents noise and S2 represents the signal. We also assume that the mean of the distribution for objective memory strength is −*d*′/2 and *d*′/2 for S1 and S2, respectively, and the Type I response criterion is *C*. Participants give an S1 response when memory strength for the current trial is lower than *C*, and an S2 response when memory strength is higher than *C*. Using the hit rate (HR) and false alarm rate (FAR) from empirical data, we can easily estimate the values of *d*′ and *C* derived from SDT (see Figure 3):^7^

$$d\prime =\Phi^{-1}(HR)-\Phi^{-1}(FAR)$$ 12

$$C=-\frac{\Phi^{-1}\left(HR\right)+\Phi^{-1}\left(FAR\right)}{2}$$ 13

[Fig fig3]

After the Type I response, participants need to rate their confidence that their answer is correct. BIM assumes that when participants give confidence ratings, they are not able to directly monitor their objective memory strength. Instead, they can only infer their memory strength based on the subjective processing experience. The processing experience for each stimulus type (S1 or S2) is correlated with the objective memory strength for the same type of stimulus, and this correlation is estimated with the free parameter ρ. In addition, the mean of the processing experience distribution (μ_*e*_) may be different between the S1 and S2 stimuli. Similar to the mean of objective memory strength, the mean of processing experience should also typically be lower for S1 than S2.

For each trial, participants take a sample *e* from the processing experience distribution of S1 or S2 (depending on the stimulus type presented in the current trial) as the subjective experience. They then need to infer the probability that their Type I response is correct based on this processing experience. BIM assumes that when participants give an S1 response in the recognition test, they then aim to estimate the probability that the current stimulus is S1 when they rate their confidence. Similarly, when they give an S2 response, they aim to estimate the probability that the current stimulus is S2. However, according to the assumptions of SDT, participants do not obtain any information indicating whether the true stimulus type is S1 or S2 in each trial of a recognition task. Instead, they decide whether a stimulus belongs to S1 or S2 category simply based on the comparison between the objective memory strength and the Type I criterion *C*. BIM assumes that when participants evaluate their confidence, they similarly estimate the probability that their estimated memory strength $\hat{m}$ is higher or lower than *C* given the processing experience *e*. For example, when participants give an S1 response, their confidence is the probability that $\hat{m}$ is lower than *C* given *e*, i.e., *P* ($\hat{m}$ < *C* | *e*). In contrast, when they give an S2 response, they then estimate the probability that $\hat{m}$ is higher than *C*, i.e., *P* ($\hat{m}$ > *C* | *e*). We note that the probability estimated in the confidence rating process should only depend on participants’ response rather than the true stimulus type because participants cannot know a priori anything about the true stimulus type in the recognition test. This probability that represents participants’ confidence may be different from the probability that the stimulus in each trial belongs to S1 or S2 category based on the objective memory strength *m*, which is related to the likelihood ratio for the S1 and S2 objective strength distributions at the strength value *m* (Fleming & Daw, 2017).

In order to rate their confidence, participants then infer the posterior distribution of $\hat{m}$ given *e* via Bayesian inference. This Bayesian inference is very similar to that introduced in the BIM for recall tasks except that the prior beliefs about memory strength may be different when participants estimate the probability that the current stimulus is S1 or S2 (i.e., the probability that $\hat{m}$ is lower or higher than *C*). For example, participants may have a prior belief that the overall memory strength is lower for S1 than S2. Thus, the mean of the prior belief distribution (μ_*b*_) in the Bayesian inference may be different when the Type I response is S1 or S2. The predicted confidence for S1 and S2 responses (denoted by *r*S1 and *r*S2) given processing experience *e* can be then calculated as:

$${\text{predicted conf}}_{rS1}=P(\hat{m}<C|e)=\Phi (\frac{C-E\left(\hat{m}|e\right)}{\sqrt{\mathrm{Var}\left(\hat{m}|e\right)}})=\Phi (-\frac{e+\sigma_{l}^{2}\mu_{b\left(rS1\right)}}{\sigma_{l}\sqrt{1+\sigma_{l}^{2}}}+\frac{C\sqrt{1+\sigma_{l}^{2}}}{\sigma_{l}})$$ 14

$${\text{predicted conf}}_{rS2}=P(\hat{m}>C|e)=\Phi (\frac{E\left(\hat{m}|e\right)-C}{\sqrt{\mathrm{Var}\left(\hat{m}|e\right)}})=\Phi (\frac{e+\sigma_{l}^{2}\mu_{b\left(rS2\right)}}{\sigma_{l}\sqrt{1+\sigma_{l}^{2}}}-\frac{C\sqrt{1+\sigma_{l}^{2}}}{\sigma_{l}})$$ 15

In recognition tasks, confidence ratings can be reported on either a continuous or discrete scale. When people report confidence ratings on a 0–1 *continuous scale*, BIM assumes that the reported confidence is the sum of the predicted confidence based on the posterior probability and a noise term obtained from a normal distribution with a mean of 0 and standard deviation of .025, as in recall tasks. When confidence ratings are reported on an *n*-point discrete scale, BIM assumes that people use (*n* − 1) fixed criteria to divide the 0–1 *continuous scale* for the posterior probability into *n* intervals with equal lengths, which is also the same as in recall tasks. These (*n* − 1) criteria on the 0–1 scale for the posterior probability can be transformed into (*n* − 1) confidence criteria on the processing experience distribution for either S1 or S2 stimulus (depending on the true stimulus type in each trial). In addition, people may have different prior beliefs about memory strength (μ_*b*_) for the two stimulus types, leading to different confidence criteria on the processing experience distribution when the Type I response is S1 or S2 (see Equation 11).

In the BIM for recognition tasks, the free parameters are *P*_exp_, μ_*e*_, μ_*b*_ and ρ. In addition, instead of estimating the nonidentifiable parameters μ_*e*_ and μ_*b*_ when fitting BIM to empirical data, we estimate the mean of the confidence distribution (*M*_conf_) which is affected by both the μ_*e*_ and μ_*b*_. We already know that μ_*e*_ is different when the true stimulus type is S1 or S2, and μ_*b*_ differs between S1 and S2 response. Thus, the value of *M*_conf_ is different across the 2 (stimulus: S1 vs. S2, denoted by *sS1* and *sS2*) × 2 (response: S1 vs. S2, denoted by *r*S1 and *r*S2) conditions (see Section S1 in Supplemental Materials, for the calculation):

$$M_{\mathrm{conf}\left(sS1,rS1\right)}=\Phi (-\frac{\mu_{e\left(sS1\right)}P_{\exp}+\mu_{b\left(rS1\right)}P_{\mathrm{belief}}-C}{\sqrt{1-P_{\exp}P_{\mathrm{belief}}}})$$ 16

$$M_{\mathrm{conf}\left(sS1,rS2\right)}=\Phi (\frac{\mu_{e\left(sS1\right)}P_{\exp}+\mu_{b\left(rS2\right)}P_{\mathrm{belief}}-C}{\sqrt{1-P_{\exp}P_{\mathrm{belief}}}})$$ 17

$$M_{\mathrm{conf}\left(sS2,rS1\right)}=\Phi (-\frac{\mu_{e\left(sS2\right)}P_{\exp}+\mu_{b\left(rS1\right)}P_{\mathrm{belief}}-C}{\sqrt{1-P_{\exp}P_{\mathrm{belief}}}})$$ 18

$$M_{\mathrm{conf}\left(sS2,rS2\right)}=\Phi (\frac{\mu_{e\left(sS2\right)}P_{\exp}+\mu_{b\left(rS2\right)}P_{\mathrm{belief}}-C}{\sqrt{1-P_{\exp}P_{\mathrm{belief}}}})$$ 19

Using maximum likelihood estimation, we can fit BIM to recognition tasks with confidence ratings on either a continuous or discrete scale (see Section S2 and S4 in Supplemental Materials, for the computation of likelihood function), and estimate the free parameters including *P*_exp_, four different *M*_conf_ (for each of the four conditions) and ρ.

## Data Simulation and Parameter Recovery

In this section, we simulate data from BIM with different parameter values. We performed three different analyses with simulated data sets. First, we investigated the effect of BIM parameters on the simulated distribution of confidence ratings and memory performance. Then, we examined whether fitting BIM to simulated data could successfully recover the ground-truth parameters. Finally, we analyzed the correlation between the parameters in BIM and other computational models fitted to the simulated data.

### Confidence-Performance Joint Distribution

In this section, we investigate how values of the parameters in BIM affect the distribution of confidence ratings and memory performance. We first simulated data from the BIM for recall tasks, with each of the four free parameters in BIM set to one of five possible values: μ_*m*_ = (−1 −.5 0 .5 1), ρ = (−.8 −.4 0 .4 .8), *P*_exp_ = (.3 .4 .5 .6 .7), *M*_conf_ = (.3 .4 .5 .6 .7). For each parameter combination, we simulated 50,000 trials while recording memory performance (0 or 1) and confidence rating (on 0–1 *continuous scale*) in each trial. According to the assumption of BIM, reported confidence in each trial is the sum of the predicted confidence from BIM and a random noise from a normal distribution with a mean of 0 and standard deviation of .025. To make sure the simulated confidence ratings were within the range from 0 to 1, we set the simulated value of reported confidence in each trial to 0 if the sum of the predicted confidence and random noise was lower than 0, and 1 if the sum of the predicted confidence and noise was higher than 1. We performed kernel density estimation in MATLAB 2019a (http://www.mathworks.com) to estimate the confidence-performance joint distribution for the simulated data and examine the effect of each parameter on the shape of this distribution (Jones, 1993).

**Figure 4 fig4:**
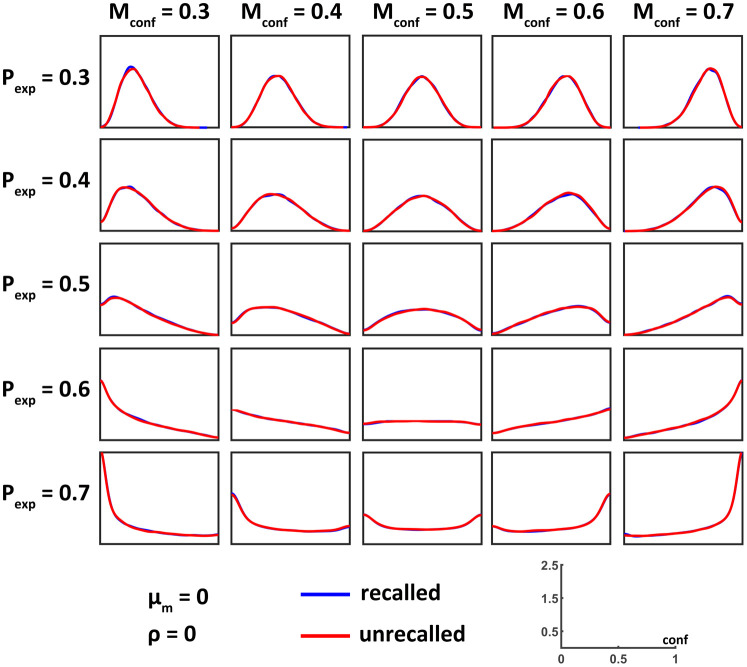
The Effect of P*_*exp*_* and M*_*conf*_* on the Confidence-Performance Joint Distribution in Recall Tasks *Note*. See the online article for the color version of this figure.

Figure 4 shows the effect of *P*_exp_ (proportion for the contribution of processing experience to confidence) and *M*_conf_ (mean of confidence distribution) on the shape of the confidence-performance joint distribution in recall tasks. The confidence distributions in the figure for recalled and unrecalled items were largely overlapped because we assumed here that participants correctly recalled half of the items (i.e., μ_*m*_ = 0) and there was no correlation between processing experience and objective memory strength (i.e., ρ = 0). We can see that the parameter *P*_exp_ mainly affected the variance of the confidence distribution, indicating that the distribution was less variable when *P*_exp_ was smaller (and vice versa). This is easy to understand according to the assumptions of BIM: BIM assumes that participants’ prior beliefs (i.e., beliefs about the overall proportion of correctly recalled items) are developed before the memory task and constant across trials. When participants rate their confidence mainly based on prior beliefs rather than processing experience, their confidence ratings should be distributed around this prior belief and the variability of the confidence distribution should be low. In contrast, the confidence distribution should have higher variability when processing experience contributes more to confidence ratings because processing experience is variable across items. We also note from Figure 4 that the parameter *M*_conf_ mainly affected the mean of confidence distribution, as expected.[Fig fig4]

**Figure 5 fig5:**
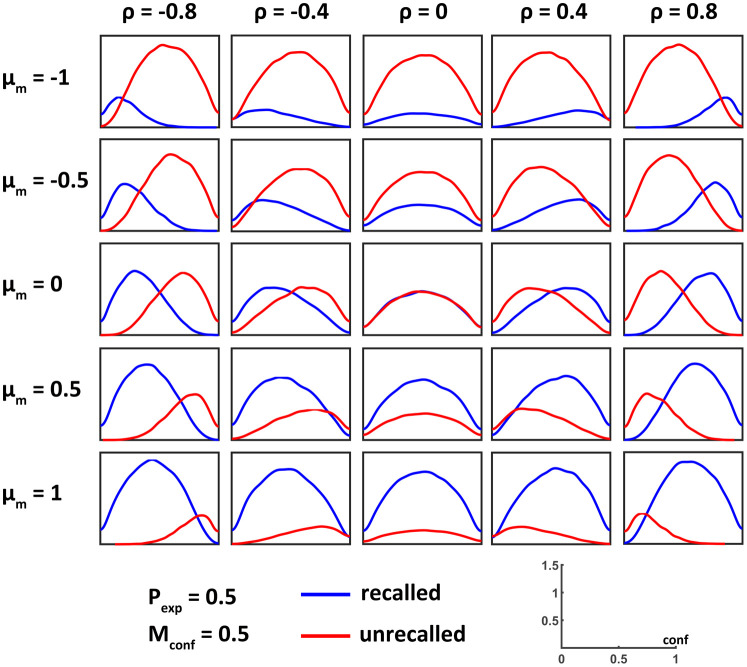
The Effect of μ*_*m*_* and ρ on the Confidence-Performance Joint Distribution in Recall Tasks *Note*. See the online article for the color version of this figure.

Figure 5 shows the effect of the parameters μ_*m*_ (mean of the objective memory strength) and ρ (correlation between objective memory strength and processing experience) on the shape of the confidence-performance joint distribution in recall tasks (here we set *P*_exp_ = .5 and *M*_conf_ = .5). We can see that μ_*m*_ mainly affected the proportion of recalled and unrecalled items: Participants should recall more items when the mean of objective memory strength is higher. On the other hand, ρ affected the distance between the confidence distributions of recalled and unrecalled items: Confidence for recalled items is higher than that for unrecalled items when there is a positive correlation between the objective memory strength and the processing experience utilized in the metamemory process (i.e., ρ > 0), and vice versa. Recalled items have higher objective memory strength than unrecalled items, and thus are also more likely to have higher strength of processing experience than unrecalled items when ρ is positive, leading to higher confidence ratings. The mean confidence for recalled and unrecalled trials should be the same when ρ = 0.[Fig fig5]

**Figure 6 fig6:**
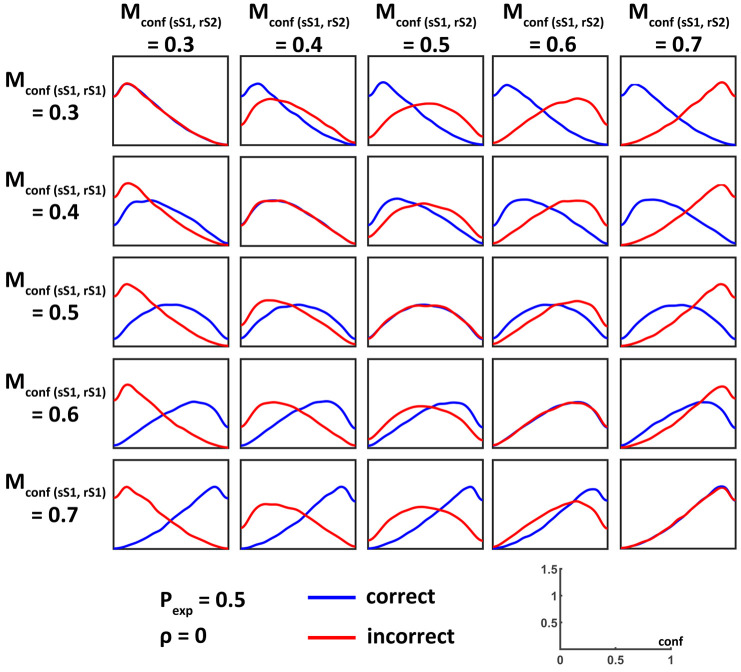
The Effect of M*_*conf (sS1, rS1)*_* and M*_*conf (sS1, rS2)*_* on the Confidence-Performance Joint Distribution Following an S1 Stimulus in Recognition Tasks *Note*. See the online article for the color version of this figure.

Next, we simulated data from the BIM for recognition tasks, in which the parameters *P*_exp_, ρ, and the four *M*_conf_ for each of the 2 (stimulus: S1 vs. S2) × 2 (response: S1 vs. S2) conditions were set to one of the five possible values described above. In addition, we set *d*′ and *C* to 0 in Type I SDT. For each parameter combination, we simulated 50,000 trials separately for S1 and S2 stimuli and recorded memory performance and confidence rating in each trial. We then performed kernel density estimation to estimate the confidence-performance joint distribution. Figure 6 shows the effect of the parameters *M*_conf (sS1, rS1)_ and *M*_conf (sS1, rS2)_ on the shape of the confidence-performance joint distribution for the S1 stimulus (here we set *P*_exp_ = .5 and ρ = 0). Consistent with our expectations, *M*_conf (sS1, rS1)_ affected the mean of the confidence distribution for correct trials (i.e., S1 response), and *M*_conf (sS1, rS2)_ affected mean confidence for incorrect trials (i.e., S2 response). Similarly, the parameters *M*_conf (sS2, rS2)_ and *M*_conf (sS2, rS1)_ separately affected mean confidence for correct and incorrect trials following an S2 stimulus.[Fig fig6]

**Figure 7 fig7:**
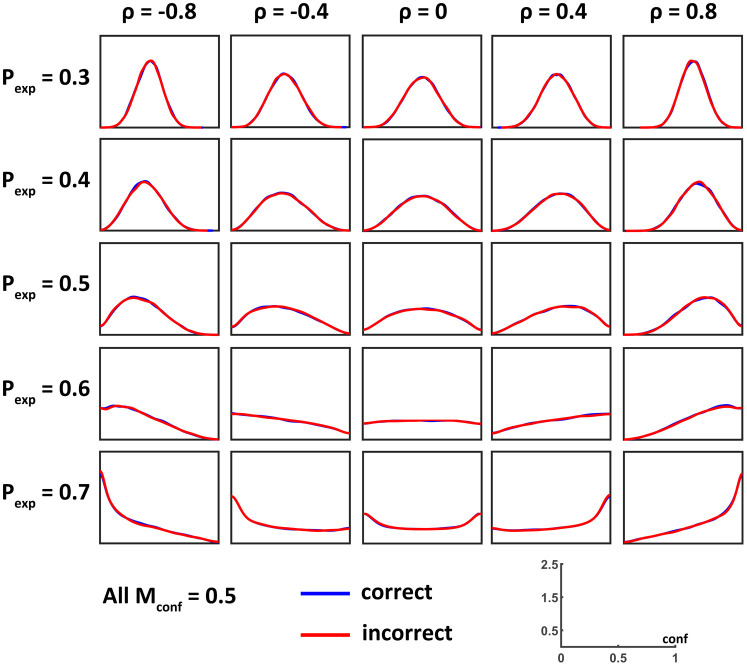
The Effect of P*_*exp*_* and ρ on the Confidence-Performance Joint Distribution in Recognition Tasks *Note*. See the online article for the color version of this figure.

Figure 7 shows the effect of the parameters *P*_exp_ and ρ on the shape of the confidence-performance joint distribution in recognition tasks (in which all four of the *M*_conf_ parameters were set to .5). Similar to the results obtained for recall tasks, the parameter *P*_exp_ mainly affected the variance of the confidence distribution in recognition tasks. However, confidence was increased for both correct and incorrect trials in recognition tasks when the parameter ρ was high (and vice versa). This result differs from that for recall tasks in which increasing ρ leads to higher confidence for correct trials but lower confidence for incorrect trials. The reason for this difference is that during the retrospective confidence rating process in recognition tasks, participants are less likely to detect an error in a Type I response when the correlation between objective memory strength and processing experience is higher. For example, when the true stimulus is S2, participants give an incorrect response (i.e., S1 response) when the objective memory strength is lower than the Type I criterion *C*. When there is a high correlation between objective memory strength and subjective processing experience, it is more likely that the processing experience is also lower than *C*, leading the model to give a high confidence rating for an incorrect response.[Fig fig7]

### Parameter Recovery

In this section, we carried out parameter recovery analyses to validate BIM, and examined whether the number of trials could affect the results of parameter recovery. We first performed parameter recovery analyses for BIM applied to recall tasks with confidence on a continuous scale. We conducted ten parameter recovery analyses with different trial numbers and simulated 1,000 data sets in each analysis. For each simulated data set, we randomly sampled the four parameters in BIM (i.e., μ_*m*_, ρ, *P*_exp_, and *M*_conf_) from uniform distributions with the following range for each parameter: (−2 2) for μ_*m*_, (−.9 .9) for ρ, (.1 .9) for *P*_exp_, and (.1 .9) for *M*_conf_. Then, we simulated data from one participant completing a block of trials of confidence ratings and memory test based on the sampled BIM parameters. The number of trials in each simulated data set is from 10 to 100 with an increment of 10 (i.e., 10, 20, 30, … 100) for the 10 parameter recovery analyses, respectively. After simulating all of the data sets, we used maximum likelihood estimation to fit BIM to each simulated data set and examined the correlation for each parameter between fitted parameter values and true values. The quality of parameter recovery was considered good if the correlation coefficient between true and recovered parameter values was higher than .75, and excellent if the correlation was higher than .9 (White et al., 2018).

**Figure 8 fig8:**
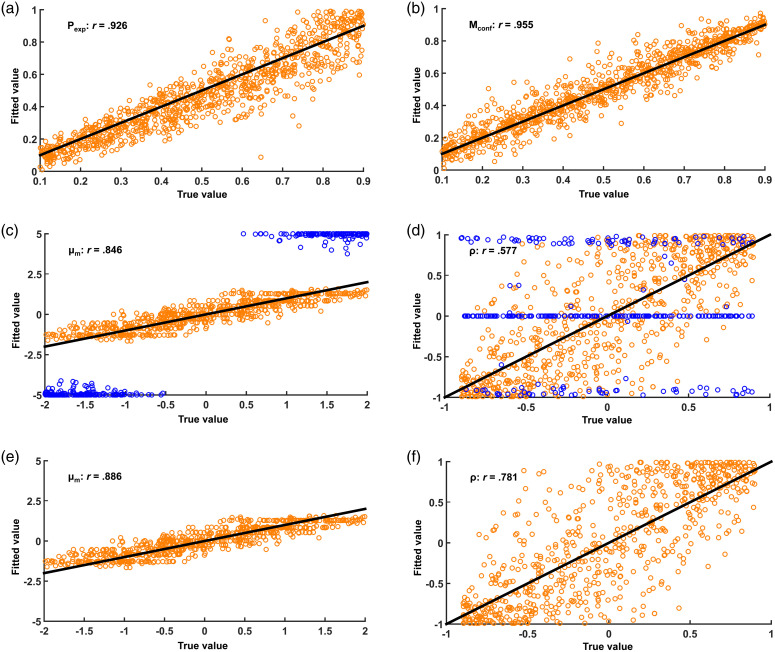
Results of Parameter Recovery for P*_*exp*_* (a), M*_*conf*_* (b), μ*_*m*_* (c), and ρ (d) in BIM for Recall Tasks With Continuous Confidence Based on Simulated Data Sets With 10 Trials Each. Values Along the Identity Line (Black Line) Indicate Good Recovery, Which is Quantified by the Correlation Between True and Fitted Parameter Values. The Correlation Between True and Fitted Value for μ*_*m*_* (e) and ρ (f) Increased After We Removed Data Sets With the Same Performance in All Trials, i.e., the Blue Dots in (c) and (d) *Note*. See the online article for the color version of this figure.

**Figure 9 fig9:**
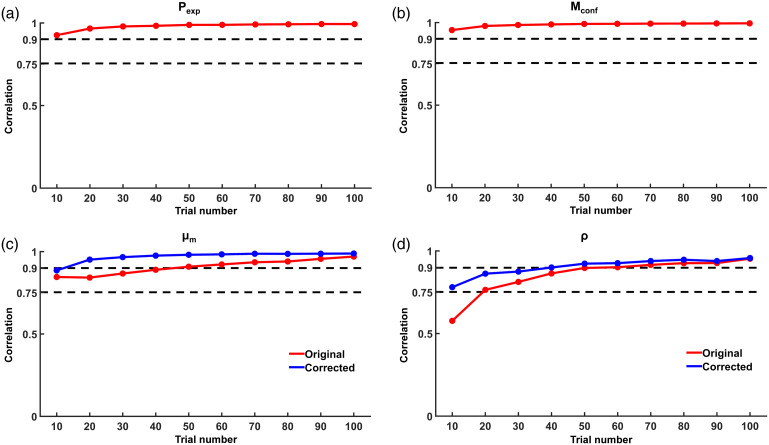
Results of Parameter Recovery for P*_*exp*_* (a), M*_*conf*_* (b), μ*_*m*_* (c), and ρ (d) in BIM for Recall Tasks With Continuous Confidence as a Function of Number of Trials. The Two Dashed Lines Represent That the Correlation Between True and Fitted Parameter Values is .75 and .9, Respectively. The Original Correlation (For All Simulated Data Sets) and Corrected Correlation (After We Removed the Data Sets With Same Performance for All Trials) are Shown for the Parameters μ*_*m*_* and ρ *Note*. See the online article for the color version of this figure.

Results revealed that parameter recovery for *P*_exp_ (*r* = .926) and *M*_conf_ (*r* = .955) was excellent with only 10 trials for each simulated data set (see Figure 8a,b). The recovery for μ_*m*_ (*r* = .846) and ρ (*r* = .577) was worse than *P*_exp_ and *M*_conf_, especially for the parameter ρ which could not be successfully recovered for all simulated data sets with only 10 trials (see Figure 8c,d). One important reason for this difference is that there were some simulated data sets in which memory performance for all trials was the same (0 or 1), and the estimations of μ_*m*_ and ρ in these data sets were very inaccurate (see the blue dots in Figure 8c,d). When these data sets were removed, the parameter recovery was improved for both μ_*m*_ (*r* = .886) and ρ (*r* = .781) (see Figure 8e,f). Thus, we only needed 10 trials to successfully recover all of the 4 parameters in BIM, and the recovery for each parameter was better with a larger number of trials (see Figure 9). When the number of trials is equal to or higher than 50, the recovery for all parameters was excellent (*r*_s_ > .9).[Fig fig8][Fig fig9]

Next, we performed parameter recovery analyses for BIM applied to recall tasks with discrete confidence ratings (using 3-point and 7-point scale as examples; see Figure S1 in Supplemental Materials). The results were similar to those for BIM with continuous confidence, except that all of the four parameters in BIM (μ_*m*_, ρ, *P*_exp_, and *M*_conf_) could only be successfully recovered (*r*_s_ > .75) when using no less than 20 trials for confidence rated on a 7-point scale, and 30 trials for confidence on a 3-point scale.

We then conducted parameter recovery analyses for BIM applied to recognition tasks with continuous confidence. The six parameters in BIM, including *P*_exp_, ρ, and four *M*_conf_ in the 2 (stimulus: S1 vs. S2) × 2 (response: S1 vs. S2) conditions were randomly sampled from the same ranges as in previous analyses. In addition, for each simulated data set, we randomly sampled the two Type I SDT parameters (*d*′ and *C*) from the following ranges: (−3 3) for *d*′, and (−1 1) for *C*. Based on the parameters in BIM and SDT, we conducted ten parameter recovery analyses with trial numbers ranging from 10 to 100 in each data set, and simulated 1,000 data sets in each analysis. In each simulated data set, the stimulus was S1 for half of the trials and S2 for the other half. We generated the Type I response for each trial based on the SDT parameters and the confidence rating (on a 0–1 *continuous scale*) for each trial from BIM. After simulating all of the data sets, we fit BIM to each data set and examined the correlation for each parameter in BIM between fitted and true values. When computing the correlation between true and recovered values for *M*_conf_ in each of the 2 (stimulus: S1 vs. S2) × 2 (response: S1 vs. S2) conditions, we removed the simulated data sets in which there were no trials in the same condition because the estimation of *M*_conf_ in these data sets is likely to be inaccurate.

**Figure 10 fig10:**
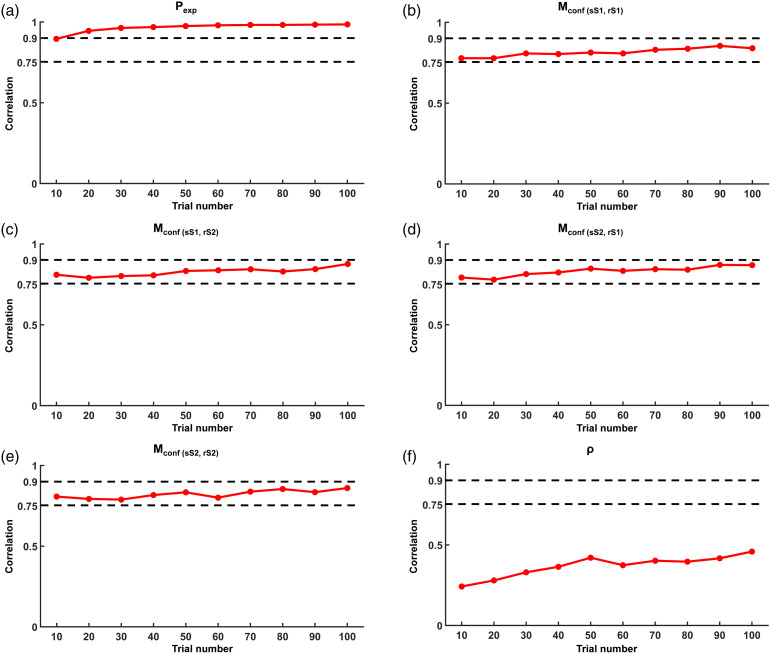
Results of Parameter Recovery for P*_*exp*_* (a), M*_*conf*_* in 2 (Stimulus: S1 vs. S2) × 2 (Response: S1 vs. S2) Conditions (b–e), and ρ (f) in BIM for Recognition Tasks With Continuous Confidence as a Function of Number of Trials. The Two Dashed Lines Represent That the Correlation Between True and Fitted Parameter Values is .75 and .9, Respectively *Note*. See the online article for the color version of this figure.

Results revealed that we only needed 10 trials to recover *P*_exp_ and the four *M*_conf_ (*r*_s_ > .75; see Figure 10a–e). However, the parameter ρ could not be successfully recovered when the trial number in each simulated data set was between 10 and 100 (see Figure 10f). In fact, we were not able to recover ρ even with 500 trials in each data set (*r* = .489). One possible explanation is that the effect of ρ on model predictions about confidence ratings might be partly mimicked by the effect of *M*_conf_ because both parameters could affect the overall mean of confidence ratings (see Figures 6 and 7), and thus it might be difficult to recover the true value of ρ.[Fig fig10]

Finally, we performed parameter recovery analyses for BIM applied to recognition tasks with discrete confidence ratings (see Figure S2 in Supplemental Materials). The parameter *P*_exp_ could be successfully recovered (*r* > .75) with 10 trials for a 7-point confidence scale and 20 trials for a 3-point scale. In addition, the correlation between true and recovered values for each *M*_conf_ was close to .75, and all of the four *M*_conf_ parameters could be successfully recovered with 90 trials for a 3-point scale and 30 trials for a 7-point scale. Furthermore, similar to BIM for recognition tasks with continuous confidence, the parameter ρ in BIM for recognition with discrete confidence could not be successfully recovered.

In summary, when we fit BIM to confidence ratings on a continuous scale in either recall or recognition tasks, we only need a small number of trials (e.g., 10 trials) to accurately estimate all of the BIM parameters except for ρ in BIM for recognition tasks. When confidence is on a discrete scale, we may need more trials to estimate the parameters. For example, 20–30 trials are required when we aim to estimate all of the parameters in BIM for recall tasks with discrete confidence ratings. In addition, when we fit BIM to recognition tasks with discrete confidence ratings, we only need 10–20 trials if we focus on the parameter *P*_exp_. However, a larger number of trials may be needed (e.g., more than 90 trials) if we aim to estimate all of the four *M*_conf_ parameters. Furthermore, we do not recommend analyzing the fitted value for the parameter ρ in BIM for recognition because it is difficult to estimate the true value of ρ in recognition tasks.

### BIM and Other Computational Models

In this section, we investigated the correlation between parameters in BIM and other metrics of metacognition, including Goodman–Kruskal Gamma, Area under the Type II ROC curve (AUROC), and parameters obtained from the SDRM and meta-*d*′ models (Fleming & Lau, 2014; Jang et al., 2012; Maniscalco & Lau, 2012; Nelson, 1984). Examining the relationship between parameters in BIM and other metrics can help us further understand the computational processes underlying BIM, and how changes in BIM parameters may affect confidence distributions and metamemory accuracy.

Goodman–Kruskal Gamma is a correlation measure for the relative accuracy of metamemory (also called resolution or metacognitive sensitivity) and is widely used in the metamemory literature (Fleming & Lau, 2014; Nelson, 1984). Gamma reflects the nonparametric correlation between confidence rating and memory performance, and a person can be ascribed a higher metamemory accuracy when the Gamma correlation is higher. Area under the Type II ROC curve (AUROC) is another nonparametric measure of metamemory accuracy based on receiver-operating characteristic (ROC) analysis (Fleming & Lau, 2014; Higham & Higham, 2018). To compute AUROC, we first separate high-confidence and low-confidence trials based on each confidence level and identify the hits (correctly answered trials with high confidence) and false alarm trials (incorrectly answered trials with high confidence) in the Type II task (i.e., the confidence rating task). Then a set of HRs and FARs can be computed and plotted on a Type II ROC curve, and the area under this ROC curve can be estimated using the trapezoidal rule (Higham & Higham, 2018). AUROC is preferred to the Gamma correlation as a metric of metacognitive accuracy because AUROC is not systematically affected by bias in confidence ratings (Fleming & Lau, 2014).

SDRM has been introduced in previous sections. In SDRM, a participant’s confidence rating is assumed to be based on a comparison between processing experience (which is correlated with objective memory strength) and a set of confidence criteria, while memory performance is based on the comparison between objective memory strength and a criterion for recall. The criteria for recall and confidence ratings in SDRM are allowed to shift across trials. SDRM contains (*n* + 3) parameters (in which *n* is the total available levels of confidence ratings), including (*n* − 1) confidence criteria (*C*_conf_), a criterion for recall (*C*_M_), standard deviations for the shift of criteria for confidence ratings (σ_*C*_) and recall (σ_*M*_), and the correlation coefficient (ρ) between the distributions of processing experience and objective memory strength (Jang et al., 2012). However, σ_*M*_ and σ_*C*_ are typically nonidentifiable when SDRM is fitted to data in a single experimental condition. In order to make SDRM identifiable for a single condition, σ_*M*_ and σ_*C*_ in SDRM can be set to 0 to prevent variation of criteria across trials. This restricted SDRM is more similar to BIM because the confidence criteria are also not allowed to vary across trials in BIM for discrete confidence ratings.

The meta-*d*′ model is a widely used SDT-based model of metacognitive judgments in two-choice perceptual and memory tasks (e.g., recognition test; Fleming & Lau, 2014; Maniscalco & Lau, 2012). The model is fit to the stimulus (S1 vs. S2) × response (S1 vs. S2) × confidence matrix. Meta-*d*′ reflects participants’ metacognitive sensitivity (i.e., whether participants can discriminate between correct and incorrect responses), and is in the same units as Type I performance *d*′. Thus, meta-*d*′ and *d*′ can be directly compared, and meta-*d*′/*d*′ is often used as a measurement of the efficiency of participants’ metacognition given a particular level of task performance (Fleming & Lau, 2014). The meta-*d*′ model contains (2 *n* − 1) parameters (in which *n* is the total available levels of confidence ratings), including (*n* − 1) confidence criteria for S1 response (*C*_rS1_), (*n* − 1) confidence criteria for S2 response (*C*_rS2_) and meta-*d*′.

Based on simulations of confidence distributions with different parameter values in BIM (see Figures 4–7), we expected that the parameter *P*_exp_ in BIM should mainly affect the variance of the confidence distribution, which should be reflected in the variance or standard deviation of the confidence criteria in other models such as SDRM and the meta-*d*′ model. The parameter *M*_conf_ affects the mean of confidence distribution and should relate to the mean of confidence criteria in other models. In addition, the parameter μ_*m*_ in BIM for recall tasks reflects objective memory strength and should be correlated with the parameters representing memory performance. The parameter ρ for recall tasks determines the difference between confidence for recalled and unrecalled trials and thus might be related to different indices of metamemory accuracy.

Here we simulated memory performance and confidence rating data (on a 7-point scale) to investigate the relationship between parameters in BIM and other metrics of metacognition. We first examined the relationship between parameters in BIM for recall tasks and other metrics, including Gamma, AUROC, and parameters of SDRM. We sampled 1,000 different sets of parameters in BIM from the same ranges as used in parameter recovery analysis. For each parameter set, we simulated data of one participant completing 500 trials of confidence ratings (on a 7-point scale) and recall test. We simulated 500 trials for each data set to ensure that the parameters in all models could be accurately estimated. We then used maximum likelihood estimation to fit BIM and SDRM to each simulated data set and calculated Gamma and AUROC for each data set. When fitting SDRM to the data sets, we set σ_*M*_ and σ_*C*_ to 0 to remove the variation in recall and confidence criteria across trials, which makes all of the other parameters in SDRM identifiable. We examined the nonparametric Spearman correlation coefficient between parameters in BIM and other models, and considered there was a close relationship between two parameters only when the two parameters showed a large correlation (*r* > .5 or *r* < −.5; Cohen, 1977).^8^ For SDRM, we also computed the mean and standard deviation of the confidence criteria and examined the correlation between these variables and the parameters in BIM.

The correlation coefficients between parameters in BIM for recall tasks and other metrics are shown in Table 1. Results revealed that parameter *P*_exp_ in BIM was negatively correlated with the standard deviation of confidence criteria in SDRM. Confidence criteria are close to each other when the standard deviation is smaller, suggesting that participants are more likely to give extreme values rather than medium values when rating their confidence. This is consistent with the simulated confidence distributions (see Figure 4), showing that participants are more willing to give extreme confidence ratings when processing experience contributes more to metamemory. Parameter *M*_conf_ in BIM was negatively correlated with mean confidence criteria in SDRM, suggesting that people set liberal confidence criteria when the mean of confidence distribution is high. Parameter μ_*m*_ in BIM was negatively correlated with the recall criterion in SDRM, showing that a liberal recall criterion leads to high memory performance. Parameter ρ in BIM positively correlated with different indices of metamemory accuracy, including Gamma, AUROC, and ρ in SDRM. In addition, the other three parameters in BIM did not significantly correlate with metamemory accuracy indices, indicating that only the correlation between memory strength and processing experience can predict metamemory accuracy in recall tasks.[Table tbl1]

Next, we examined the relationship between BIM for recognition tasks and other metrics, including Gamma, AUROC, and parameters of the meta-*d*′ model. We sampled 1,000 different sets of parameters in BIM and SDT from the same ranges as in the parameter recovery analyses in the previous section. For each parameter set, we simulated data of 250 trials following an S1 stimulus and 250 trials following an S2 stimulus. We then fit BIM and the meta-*d*′ model to each simulated data set and calculated Gamma and AUROC for each data set. When fitting the meta-*d*′ model to simulated data sets, we applied padding correction to ensure all of the parameters in the meta-*d*′ model could be successfully estimated (Fleming, 2017; Maniscalco & Lau, 2012). We also computed the mean and standard deviation of the confidence criteria in the meta-*d*′ model separately for S1 and S2 responses and examined the correlation between these variables and the parameters in BIM. In addition, we did not analyze the parameter ρ in BIM for recognition tasks because it is difficult to estimate the true value of ρ.

The correlation coefficients between parameters in BIM for recognition tasks and other metrics are shown in Table 2. Parameter *P*_exp_ in BIM was negatively correlated with the standard deviation of confidence criteria in the meta-*d*′ model, as expected. In contrast, none of the parameter *M*_conf_ showed a large correlation with the mean confidence criteria in the meta-*d*′ model, which diverges from our hypothesis. However, we noticed that the correlation between each *M*_conf_ and the mean confidence criteria depended on the Type I performance in the recognition test (i.e., *d*′). When *d*′ was higher than 0, *M*_conf (sS1, rS1)_ and *M*_conf (sS2, rS2)_ largely correlated with the mean confidence criteria in the meta-*d*′ model for S1 and S2 responses, respectively (*r* = .521 and −.583). This is because *M*_conf (sS1, rS1)_ and *M*_conf (sS2, rS2)_ represent the mean confidence for correct trials. There should be more correct than incorrect trials in the data sets when *d*′ is higher than 0, and the mean confidence criteria in the meta-*d*′ model should mainly correlate with *M*_conf_ for correct trials. Similarly, when *d*′ was lower than 0, *M*_conf (sS2, rS1)_ and *M*_conf (sS1, rS2)_ showed large correlations with the mean confidence criteria in the meta-*d*′ model for S1 and S2 responses, respectively (*r* = .589 and −.553) because the mean confidence criteria should mainly correlate with *M*_conf_ for incorrect trials. In addition, neither *P*_exp_ or any of the *M*_conf_ showed large correlations with indices of metamemory accuracy such as Gamma, AUROC or meta-*d*′.[Table tbl2]

To further investigate the relationship between the parameters in BIM and other models, we also simulated data generated by either the SDRM or the meta-*d*′ model and examined the correlation between the parameters in the generative model and BIM. The results were very similar to those reported above (see Section S5 and Tables S1–2 in Supplemental Materials).

## Fitting BIM to Empirical Data

In this section, we provide examples of fitting BIM to empirical data sets in four studies. In Studies 1–3, we fit BIM to experiments on judgments of learning (JOLs). We examined if the results from BIM in Studies 1–2 were consistent with previous theories of metamemory. In Study 3, we conducted a stronger test of the assumption about the Bayesian inference process in BIM. Furthermore, we compared the fit of BIM and SDRM to data in Studies 1–3 to see which model could better account for the data. Finally, in Study 4, we showed an example of how to fit BIM to data from a recognition memory test with retrospective confidence ratings.

### Study 1: JOLs Versus Prestudy JOLs

A JOL is a prediction of the likelihood of remembering studied materials in a subsequent memory test (Nelson & Narens, 1990). In a typical study on JOLs, participants need to predict their memory performance after they finish learning each item (Bjork et al., 2013; Nelson & Narens, 1990). Results from many studies suggest that poststudy JOLs rely on both the current experience in processing each item and participants’ prior beliefs about their overall memory ability (Frank & Kuhlmann, 2017; Hu et al., 2015; Mueller, Dunlosky, Tauber, et al., 2014; Su et al., 2018; Undorf et al., 2017; Witherby & Tauber, 2017; Yang, Huang, et al., 2018). However, in some studies, researchers asked participants to guess the likelihood of remembering each item before they see the item. This type of metamemory judgment is called prestudy JOL (Mueller, Dunlosky, Tauber, et al., 2014; Mueller et al., 2016; Witherby & Tauber, 2017). Participants are not able to see the item prior to making a prestudy JOL, and have to largely rely on their prior belief about overall memory ability to guess the memory performance for each item (Mueller, Dunlosky, Tauber, et al., 2014). Thus, compared with poststudy JOLs, prestudy JOLs should be based more on prior beliefs and less on processing experience.

In this study, we asked participants to give post- or prestudy JOLs to a list of word pairs. We used BIM to investigate the contribution of processing experience and prior beliefs to post- and prestudy JOLs. Based on previous studies, we expected that the contribution of processing experience (i.e., *P*_exp_) should be higher for post- than prestudy JOLs. In addition, we expected that the correlation between processing experience and objective memory strength (i.e., ρ) should also be higher for post- than prestudy JOLs. It is possible that when giving prestudy JOLs, participants may use the processing experience for previously learned items to infer the memory performance of the following item (Mueller et al., 2013). However, the processing experience for previous items should not accurately predict the objective memory strength for the current item. In contrast, when giving poststudy JOLs, participants utilize their experience in processing the current item, which should more accurately predict the objective memory strength.

Another purpose of this study was to compare the fit of BIM and SDRM to empirical data. We first compared the empirical JOL-performance joint distribution in two experimental conditions (post- and prestudy JOLs) and the predictions from BIM and SDRM, and then examined which model could better account for the data.

#### Participants

The participants included 28 students from Beijing Normal University (6 men; age: *M* = 21.21 years, *SD* = 2.44). Each participant was tested individually, and written informed consent was obtained from all participants. This sample size was similar to previous studies of metacognition using computational modeling (Fleming et al., 2018; Hu et al., 2019). All procedures were approved by the local ethics committee.

#### Materials

The materials consisted of 50 Chinese word pairs. Each word pair contained one cue word and one target word. All of the words were two-character words that were from the Chinese word database by Cai and Brysbaert (2010). The word frequency was between .03 and 46.2 per million words. Before the experiment, 238 raters used a four-point rating scale to evaluate the semantic associations of all the word pairs. On the four-point scale, 1 represented “very unrelated” and 4 represented “very related”. The semantic associations of all word pairs were between 1 and 2. Another six word pairs were used in the practice stage.

#### Procedure

The experiment consisted of three phases: Learning (in which pre- and poststudy JOLs were made), distractor task, and memory test. In the learning phase, participants were required to learn 50 word pairs with a 3 s presentation time for each word pair. Word pairs were randomly divided into two experimental conditions (post- and prestudy JOLs), and each condition contained 25 pairs. For the pairs in the poststudy JOL condition, participants were instructed to rate the likelihood of recalling the target word in a later memory test given the cue word (i.e., JOL) immediately following the presentation of each pair. Participants gave their JOLs on a sliding scale from 0 to 100. Arbitrary scale values of 0, 20, 40, 60, 80, and 100 were marked at equal spacings. The initial cursor position on each trial was randomly jittered around the midpoint of the scale (±12% of scale length). Participants used the left or right arrow key to move the cursor up or down the scale. The final cursor position was recorded as a continuous variable on each trial. For the word pairs in the prestudy JOL condition, participants guessed the likelihood of recalling the target word before learning each pair. They gave prestudy JOLs in the same way as poststudy JOLs. Word pairs were presented in a pseudorandom order in which no more than three pairs from the same experimental condition were presented consecutively.

After the learning phase, participants engaged in a 1-min arithmetic distractor task. They were then given a recall test. The computer screen showed a cue word in each trial, and participants needed to type the target word in the same pair. There was no time limit for the test of each pair, and participants could choose to skip the test for a trial if they could not recall the target word.

#### Results

The mean JOL magnitude was significantly higher for post- than prestudy JOLs, *t*(27) = 3.79, *p* = .001, Cohen’s *d* = .72, η_p_^2^ = .35, indicating that participants were more confident about their memory after they saw the word pair. However, the proportion of recalled word pairs did not differ between the post- and prestudy JOL conditions, *t*(27) = .88, *p* = .389, Cohen’s *d* = .17, η_p_^2^ = .03, suggesting that the type of JOL did not affect memory performance. We then divided the 0–100 *continuous scale* for JOLs into seven equal-length bins (i.e., 0−14, 14−28, etc.), and used AUROC to quantify the accuracy of binned JOLs in the pre- and poststudy condition (Fleming & Lau, 2014).^9^ We found that although metamemory accuracy was slightly higher for post- than prestudy JOLs, this difference did not reach significance, *t*(27) = 1.52, *p* = .140, Cohen’s *d* = .29, η_p_^2^ = .08 (see Table 3).[Table tbl3]

We next fit BIM for each participant separately in the post- and prestudy JOL conditions (see Table 4). JOLs were converted into a percentage scale, and memory performance was set to 0 for unrecalled trials and 1 for recalled trials. We removed the parameters μ_*m*_ and ρ in the prestudy JOL condition for one participant because the memory performance was the same (i.e., 1) for all trials. Then we used paired-sample *t*-tests to compare *P*_exp_ and *M*_conf_, and linear mixed effect models to compare μ_*m*_ and ρ between two conditions because linear mixed effect models can properly handle missing data within participants (Hu et al., 2019).^10^ We found that the contribution of processing experience (*P*_exp_) was significantly higher for post- than prestudy JOLs, *t*(27) = 4.39, *p* < .001, Cohen’s *d* = .83, η_p_^2^ = .42, indicating that participants relied more on processing experience (rather than prior beliefs about their overall memory ability) when making poststudy compared with prestudy JOLs. In addition, the mean of the confidence distribution (*M*_conf_) was higher for post- than prestudy JOLs, *t*(27) = 3.80, *p* = .001, Cohen’s *d* = .72, η_p_^2^ = .35. The mean of objective memory strength (μ_*m*_) did not differ between two conditions, *t*(25.84) = .54, *p* = .596, η_p_^2^ = .01. These results are consistent with previous analyses of mean JOL magnitude and recall performance. Finally, the correlation between processing experience and objective memory strength (ρ) was also higher for post- than prestudy JOLs, *t*(25.81) = 2.30, *p* = .030, η_p_^2^ = .17, suggesting that the processing experience utilized in poststudy JOLs could better predict the memory performance than that in prestudy JOLs.[Table tbl4]

**Figure 11 fig11:**
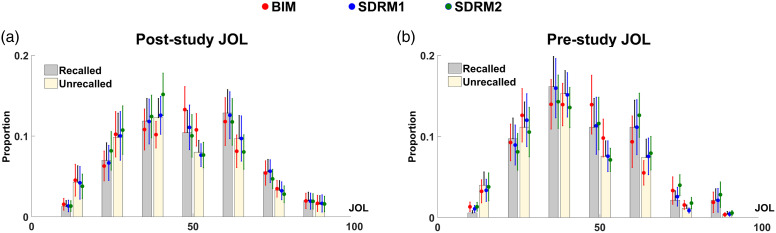
The JOL-Performance Joint Distribution for Poststudy JOLs (a) and Prestudy JOLs (b) Condition in Study 1. The Red, Blue, and Green Points Represent the Predictions From BIM, SDRM1, and SDRM2, Respectively. Error Bars Represent Standard Errors *Note*. See the online article for the color version of this figure.

Finally, we compared the fit of BIM and SDRM to the data. We first divided the percentage scale for JOLs into seven equal-length bins, and then fit BIM and SDRM to binned JOLs (i.e., on a 7-point scale) and recall performance in each experimental condition. When fitting SDRM to data, we set the standard deviations for recall and confidence criteria (i.e., σ_*M*_ and σ_*C*_) to 0 to ensure SDRM was identifiable for each condition and more similar to BIM in which the variation of criteria across trials is not allowed. We should note that the difference in mathematical structure between BIM and the current version of SDRM is that each confidence criteria in SDRM is a free parameter, while all of the confidence criteria on the processing experience distribution in BIM are determined by only two parameters (*P*_exp_ and *M*_conf_). We also fit another restricted version of SDRM in which the confidence criteria in one experimental condition were the rescaled criteria in the other condition adjusted by a scale parameter β. This restricted SDRM is very similar to the Model 4c in Jang et al. (2012), except that the parameters σ_*M*_ and σ_*C*_ were set to 0 in the current model. Here, we denote the SDRM without and with the restriction on the confidence criteria via parameter β as SDRM1 and SDRM2, respectively. The JOL-performance joint distribution for the data and model predictions is shown in Figure 11. All of the three models could fit the overall pattern of JOL-performance distribution in both post- and prestudy JOL conditions.[Fig fig11]

To further compare the fit of the three models while accounting for the difference in model complexity, we next computed the cross-validated (CV) log-likelihood for each model using leave-one-out cross-validation. For each participant, we used one trial as the test data set and the other trials as the training data set. We separately fit each of the three models to the training data set and calculated the log-likelihood for the left-out trial based on the fitted parameters. We could obtain the CV log-likelihood of each trial for a participant using this method, and then summed the CV log-likelihood of all trials from all participants separately for each of the three models. Results revealed that the aggregated CV log-likelihood of BIM (−3993.46) was higher than that of SDRM1 (−7321.53) and SDRM2 (−5455.34), suggesting that BIM could better predict the data than SDRM.

#### Discussion

The results from BIM support our hypothesis that processing experience should contribute more to post- than prestudy JOLs (Mueller, Dunlosky, Tauber, et al., 2014), indicating that BIM is consistent with previous theories on metamemory. We also found that the overall proportion for the contribution of processing experience to prestudy JOLs (i.e., *P*_exp_) across participants was .24 (see Table 4), which is considerably higher than 0. One possible explanation is that participants’ prestudy JOL for each trial might rely not only on prior beliefs about memory ability, but also the processing experience in previous trials (Mueller et al., 2013). In addition, our results showed that the processing experience utilized in poststudy JOLs could predict objective memory strength more accurately than that in prestudy JOLs, suggesting that the processing experience of current items is more closely related to objective memory than that of previous items.

Another important result from Study 1 is that BIM could better predict the data than SDRM1 and SDRM2 (based on the CV log-likelihood), although all of the three models could fit the overall pattern of JOLs and memory performance. One possible explanation for the difference in CV log-likelihood between BIM and SDRM is that BIM may be a more parsimonious model than SDRM. While each confidence criterion in SDRM is a free parameter, the confidence criteria in BIM for discrete confidence ratings are decided by only a location and a scale parameter (see Equation 11). This difference in model complexity may be accounted for by the CV log-likelihood, leading to a better prediction of data by BIM than SDRM.

### Study 2: Delayed JOL Effect

In many studies on JOLs, participants are asked to make JOLs immediately after learning each item (e.g., Finn & Metcalfe, 2008; Frank & Kuhlmann, 2017; Hu et al., 2015; Koriat, 1997; Mueller, Dunlosky, Tauber, et al., 2014). However, there may also be a delay between when learning occurs and the JOL is made. For example, in some studies participants were required to make a JOL for each item after all items were learned. This type of JOL is called delayed JOL (Nelson & Dunlosky, 1991; Rhodes & Tauber, 2011). Previous studies have indicated that delayed JOLs show significantly higher metamemory accuracy than immediate JOLs, known as the delayed JOL effect (Hu et al., 2016; Nelson & Dunlosky, 1991; Rhodes & Tauber, 2011; Spellman & Bjork, 1992; Weaver & Kelemen, 1997).

While immediate JOLs have been shown to rely on both processing experience and prior beliefs, researchers suggest that delayed JOLs are mainly based on processing experience incidental to the JOL process (Nelson & Dunlosky, 1991; Rhodes & Tauber, 2011; Spellman & Bjork, 1992). For example, Spellman and Bjork suggest that when rating the likelihood of recalling an item after a delay, participants first try to retrieve the item from their long-term memory. Whether participants believe they could recall the item in a later test depends on this retrieval attempt: They should give a high JOL when they successfully and fluently retrieve this item (and vice versa). Thus, compared to immediate JOLs, processing experience should contribute more to delayed JOLs.

In this study, we used BIM to reanalyze the data in our previous study on the delayed JOL effect (Hu et al., 2016). Based on the theory of delayed JOLs outlined above (Rhodes & Tauber, 2011; Spellman & Bjork, 1992), we expected that the contribution of processing experience to JOLs (i.e., *P*_exp_) should be higher for delayed than immediate JOLs. We also expected that the correlation between processing experience and objective memory strength (i.e., ρ) should also be higher for delayed than immediate JOLs. This is because participants mainly rely on information in short-term memory when making immediate JOLs, while they rely on long-term memory when making delayed JOLs. Compared with short-term memory, the information in long-term memory is more likely to accurately predict performance in a later test (Nelson & Dunlosky, 1991). In addition, we compared the fit of BIM and SDRM to the data for immediate and delayed JOLs.

#### Participants

Hu et al. (2016) contain three experiments. There were 28 participants (11 men) in Experiment 1, 34 participants (11 men) in Experiment 2, and 25 participants (5 men) in Experiment 3.

#### Materials

The materials consisted of 52 Chinese word pairs. All of the words were two-character words that were from the Chinese word database by Cai and Brysbaert (2010). The word frequency was between .006 and 46.2 per million words. The semantic associations of the word pairs were between 1.3 and 1.9 on a 4-point scale.

#### Procedure

Each experiment consisted of four phases: Learning (in which immediate JOLs were made), delayed JOLs, a distractor task, and memory test (except for Experiment 3 in which there was also a lexical decision task). In the learning phase, participants were required to learn 52 word pairs with 4 s presentation time for each word pair. The first and last two pairs were buffering pairs and were not included in the JOL and recall phases. From the remaining 48 pairs, half (i.e., 24 pairs) were randomly selected for immediate JOLs. Immediately following the presentation of these pairs, participants were instructed to orally report a number between 0 and 100 indicating their JOL. After learning all of the pairs, participants were required to make JOLs for the remaining 24 pairs with only the cue words presented on the screen (i.e., delayed JOL). After the delayed JOL phase, participants engaged in an arithmetic distractor task. They were then given a recall test. During the immediate and delayed JOL phases, cue words were presented in either 70-pt or 9-pt font size. In the data analyses here, we combined the trials with different font sizes (see Hu et al. (2016), for further details of the experimental procedure).

#### Results

The mean JOL magnitude and recall performance did not differ between immediate and delayed JOLs in Experiment 1, *t*_s_ < 1.3, *p*_s_ > .2, Cohen’s *d* < .3, η_p_^2^ < .06. However, in Experiments 2 and 3 both the mean JOL magnitude and recall performance was higher for immediate than delayed JOLs, *t*_s_ > 2.6, *p*_s_ < .05, Cohen’s *d* > .4, η_p_^2^ > .17. This consistency in JOL magnitude and recall performance across two conditions confirms that participants could accurately predict the overall pattern of memory performance. We then used AUROC to quantify the accuracy of JOLs, and found that in all of the experiments JOL accuracy was significantly higher for delayed than immediate JOLs, *t*_s_ > 10.2, *p*_s_ < .001, Cohen’s *d* > 1.9, η_p_^2^ > .79, replicating the delayed JOL effect found in previous studies (see Table 3).

We next fit BIM for each participant separately in immediate and delayed JOL conditions (see Table 4).^11^ In all of the three experiments, the contribution of processing experience to JOLs (i.e., *P*_exp_) was higher for delayed than immediate JOLs, *t*_s_ > 7.6, *p*_s_ < .001, η_p_^2^ > .68, showing that participants relied more on processing experience during delayed JOLs. We also found that the correlation between processing experience and objective memory strength (ρ) was higher for delayed than immediate JOLs in all of the experiments, *t*_s_ > 7.4, *p*_s_ < .001, η_p_^2^ > .46, suggesting that the information utilized in delayed JOLs (such as the results of retrieval attempt) could better predict memory performance. In addition, the mean of the confidence distribution (*M*_conf_) and objective memory strength (μ_*m*_) did not differ between immediate and delayed JOLs in Experiment 1, *t*_s_ < 1.0, *p*_s_ > .3, Cohen’s *d* < .2, η_p_^2^ < .05, but were significantly higher for immediate than delayed JOLs in Experiments 2 and 3, *t*_s_ > 2.4, *p*_s_ < .05, η_p_^2^ > .15, which is consistent with previous analyses on mean JOL magnitude and recall performance.

**Figure 12 fig12:**
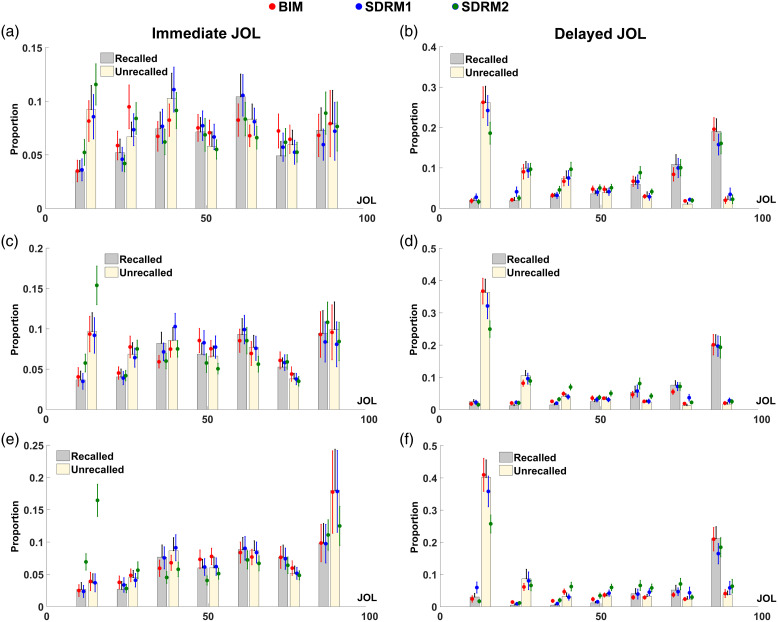
The JOL-Performance Joint Distribution for Immediate JOLs (a for Exp 1, c for Exp 2, and e for Exp 3) and Delayed JOLs (b for Exp 1, d for Exp 2, and f for Exp 3) in Study 2. The Red, Blue, and Green Points Represent the Predictions From BIM, SDRM1, and SDRM2, Respectively. Error Bars Represent Standard Errors *Note*. See the online article for the color version of this figure.

Finally, we compared the fit of BIM and the two SDRM models (i.e., SDRM1 and SDRM2; see Study 1) to the data (see Figure 12). Although all of the three models could fit the overall pattern of JOL-performance distribution for immediate and delayed JOLs, the fit of SDRM2 was imperfect for trials with low JOLs. To further compare the three models, we computed the CV log-likelihood as in Study 1. The aggregated CV log-likelihood was higher for BIM (Exp 1: −3301.81; Exp 2: −3790.37; Exp 3: −2672.99) than SDRM1 (Exp 1: −8245.21; Exp 2: −9904.55; Exp 3: −7792.53) and SDRM2 (Exp 1: −5184.91; Exp 2: −6858.77; Exp 3: −5162.92) in all of the experiments, suggesting that BIM could better predict the data.[Fig fig12]

#### Discussion

The results from BIM revealed that participants relied more on processing experience in delayed than immediate JOLs, which is consistent with our hypothesis based on previous studies (Rhodes & Tauber, 2011; Spellman & Bjork, 1992). Furthermore, the proportion for the contribution of processing experience to delayed JOLs was significantly higher than .5 in all of the three experiments (*t*_s_ > 4.4, *p*_s_ < .001), suggesting that delayed JOLs were mainly based on processing experience (Spellman & Bjork, 1992). We also found that the processing experience utilized in delayed JOLs could more accurately predict memory performance than that in immediate JOLs, supporting a previous theory that immediate JOLs are based on short-term memory while delayed JOLs rely on long-term memory (Nelson & Dunlosky, 1991). In addition, as in Study 1, while both BIM and SDRM could fit the overall pattern of JOLs and performance, BIM could better predict the data according to the CV log-likelihood, possibly because BIM is a more parsimonious model.

### Study 3: Stronger Test of Assumptions in BIM

The core assumption of BIM is that people evaluate their memory performance through a Bayesian inference, in which they integrate the current processing experience and prior beliefs about memory. In Studies 1 and 2, we found that BIM could accurately fit the overall pattern of confidence distribution. However, the previous two studies did not provide direct support for the assumption that participants apply Bayesian inference to evaluate their memory performance. Thus, in Study 3, we conducted a stronger test of the assumption about the Bayesian inference process in BIM. Specifically, we obtained separate estimates for the strength of processing experience and prior beliefs about memory performance from separate data sets, and made parameter-free predictions of the JOL value in each trial via Bayesian inference given people’s prior beliefs about memory and the processing experience during learning. Then we examined whether the true JOL values could be predicted by the computed JOLs based on Bayesian inference. If people give JOLs through a Bayesian inference process in which processing experience and prior beliefs are integrated, then the computed JOLs based on Bayesian inference should significantly predict the true JOL values.

In Study 3, we reanalyzed the data from Experiment 6 of Tauber et al. (2019). In this experiment, participants were asked to learn a list of words. After learning each word, they gave two different JOLs in which they predicted the probability of correctly recalling this word in a later memory test for an average young adult (18–21 years old) and an average old adult (65 + years old), respectively. Before the learning phase, some of the participants also made global predictions about the number of correctly recalled words in the memory test separately for young and old adults. These global predictions should be only based on participants’ prior beliefs because they had not seen the words when giving the global predictions.

We could assume that when participants gave the two JOLs for each word, they should rely on the same (or at least very similar) processing experience because the two JOLs were made for the same word. Thus, we could fit BIM to one experimental condition (e.g., young adult) to estimate the processing experience for each word, and then directly compute the JOLs in the other condition (e.g., old adult) without model fitting by applying Bayesian inference based on the estimated processing experience and participants’ prior beliefs obtained in their global predictions before learning. We examined whether the value of the computed JOL in each trial could significantly predict the true JOL.

#### Participants

There were 85 participants in Experiment 6 of Tauber et al. (2019), in which only 43 participants made belief-based global predictions about memory performance for young and old adults before learning (the other 42 participants gave retrospective global postdictions about memory performance after memory test). Here we only analyzed the data from the participants for whom prior beliefs were measured. One participant was excluded due to using the same JOL value for all trials in each experimental condition, leaving 42 participants in the data analysis.

#### Materials

The materials consisted of 28 neutral English words.

#### Procedure

Participants were asked to learn all of the words and then took a memory test. They gave belief-based global predictions about the number of correctly recalled words in a later memory test separately for young and old adults before the learning phase. After learning each word, they separately gave a JOL representing the recall probability for young and old adults. Participants finished a free recall test after the learning phase, in which they were required to report the learned words as many as possible. See Tauber et al. (2019) for further details of the experimental procedure.

#### Data Analysis

We first examined the difference in mean JOL magnitude and belief-based global predictions between young and old adults. Next, we fit BIM to each experimental condition for each participant and compared the parameters in BIM between the two conditions. Then we compared the fit of BIM and the two SDRM models to the data, as in Studies 1 and 2.

Finally, for each participant, we directly computed the JOL in each trial and investigated whether the value of the computed JOLs could significantly predict the true JOLs. According to the assumption of BIM, people integrate their prior beliefs and processing experience in a Bayesian inference to evaluate their memory performance. Thus, the JOL value can be directly computed through this Bayesian inference given the processing experience and prior beliefs. We assumed that the processing experience utilized in the JOLs for young and old adults in each trial was the same because the two JOLs were made for the same word. Thus, we could estimate the processing experience in each trial by fitting BIM to the JOL data in one experimental condition (e.g., young adult), and then calculate the JOL in each trial for the other condition (e.g., old adult) without model fitting, using this estimated processing experience and participants’ prior beliefs obtained from the belief-based global predictions made before learning (see Section S6 in Supplemental Materials for details of the calculation). We examined the correlation between the computed JOLs and true JOLs to see whether the true value of JOLs could be predicted by our Bayesian inference computation.

#### Results

The mean JOL magnitude was significantly higher for young than old adults, *t*(41) = 3.12, *p* = .003, Cohen’s *d* = .48, η_p_^2^ = .19, suggesting participants predicted that young adults would have higher memory performance than old adults in a later test(see Table 3). Similarly, the belief-based global predictions (converted into beliefs about the proportion of recalled words) were also higher for young (*M* = 50.5%, *SD* = 19.1%) than old adults (*M* = 40.6%, *SD* = 15.3%), *t*(41) = 4.25, *p* < .001, Cohen’s *d* = .66, η_p_^2^ = .31, indicating participants had a prior belief that young adults should have better overall memory ability. We next fit BIM for each participant separately in the young and old adult conditions (see Table 4). Results revealed that the mean of confidence distribution (i.e., parameter *M*_conf_) was significantly higher for young than old adults, *t*(41) = 3.13, *p* = .003, Cohen’s *d* = .48, η_p_^2^ = .19, as in the analysis on the mean JOL magnitude. The other three parameters in BIM (*P*_exp_, μ_*m*_, and ρ) did not significantly differ between young and old adults, *t*_s_ < 1.4, *p*_s_ > .1, Cohen’s *d* < .3, η_p_^2^ < .05.

**Figure 13 fig13:**
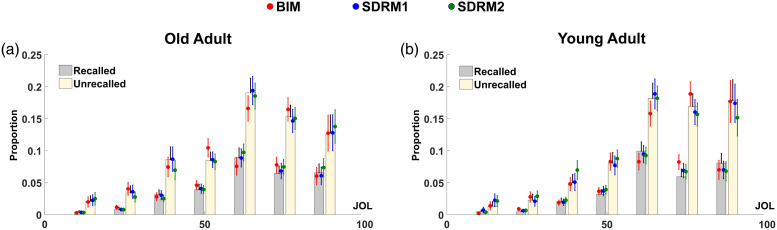
The JOL-Performance Joint Distribution for Old Adult (a) and Young Adult (b) in Study 3. The Red, Blue, and Green Points Represent the Predictions From BIM, SDRM1 and SDRM2, Respectively. Error Bars Represent Standard Errors *Note*. See the online article for the color version of this figure.

We then compared the fit of BIM and the two SDRM models (i.e., SDRM1 and SDRM2; see Study 1) to the data (see Figure 13). All of the three models could fit the overall pattern of JOL-performance distribution for both old and young adults. To further compare the three models, we computed the CV log-likelihood as in Studies 1 and 2. The aggregated CV log-likelihood was higher for BIM (−5334.66) than SDRM1 (−9293.88) and SDRM2 (−7032.19), suggesting that BIM could better predict the data.[Fig fig13]

**Figure 14 fig14:**
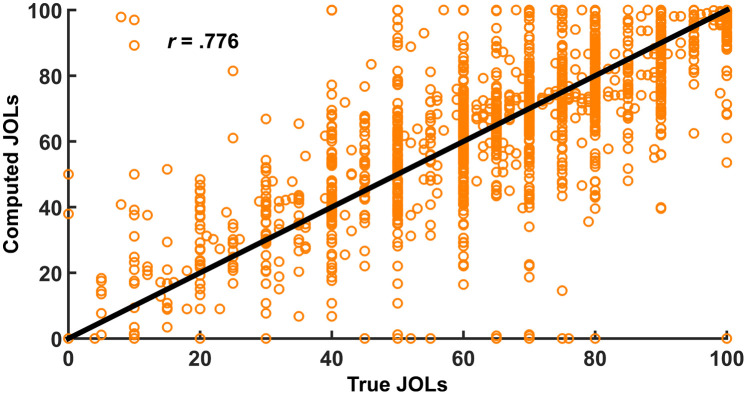
The Relationship Between True and Computed JOLs for All Trials From All of the Participants in Study 3 *Note*. See the online article for the color version of this figure.

Finally, we computed the JOL in each trial by applying Bayesian inference and examined the correlation between the computed and true value of JOLs. We first looked at the overall correlation for all trials from all participants, which showed that the computed JOLs could significantly predict the true JOLs and the correlation was large, *r* = .776, *p* < .001, suggesting the true JOL values could be predicted by our Bayesian inference computation (see Figure 14). We next examined the correlation for each participant. Results indicated that the mean correlation between computed and true JOLs across participants was large (*M* = .635, *SD* = .252), although the prediction for true JOLs was not perfect.[Fig fig14]

One possible concern with this result is that a large correlation between computed and true JOLs may be expected due to an underlying correlation between the two JOLs provided on a given trial. In other words, because the model is basing its prediction on the other JOL from the same trial, a correlation may emerge due to the two JOLs sharing the same word, rather than due to any success of BIM. To rule out this possibility, we built a simple correlation model in which we used the true JOL values in one condition to predict the JOLs in the other condition, and investigated whether the JOLs computed via Bayesian inference could predict the true JOLs better than the simple correlation model. We first examined the overall correlation for all trials from all participants, which showed that the correlation between true JOLs and computed JOLs based on Bayesian inference (*r* = .776) was significantly higher than the correlation between JOLs in two conditions (*r* = .728; *Z* > 5, *p* < .001). We also analyzed the same correlations for each participant, and results again indicated that the mean correlation between true JOLs and computed JOLs based on Bayesian inference (*M* = .635, *SD* = .252) was significantly higher than the mean correlation between JOLs in two conditions (*M* = .446, *SD* = .408), *t*(41) = 3.15, *p* = .003, Cohen’s *d* = .49, η_p_^2^ = .19. These results suggest that the prediction for true JOL values based on Bayesian inference goes beyond that which can be obtained from a simple linear correlation between the JOLs in two experimental conditions.

To further investigate the relationship between the true and computed JOLs, we built linear regression models in which true JOLs were regressed on computed JOLs. If the value of true JOLs could be perfectly predicted by the computed JOLs, then the regression slope should not significantly differ from 1, and the regression intercept should not significantly differ from 0. We first built a regression model for all trials from all participants, and found that the slope was significantly lower than 1, β = .72, *t* = −23.38, *p* < .001, and the intercept was significantly higher than 0, β = 18.92, *t* = 21.86, *p* < .001, suggesting the computed JOLs could not perfectly predict the true JOLs. We next built a regression model for each participant and calculated the slope and intercept. Results also revealed that the overall regression slope for the participant group was significantly lower than 1, *M* = .725, *SD* = .644, *t*(41) = −2.77, *p* = .008, Cohen’s *d* = .43, η_p_^2^ = .16, and the overall intercept was significantly higher than 0, *M* = 18.10, *SD* = 46.86, *t*(41) = 2.50, *p* = .016, Cohen’s *d* = .39, η_p_^2^ = .13, which was consistent with previous analysis. These results further support our conclusion above that although the value of true JOLs could be predicted by our computation via Bayesian inference, this prediction was not perfect.

#### Discussion

As in Studies 1 and 2, we found that BIM could better predict the data than the two SDRM models based on the CV log-likelihood, suggesting that BIM might be a more parsimonious model. More importantly, there was a large correlation between the value of computed and true JOLs, suggesting that the empirical JOLs measured in the experiment could be predicted by our Bayesian inference computation. This result supports the assumption of BIM that people may apply Bayesian inference to evaluate their memory performance. However, we also found that this prediction for the true JOL values was not perfect. There are two possible explanations for this imperfection in the prediction. First, during the learning phase, participants might continue to update their beliefs about memory ability based on the processing experience in each trial (Mueller, Dunlosky, & Tauber, 2014; Rouault et al., 2019). In this study, we used participants’ belief-based global predictions before learning as the prior beliefs when computing the JOL values, which might not fully reflect the evolution of participants’ beliefs during learning (we will further discuss this issue below). Second, the processing experience utilized in the JOLs for young and old adults in the same trial might not be exactly the same. For example, participants might continue accumulating processing experience after giving the first JOL in a trial, leading to a slightly different processing experience when they rated the second JOL for the same word (Navajas et al., 2016).

### Study 4: Example of Fitting BIM to Recognition Memory Data

In this study, we show an example of fitting BIM to empirical data from recognition memory tests with retrospective confidence ratings. We reanalyzed the data from Carpenter et al. (2019) which aimed to improve metacognition through training. In their experiment, Carpenter et al. first asked participants to perform perceptual discrimination and recognition memory tests with retrospective confidence ratings, using words or abstract shapes as stimuli. Then all participants took part in several training sessions across multiple days, in which they performed a perceptual task with confidence ratings. Participants were randomly divided into experimental or control groups: The experimental group received feedback about their metacognitive accuracy during the training sessions, while the control group received feedback about their task performance. After the training sessions, all participants performed the perceptual and memory tasks again with confidence ratings. Results indicated that metacognitive training for the experimental group could improve metacognitive accuracy from pre- to posttraining sessions for not only the trained perceptual task, but also the untrained recognition memory task, suggesting the benefit of metacognition training could transfer to another cognitive domain.

Here we reanalyzed data from the recognition memory task in the pre- and posttraining sessions with either words or shapes used as the stimuli. We fit BIM to the data of memory performance and confidence ratings, and focused on the fitted value of the parameter *P*_exp_. We first examined whether the contribution of processing experience and prior beliefs to confidence was significantly correlated across stimulus types (words or shapes) and sessions. Then we investigated whether metacognitive training in the experimental group influenced the extent to which experience and beliefs affected confidence ratings.

#### Participants

There were 61 participants in Carpenter et al. (2019), including 29 participants in the experimental group and 32 participants in the control group.

#### Procedure

In this study, we focused on the recognition memory tests in Carpenter et al. (2019). In both the pre- and posttraining session, participants completed two recognition memory tests with words or abstract shapes as the to-be-remembered items, respectively. There were 108 trials in the recognition test for each stimulus type in each session. Before the memory test, participants were first presented with a series of stimuli (words or shapes) to memorize. Then in each subsequent trial during the test, a learned and a new stimulus were presented at the same time. Participant were asked to choose the learned stimulus, and then rate confidence in their choice on a 4-point discrete scale. See Carpenter et al. (2019) for further details of the experimental procedure.

#### Results

As in Carpenter et al. (2019), we excluded trials in which either participants did not respond in time (response times >2,000 ms) or response times were less than 200 ms. Then for each participant, we fit BIM to the data from each of the 2 (Stimulus Type: words vs. shapes) × 2 (Session: pre- vs. posttraining) conditions, and focused on the fitted value of the parameter *P*_exp_ in our data analysis (see Table S3 in Supplemental Materials, for the fitted value of the parameter *M*_conf_ in each condition).

We first examined the correlation between *P*_exp_ for different stimulus types (words and shapes). Results showed that for both the experimental and control groups, there was a significant positive correlation between *P*_exp_ for words and shapes in the pretraining session (Experimental Group: *r* = .615, *p* < .001; Control Group: *r* = .492, *p* = .004) and the posttraining session (Experimental Group: *r* = .421, *p* = .023; Control Group: *r* = .754, *p* < .001), suggesting that participants who relied more on processing experience to generate confidence ratings for word stimuli were also more likely to rely on experience for shape stimuli. Next, we explored the correlation between *P*_exp_ in different sessions. We found that in the control group, *P*_exp_ in the pre- and posttraining sessions was significantly correlated for words (*r* = .472, *p* = .006) and shapes (*r* = .513, *p* = .003), indicating that participants who relied more on processing experience to generate their confidence before training tended to also rely more on experience after training. However, in the experimental group, *P*_exp_ in the two sessions did not significantly correlate with each other for either words (*r* = .096, *p* = .619) or shapes (*r* = .055, *p* = .776).

**Figure 15 fig15:**
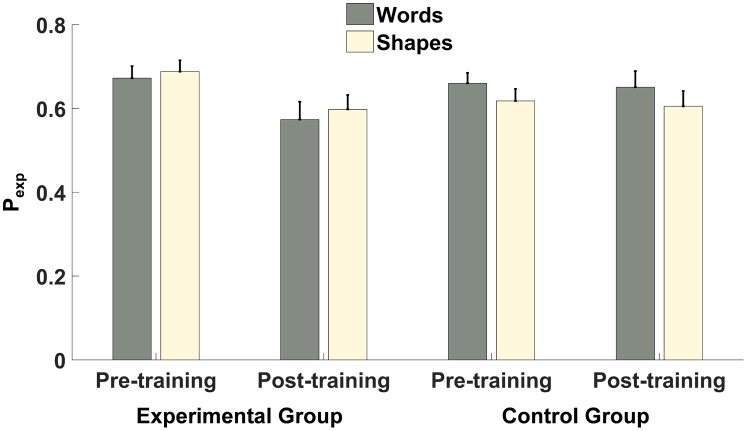
The Fitted Value of the Parameter P*_*exp*_* as a Function of Stimulus Type (Words vs. Shapes), Group (Experimental vs. Control), and Session (Pre- vs. Posttraining) in Study 4. Error Bars Represent Standard Errors *Note*. See the online article for the color version of this figure.

We then conducted a 2 (Session: pre- vs. posttraining) × 2 (Stimulus Type: words vs. shapes) × 2 (Group: experimental vs. control) ANOVA on the fitted value of *P*_exp_ (see Figure 15). There was a marginally significant interaction between Stimulus Type and Group, *F*(1, 59) = 3.92, *p* = .052, η_p_^2^ = .06, indicating that the difference in the contribution of processing experience to confidence between the two stimulus types was larger in the control than the experimental group. In addition, there was a significant main effect of Session, *F*(1, 59) = 4.76, *p* = .033, η_p_^2^ = .08, suggesting that the overall contribution of processing experience to confidence was higher in the pre- compared to the posttraining session. More importantly, the Session × Group interaction was close to significance, *F*(1, 59) = 2.98, *p* = .089, η_p_^2^ = .05. Further analysis revealed that *P*_exp_ did not significantly differ across sessions in the control group, *F*(1, 31) = .15, *p* = .704, η_p_^2^ < .01, but was significantly reduced in the posttraining session compared with the pretraining session for the experimental group, *F*(1, 28) = 5.65, *p* = .025, η_p_^2^ = .17. These results suggest that metacognitive training in the experimental group could reduce the contribution of processing experience to confidence ratings in the recognition memory test, and increase the contribution of prior beliefs.[Fig fig15]

#### Discussion

In this study, we demonstrated an example of fitting BIM to data from recognition tests and analyzing the fitted value of the parameter *P*_exp_ across different experimental conditions. We found that the contribution of processing experience and prior beliefs to confidence ratings for words and shapes significantly correlated with each other, suggesting that the integration of processing experience and prior beliefs via Bayesian inference might generalize across stimulus types. In addition, participants’ reliance on experience and beliefs was stable across time for the control group. However, metacognitive training for the experimental group significantly reduced the contribution of processing experience to confidence in the posttraining session, and might explain why the correlation between the *P*_exp_ in different sessions was only significant for the control group but not for the experimental group. One possible explanation for the reduction of *P*_exp_ following metacognitive training is that participants received feedback about their metacognitive performance during training, and were able to develop and adjust their metacognitive beliefs about their performance based on this feedback. Although these beliefs were developed in a perception task, participants might transfer their beliefs to the memory test and use these beliefs to generate confidence ratings during the posttraining session.

## Extension of BIM: Belief Updating

The basic assumption of BIM is that people evaluate their memory performance by integrating current processing experience and prior beliefs about memory ability via Bayesian inference. Up until now, we have assumed that people’s prior beliefs about memory are developed before the memory task and do not change during learning and test. However, this may be an oversimplification because previous empirical studies have shown that people are able to (at least partly) update their beliefs about memory ability during the memory and metamemory process (Hertzog et al., 2009; Mueller, Dunlosky, & Tauber, 2014). Here we provide an extension of BIM which may explain this updating of beliefs during the metamemory process. In this extended BIM, we assume that when people rate their confidence for each trial via Bayesian inference, they also continuously update their beliefs about memory ability by applying a second Bayesian inference that incorporates the processing experience in each trial. We also performed a data simulation to quantitatively illustrate this belief updating process across trials, and discuss how we may detect the belief updating in empirical data.

### Model Details

To illustrate the belief updating process in the extended BIM, suppose that the prior belief distribution, which represents participants’ prior belief about memory ability developed before the memory task, has a mean of μ_*b*_ and a standard deviation of 1 (see Figure 1). Then in the first trial of the memory task, participants take a sample of processing experience, which is denoted by *e*_1_. According to the assumptions of BIM, participants rate their confidence for the first trial through a Bayesian inference, in which they infer the posterior distribution of the subjective memory strength $\hat{m}$ (see also Equation 1):

$$f_{c}(\hat{m}|e_{1})\propto f(\hat{m})f_{c}(e_{1}|\hat{m})$$ 20

In Equation (20), *f*_*c*_ ($\hat{m}$ | *e*_1_) represents the posterior distribution for $\hat{m}$, which is used to generate the confidence rating in the first trial. In a recall task, the confidence predicted by BIM is the posterior probability that $\hat{m}$ is higher than 0, i.e., *P*_c_ ($\hat{m}$ > 0 | *e*_1_). In a recognition task, the predicted confidence is the posterior probability that $\hat{m}$ is higher or lower than the Type I criterion *C* depending on the Type I response (S1 or S2), i.e., *P*_c_ ($\hat{m}$ > *C* | *e*_1_) or *P*_*c*_ ($\hat{m}$ < *C* | *e*_1_). The posterior distribution *f*_*c*_ ($\hat{m}$ | *e*_1_) is inferred based on (a) the prior belief distribution *f* ($\hat{m}$), which has a mean of μ_*b*_ and a standard deviation of 1, and (b) the likelihood function *f*_*c*_ (*e*_1_ | $\hat{m}$), which is a normal distribution with a mean of $\hat{m}$ and a standard deviation of σ_*l*_ (see Figure 1). σ_*l*_ reflects participants’ knowledge about the relationship between memory strength $\hat{m}$ and processing experience *e*, and participants rely more on processing experience to generate their confidence rating when σ_*l*_ is lower (i.e., when *P*_exp_ is higher; see Equation 4).

In the extended BIM, we assume that at the same time participants also update their beliefs about memory using another Bayesian inference when rating their confidence:

$$f_{b}(\hat{m}|e_{1})\propto f(\hat{m})f_{b}(e_{1}|\hat{m})$$ 21

Here, we use *f*_*b*_ ($\hat{m}$ | *e*_1_) to represent the updated distribution of beliefs about memory strength $\hat{m}$ based on the processing experience *e*_*1*_ in the first trial. This Bayesian inference for belief updating is very similar to the previous Bayesian inference generating confidence ratings. The prior belief distribution *f* ($\hat{m}$) utilized in this inference is the same as that in Equation 20. The likelihood function *f*_*b*_ (*e*_1_ | $\hat{m}$) is also very similar to that in Equation 20 (i.e., *f*_*c*_ (*e*_1_ | $\hat{m}$)) except that the standard deviation of the likelihood function (denoted by σ_*lb*_) is different from the σ_*l*_ used previously. Typically, the value of σ_*lb*_ should be larger than the value of σ_*l*_, as we expect updated beliefs to mainly rely on prior beliefs rather than current processing experience. This is because previous studies indicate that belief updating based on processing experience is often slow and incomplete (Hertzog et al., 2009; Mueller, Dunlosky, Tauber, 2014).

Then, in the second trial, participants take another sample of processing experience denoted by *e*_2_. To rate their confidence for the second trial, they apply a Bayesian inference in which they use beliefs about memory updated in the first trial:

$$f_{c}(\hat{m}|e_{2})\propto f_{b}(\hat{m}|e_{1})f_{c}(e_{2}|\hat{m})$$ 22

In Equation 22, *f*_*c*_ ($\hat{m}$ | *e*_2_) refers to the posterior distribution of $\hat{m}$ used for the confidence rating in the second trial. *f*_*b*_ ($\hat{m}$ | *e*_1_) represents participants’ beliefs about memory strength $\hat{m}$, which was updated in the first trial. The likelihood function *f*_*c*_ (*e*_2_ | $\hat{m}$) is the same as that in the first trial and has a standard deviation of σ_*l*_.

In the second trial, participants also update their beliefs about memory based on processing experience *e*_2_:

$$f_{b}(\hat{m}|e_{1},e_{2})\propto f_{b}(\hat{m}|e_{1})f_{b}(e_{2}|\hat{m})$$ 23

In Equation 23, *f*_*b*_ ($\hat{m}$ | *e*_1_) represents the beliefs updated in the first trial, and *f*_*b*_ ($\hat{m}$ | *e*_1_, *e*_2_) refers to the beliefs updated in the second trial. The likelihood function *f*_*b*_ (*e*_2_ | $\hat{m}$) is the same as that in the first trial and has a standard deviation of σ_*l*b_.

Similarly, when participants rate their confidence for the *i*th trial, they conduct a Bayesian inference using the beliefs updated in the (*i* − 1)th trial:

$$f_{c}(\hat{m}|e_{i})\propto f_{b}(\hat{m}|e_{1},e_{2},\cdots e_{i-1})f_{c}(e_{i}|\hat{m})$$ 24

At the same time, they update their beliefs about memory based on the processing experience in the *i*th trial:

$$f_{b}(\hat{m}|e_{1},e_{2},\cdots e_{i-1},e_{i})\propto f_{b}(\hat{m}|e_{1},e_{2},\cdots e_{i-1})f_{b}(e_{i}|\hat{m})$$ 25

BIM assumes that this belief updating process should be similar for the recall and recognition tasks. The only difference between the two tasks is that in the recognition task, participants’ subjective beliefs about the distribution of memory strength $\hat{m}$ may be different for S1 and S2 stimulus. Thus, participants should only update one of the two belief distributions in each trial (either for S1 or S2), which depends on the Type I response given by participants.

### Data Simulation

In this section, we simulated data from a hypothetical recall task with confidence ratings to quantitatively illustrate the belief updating process. In the simulation, we constructed the distribution of processing experience with a standard deviation of 1 (as assumed by BIM) and a mean (μ_*e*_) of.5. In addition, the distribution for the prior beliefs developed before the task was set with a mean (μ_*b*_) of 0 and a standard deviation (σ_*b*_) of 1. We also set the standard deviation of the likelihood function in the Bayesian inference to 1 for confidence ratings (i.e., σ_*l*_ = 1) and either 10, 15, or 20 for belief updating (i.e., σ_*lb*_ = 10, 15 or 20). Then for each parameter set, we simulated data from 100 participants with 100 trials per participant to obtain stable results for the belief updating process. In each trial, we took a sample from the processing experience distribution as the processing experience for the current trial and computed the confidence rating on a continuous 0–1 scale in this trial based on the equations of the extended BIM.

**Figure 16 fig16:**
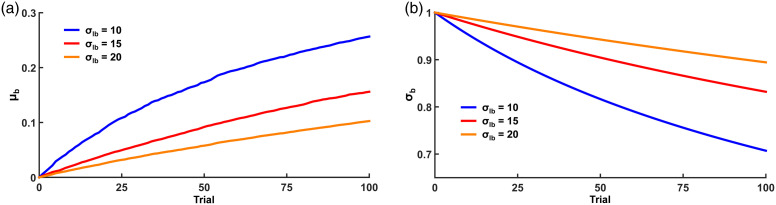
The (a) Mean (μ*_*b*_*) and (b) Standard Deviation (σ*_*b*_*) of the Belief Distribution Across Trials in the Data Simulated From the Extended BIM. (Averaged for All Simulated Participants) *Note*. See the online article for the color version of this figure.

Figure 16 shows the change of the mean and standard deviation for the belief distribution (i.e., μ_*b*_ and σ_b_) across trials (averaged for all simulated participants). We see that μ_*b*_ gradually increased across trials. This is because the overall strength of processing experience (reflected by the mean of processing experience distribution, μ_*e*_, which was .5) was stronger than participants’ prior beliefs about memory developed before the memory task (reflected by the original μ_*b*_ before the task, which was 0). In this example, participants update their beliefs about memory ability based on their processing experience in each trial and end up believing that they have higher overall memory performance. In addition, σ_b_ decreased across trials, suggesting that participants were more certain about their beliefs after belief updating. We should also note that the belief updating process was faster when the standard deviation of the likelihood function for belief updating (σ_*l*b_) was smaller because participants relied more on the current processing experience rather than prior beliefs when σ_*l*b_ was smaller.[Fig fig16]

**Figure 17 fig17:**
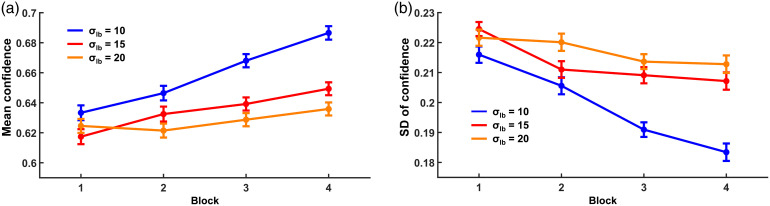
The (a) Mean and (b) Standard Deviation of Confidence Ratings Across Blocks in the Data Simulated From the Extended BIM. Error Bars Represent Standard Errors *Note*. See the online article for the color version of this figure.

Next, we looked at the change of confidence ratings across trials during the belief updating process. For each participant, we divided the 100 trials into 4 blocks based on the trial order, with each block containing 25 trials. Then we calculated the mean and standard deviation of the confidence ratings separately for each block (see Figure 17). Results revealed a trend that the mean confidence increased across blocks because during belief updating participants developed new beliefs that they should have better memory ability. In addition, the standard deviation of confidence tended to decrease across blocks. This is because the uncertainty in beliefs about memory reduced during belief updating, and the confidence ratings should be distributed more closely around the updated beliefs in later blocks. In brief, we could conclude that the belief updating process affected the distribution of confidence ratings across blocks, changing its mean, and reducing the variability.[Fig fig17]

**Figure 18 fig18:**
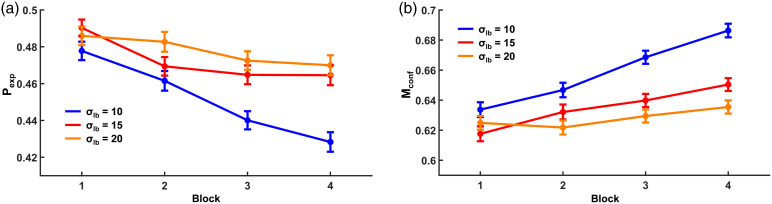
The Estimated Value of (a) P*_*exp*_* and (b) M*_*conf*_* Across Blocks in the Restricted BIM Fitted to the Data Simulated From the Extended BIM. Error Bars Represent Standard Errors *Note*. See the online article for the color version of this figure.

The extended BIM cannot be directly fitted to empirical confidence ratings because it allows variation in the uncertainty of beliefs about memory across trials, which introduces redundant free parameters and makes the model nonidentifiable. However, we can fit the restricted version of BIM, in which belief updating is not allowed (i.e., the BIM we have used in previous sections), to confidence data simulated from the extended BIM. Figure 18 shows the fitted value of *P*_exp_ and *M*_conf_ in the restricted BIM for each block.^12^ We could see that the fitted *P*_exp_ reduced across blocks, suggesting the fitted value of σ_*l*_ increased during belief updating (see Equation 4). However, the true value of σ_*l*_ in the extended BIM was set as a constant (σ_*l*_ = 1) in our data simulation. This increase of fitted σ_*l*_ in the restricted BIM is due to the fact that the decreasing of belief uncertainty in the extended BIM during belief updating reduced the variance of confidence ratings, which was mimicked by the effect of increasing σ_*l*_ (or decreasing *P*_exp_) in the restricted BIM (see Figure 4). In addition, the fitted *M*_conf_ in the restricted BIM increased across blocks. *M*_conf_ is based on the mean of the distributions for both the processing experience (μ_*e*_) and beliefs (μ_*b*_), and should increase when participants develop higher beliefs about their memory ability.[Fig fig18]

In summary, we are not able to directly fit the extended BIM to empirical confidence data to examine whether participants update their beliefs about memory ability across trials based on processing experience. However, we can fit the restricted BIM to data and use the following results as potential evidence for belief updating across trials: (a) the fitted *P*_exp_ tends to decrease across trials and (b) the fitted *M*_conf_ changes approximately monotonically toward a new value. In addition, the change of fitted *P*_exp_ and *M*_conf_ across trials may be subtle and noisy with only a few participants and trials, and we recommend future studies use a relatively large number of participants and trials to examine these effects.

## General Discussion

Although previous studies have shown that both processing experience and prior beliefs can significantly contribute to metamemory monitoring (e.g., Hu et al., 2015; Mueller, Dunlosky, Tauber, et al., 2014; Mueller et al., 2013, 2016; Undorf & Erdfelder, 2015; Yang, Huang, et al., 2018), few have proposed formal computational models to explain how people combine processing experience and prior beliefs to evaluate their memory performance. To address this question, in the current study we introduce BIM which assumes that during the metamemory process people integrate processing experience and prior beliefs via Bayesian inference. Our results from data simulation revealed successful recovery for most of the parameters in BIM, and also indicated a significant relationship between parameters in BIM and other computational models such as SDRM and the meta-*d*′ model (Jang et al., 2012; Maniscalco & Lau, 2012). We then fit BIM to empirical data sets in four studies, which suggest that BIM could make predictions consistent with previous theories of metamemory, and provide a better prediction of the data in recall tasks than SDRM (as revealed by CV log-likelihood). In addition, BIM could be fitted to empirical confidence ratings not only from recall tasks but also recognition tasks. Finally, we discuss an extension of BIM which may explain how people update their beliefs about memory based on the processing experience.

BIM suggests that prior beliefs act as an anchor in metamemory judgments (e.g., confidence ratings). As shown in Figures 4 and 7, the variance of the confidence distribution is higher when the contribution of processing experience to metamemory (i.e., *P*_exp_) is higher. This is because BIM assumes when *P*_exp_ is small, confidence ratings are largely affected by people’s prior beliefs about their overall memory ability, which are developed prior to the memory process. In contrast, confidence ratings closely track the variation of processing experience across trials when *P*_exp_ is high, which in turn increases the variance of the confidence distribution. These features are consistent with the anchoring hypothesis of metamemory, which indicates that during metamemory monitoring people set an anchor that reflects their beliefs about memory performance, and then adjust their confidence ratings across trials around the anchor (Scheck et al., 2004; Yang, Sun, et al., 2018). In addition, the anchoring hypothesis predicts that delayed JOLs are less affected by the anchor than immediate JOLs (Scheck et al., 2004). This is consistent with the results in our Study 2 showing that processing experience contributed more to delayed than immediate JOLs.

Our results from the simulations of confidence distributions also suggest that the correlation between processing experience and actual memory strength (i.e., the parameter ρ in BIM) affects the confidence distribution differently in recall and recognition tasks (see Figures 5 and 7). In recall tasks, increasing ρ can increase the confidence of recalled trials and decrease the confidence of unrecalled trials. However, in recognition tasks, increasing ρ leads to higher confidence ratings for both the correct and incorrect trials. The reason for this difference is that the Type I response affects the subsequent confidence rating process in recognition tasks but not recall tasks. During the metamemory process in recall tasks, people only need to evaluate the probability that each item is correctly recalled, or the probability that memory strength is higher than a recall criterion, based on the processing experience. When the correlation between processing experience and actual memory strength is high, recalled trials should result in higher processing experience than unrecalled trials, leading to high confidence for recalled trials but low confidence for unrecalled trials. In contrast, in recognition tasks, people’s confidence rating depends on whether they respond S1 or S2 in Type I task. They need to evaluate the probability that the estimated memory strength $\hat{m}$ is lower than the Type I criterion *C* given the processing experience *e* when the Type I response is S1, and the probability that $\hat{m}$ is higher than *C* given *e* when the Type I response is S2. In recognition tasks, people give an S1 response when the objective memory strength *m* is lower than the Type I criterion *C*, regardless of whether the true stimulus is S1 or S2. When ρ is high, the processing experience *e* is also more likely to be lower than *C* following S1 responses, and higher than *C* following S2 responses, increasing people’s confidence that a Type I response is correct. Thus, increasing ρ in recognition tasks can lead to higher confidence not only for correct trials but also for incorrect trials. This is consistent with the second-order model for metacognition proposed by Fleming and Daw (2017), which suggests that increasing the correlation between the variables supporting Type I decisions and confidence ratings may reduce metacognitive accuracy due to a lower possibility of detecting errors in Type I responses.

Our parameter recovery analysis indicates that when BIM is fitted to recall tasks with either continuous or discrete confidence ratings, all of the parameters in BIM (including *P*_exp_, *M*_conf_, μ_*m*_, and ρ) can be successfully recovered with only a few trials (e.g., 10–30 trials) for each participant. One possible explanation for this good recovery in recall tasks is that BIM is a relatively parsimonious model. In contrast to previous computational models (such as SDRM and the meta-*d*′ model) in which each confidence criterion is a free parameter (Jang et al., 2012; Maniscalco & Lau, 2012), BIM for recall tasks only uses two parameters (*P*_exp_ and *M*_conf_) to characterize the shape of the overall confidence distribution and may be able to offer more stable parameter estimates. When BIM is fitted to recognition tasks with retrospective confidence ratings, we also need only a few trials to recover *P*_exp_ and four *M*_conf_ parameters in 2 (stimulus: S1 vs. S2) × 2 (response: S1 vs. S2) conditions if confidence is rated on a 0–1 continuous scale. However, in most of the previous studies on retrospective confidence ratings, participants were asked to rate their confidence on a discrete scale (Rahnev et al., 2020). When we fit BIM to confidence data on a discrete scale in recognition tasks, only a few trials (e.g., 10–20 trials) are needed if we are only interested in the fitted value of *P*_exp_ (as in our Study 4). However, we suggest future studies should include a relatively large number of trials (e.g., more than 90 trials) if they aim to estimate all four *M*_conf_ parameters from discrete confidence data in recognition tasks. Furthermore, we do not recommend analyzing the fitted value of ρ in recognition tasks because the estimation of ρ is often inaccurate.

When examining the relationship between parameters in BIM and other computational models such as SDRM and the meta-*d*′ model, we found that the parameter μ_*m*_ in BIM for recall tasks was closely related to the parameter reflecting memory performance in SDRM, and ρ in BIM for recall tasks was significantly correlated with different indices of metamemory accuracy. More interestingly, the parameters *P*_exp_ and *M*_conf_ in BIM for either recall or recognition tasks were separately correlated with the variance and mean of confidence criteria in other models. These results are related to the computational model proposed by Selker et al. (2019) which characterizes a set of confidence criteria with only a location parameter and a scale parameter. In BIM, *M*_conf_ can be seen as a location parameter and *P*_exp_ as a scale parameter for confidence criteria. According to BIM, the scale (or variance) of confidence criteria is based on the relative contribution of processing experience and prior beliefs to confidence ratings, and the location (or mean) of confidence criteria is related to the integrated effect of experience and beliefs on confidence.

We then showed how to fit BIM to empirical data in four studies. Our Studies 1 and 2 indicated that results from BIM about how much processing experience and prior beliefs contributed to JOLs were consistent with the theories based on previous studies. For example, we found that the contribution of processing experience to memory predictions (i.e., *P*_exp_) was significantly higher for post- than prestudy JOLs, and for delayed than immediate JOLs. Previous theories of metamemory indicate that people largely rely on their prior beliefs about overall memory ability to guess memory performance when giving prestudy JOLs, and use processing experience from retrieval attempts to infer their memory strength during metamemory monitoring after a delay (Mueller, Dunlosky, Tauber, et al., 2014; Spellman & Bjork, 1992). The results from BIM are consistent with these theories, suggesting that BIM is theoretically reasonable. In Study 3, we examined whether observed JOLs could be predicted by our Bayesian inference computation given the processing experience in each trial and participants’ prior beliefs about memory. Our results showed a substantial correlation between computed and true JOLs, providing further evidence that metamemory monitoring may be based on a Bayesian inference process in which processing experience and prior beliefs are integrated. In addition, in all of the first three studies, we found that BIM predicted the data better than SDRM (as revealed by CV log-likelihood), possibly because BIM is a more parsimonious model. In Study 4, we provide an example of fitting BIM to recognition tasks with retrospective confidence ratings, which revealed that the Bayesian inference process during metamemory might generalize across different study materials, and that metacognitive training might increase reliance on prior beliefs.

Finally, we introduce an extended version of BIM which may account for the updating of beliefs about memory based on the processing experience during previous episodes of metamemory monitoring. Although we are not able to directly fit the extended BIM to confidence data to examine the updating of beliefs across trials because this model is nonidentifiable, the evidence for belief updating may be still reflected in a change in fitted parameters across trials in the restricted BIM. First, a reduction of belief uncertainty (i.e., the standard deviation of belief distribution, σ_b_) during belief updating could reduce the variance of the observed confidence distribution, and this effect could be mimicked by a decrease in fitted *P*_exp_ (or an increase of the fitted standard deviation of likelihood function, σ_*l*_) in the restricted BIM. Second, the fitted *M*_conf_ in the restricted BIM should gradually change toward a new value based on the updated beliefs. Future studies should use a relatively large number of trials and participants to test these hypotheses in empirical data.

We note that the effect of a change in σ_b_ on confidence distribution can be mimicked by the effect of a change in σ_*l*_ because both parameters can affect the variability of confidence ratings (see also Section S3 in Supplemental Materials). However, σ_b_ in the restricted BIM is fixed to 1 in order to make the model identifiable, and the restricted BIM can only attribute a change in the variability of confidence to a change in σ_*l*_. Thus, when we fit the restricted BIM to confidence data in two experimental conditions and observe that the contribution of processing experience to confidence (i.e., fitted value of *P*_exp_) is higher in Condition 1 than Condition 2, there are two possibilities for this difference: (a) participants in Condition 1 assume there is a more close relationship between processing experience and memory strength (i.e., σ_*l*_ in Condition 1 is lower than Condition 2), or (b) participants in Condition 1 are actually more uncertain of their beliefs about their overall memory ability (i.e., σ_b_ in Condition 1 is higher than Condition 2). The restricted BIM is not able to discriminate the two possibilities based on the fitted parameters, which is an important limitation of BIM.

In some experimental designs, we may assume that uncertainty in beliefs about memory is approximately equal across conditions. For example, in Studies 1 and 2, we asked participants to make either a prestudy JOL, immediate poststudy JOL or delayed poststudy JOL for each trial. These different types of JOLs should rely on the same beliefs about participants’ overall memory performance in a later memory test, and belief uncertainty should be the same across JOL types. In addition, participants in many previous studies were asked to give confidence ratings about their memory performance for different levels of a manipulated variable related to study materials, such as for word pairs with high or low semantic relatedness between two words, or stimuli presented in either large or small font (Hu et al., 2015; Mueller, Dunlosky, Tauber, et al., 2014; Mueller et al., 2013; Su et al., 2018). We may assume that the uncertainty in participants’ beliefs about memory performance is approximately the same across different levels of the variable. In these cases, the difference in fitted *P*_exp_ across conditions should be attributed to the difference in σ_*l*_, which reflects participants’ assumptions about the relationship between processing experience and memory strength. However, belief uncertainty may be different in other experimental designs. For example, when participants are asked to make belief-based predictions about memory performance separately for an immediate test and a test after 1 week, they may be more certain about their performance in the near than distant future. Furthermore, in our Study 4 we found that the fitted *P*_exp_ for confidence in recognition tasks was reduced after metacognitive training. One possible explanation is that participants developed and updated new metacognitive beliefs during training, and thus their belief uncertainty was reduced in the posttraining session. In summary, we need to be cautious when explaining why there is a difference in the contribution of processing experience and beliefs to confidence across conditions.

To our knowledge, BIM is the first computational framework to systematically account for how people combine current processing experience and prior beliefs about memory ability to evaluate their memory performance. Previous empirical studies on this topic are often grounded in the dual-basis theory for metamemory, which only qualitatively proposes that metamemory monitoring is affected by processing experience and prior beliefs, and does not quantitatively account for how people combine these two sources of information during the confidence rating process (Koriat, 1997; Koriat et al., 2004). In contrast, BIM seeks to identify psychologically meaningful latent parameters that characterize this integration of processing experience and prior beliefs using a Bayesian inference process, and our Studies 1–2 suggest that the predictions made by BIM were consistent with previous studies based on the dual-basis theory (Mueller, Dunlosky, Tauber, et al., 2014; Spellman & Bjork, 1992). Future studies should further examine BIM’s predictions about metamemory judgments in different tasks, and use BIM to investigate whether processing experience and prior beliefs differently affect metamemory judgments when participants utilize different cues (such as the characteristics of study materials or those of learning process) in the metamemory process (Koriat, 1997).

Another contribution of BIM to metamemory research is that BIM tries to explain why the usage of confidence scale varies across individuals and experimental conditions. Many empirical studies on metamemory judgments indicate that participants use the confidence scale differently in different experimental conditions: They prefer to use the middle of the scale in some conditions and the end of the scale in the other (e.g., Dunlosky & Nelson, 1994; Nelson et al., 2004; Weaver & Kelemen, 1997). Previous computational models for metamemory (such as SDRM and the meta-*d*′ model) attribute the different usage of the confidence scale to the setting of confidence criteria, and suggest that participants may show preferences for different parts of the confidence scale across conditions. These models include one free parameter for each confidence criteria, and do not focus on the psychological interpretation of the different setting of criteria across conditions (Jang et al., 2012; Maniscalco & Lau, 2012). In contrast, BIM attributes different usages of the confidence scale to differential reliance on processing experience and prior beliefs about memory ability.

BIM assumes that people use the confidence scale in an accurate and linear manner: Reported confidence and the predicted confidence generated by BIM have a tight linear relationship when confidence ratings are reported on a 0–1 *continuous scale*, and the 0–1 confidence scale is divided into equal-length bins when confidence ratings are reported on an *n*-point discrete scale. In BIM, unlike in previous models, the different usages of the confidence scale observed in empirical data are directly attributed to different contributions of processing experience and prior beliefs in the Bayesian inference process: People tend to use the middle of confidence scale when they mainly rely on their prior beliefs about memory to rate their confidence, and the ends of the scale when processing experience contributes more to confidence ratings (see Figures 4 and 7). In addition, our Study 3 showed that the true value of JOLs could be significantly predicted by our computation via Bayesian inference (although this prediction was not perfect), while previous computational models for metamemory are not able to make such predictions for JOL values. Thus, BIM gives greater psychological depth and interpretation to how confidence scale usage changes across conditions and individuals, which in turn may motivate future studies to further investigate how different factors interact to affect the confidence distributions. For example, future studies may examine whether the usage of the confidence scale is affected by certain processing experience during memory tasks, or by the activation of different beliefs about memory performance.

In our Studies 1–4, participants rated their confidence ratings on either a numerical continuous scale (0%–100% percentage scale) or discrete scale with number labels (a 4-point scale labeled from 1 to 4). BIM assumes that when rating confidence on a numerical continuous or discrete scale, people naturally use the scale in a linear manner. We need to be more cautious when applying BIM to confidence data on a verbal-labeled discrete scale (e.g., a 4-point scale using *not confident at all*, *low confidence*, *high confidence*, and *complete confidence* without corresponding numerical labels) because different participants may link different levels of posterior probability on the 0–1 *continuous confidence scale* to *each verbal label*.

In contrast to previous computational models of metamemory, BIM not only characterizes the accuracy of confidence ratings (e.g., the parameter ρ in BIM for recall tasks), but also includes a parameter representing how much our confidence is based on processing experience or prior beliefs (the parameter *P*_exp_). Although *P*_exp_ does not correlate with metamemory accuracy, it significantly affects how we evaluate our memory performance and thus the distribution of confidence ratings. Future studies might investigate whether experience and beliefs affect confidence ratings similarly or differently across different types of memory such as episodic and semantic memory tasks (Mazancieux et al., 2020), and neural correlates of individual differences in the contribution of experience and beliefs. Theoretically, BIM can also be fitted to data in any cognitive task with confidence ratings such as perceptual tasks (Fleming et al., 2010). For instance, people may have prior beliefs or expectations about the overall difficulty of a perceptual task, which can affect their confidence ratings. Future studies could compare the fit of BIM and other computational models to data from cognitive domains other than memory, and examine whether the contribution of experience and beliefs to confidence is correlated across different cognitive domains.

Previous studies have used another statistical method, multilevel mediation, to examine the relative contribution of processing experience and prior beliefs to metamemory (Hu et al., 2020; Su et al., 2018; Undorf & Erdfelder, 2015; Undorf et al., 2017; Yang, Huang, et al., 2018). For example, Yang, Huang, et al. (2018) measured perceptual fluency for items presented in large or small font size as the processing experience, and built a multilevel mediation model to investigate how much perceptual fluency could explain the font-size effect on JOLs. Their results revealed that perceptual fluency mediated 20% of the font-size effect. We note that there are important differences between BIM and the multilevel mediation approach. First, the multilevel mediation model focuses on whether the difference in processing experience or prior beliefs between experimental conditions (e.g., large vs. small font) can explain the difference in confidence ratings between conditions, while BIM estimates the absolute contribution of processing experience and beliefs to confidence. For example, suppose that the processing experience in Conditions 1 and 2 is the same while the prior beliefs are different. The parameter *P*_exp_ in BIM is .5 for both conditions, indicating that people rely on processing experience and prior beliefs to the same extent when giving confidence ratings. However, a multilevel mediation model should reveal that processing experience mediates 0% (rather than 50%) of the effect of experimental conditions on confidence because processing experience in both conditions is the same and the difference in confidence is entirely due to the difference in prior beliefs. Another difference between BIM and the multilevel mediation model is that BIM can estimate the contribution of processing experience and prior beliefs to confidence without directly measuring either experience or beliefs, which is impossible for multilevel mediation approaches. Thus, we should choose the appropriate model to examine the contribution of experience and beliefs to confidence according to our theoretical hypothesis and experimental design.

The core assumption of BIM is that during metamemory monitoring, people integrate processing experience and prior beliefs via Bayesian inference, in which the relative contribution of processing experience and prior beliefs (i.e., *P*_exp_) is controlled by the standard deviation of the likelihood function (σ_*l*_). σ_*l*_ reflects people’s assumption about the relationship between processing experience and objective memory. Processing experience contributes more (and prior beliefs contribute less) to metamemory monitoring when people have an assumption that processing experience is more closely distributed around the memory strength (i.e., when σ_*l*_ is smaller). However, BIM is not able to tell whether this assumption affects metamemory monitoring in an explicit or implicit manner. One possibility is that people may deliberately infer the relationship between processing experience and memory strength, based on which they explicitly decide how to utilize processing experience and prior beliefs to evaluate their memory performance (Mueller & Dunlosky, 2017). It is also possible that certain processing experiences (e.g., high perceptual fluency during the identification of study materials) trigger a subjective feeling that the materials are easy to remember, which then implicitly leads people to predict that they should have high performance in the memory test (Oppenheimer, 2008). Future studies should try to distinguish these two possibilities.

Another limitation of BIM needs to be mentioned. Although BIM can estimate how much processing experience contributes to metamemory monitoring, it cannot tell which kind of processing experience affects metamemory. Future studies should measure processing experience in different ways (e.g., using response time or self-paced study time; see Mueller, Dunlosky, Tauber, et al., 2014; Yang, Huang, et al., 2018) and compare the measured processing experience and estimated parameters from BIM.

## Supplementary Material

10.1037/rev0000270.supp

## Figures and Tables

**Table 1 tbl1:** Spearman Correlation Between Parameters in BIM for Recall Tasks and Other Metamemory Metrics/Models

Metrics	*P*_exp_	*M*_conf_	μ_*m*_	ρ
Gamma	−.056	.059	−.031	.991^a^
AUROC	−.046	.062	−.016	.969^a^
**SDRM**				
ρ	−.048	.053	−.020	.993^a^
*M*_Cconf_	.025	−.885^a^	.021	−.029
*SD*_Cconf_	−.937^a^	−.037	−.005	.031
*C*_M_	−.007	.033	−.997^a^	.029
*Note*. ^a^ The absolute value of the correlation coefficient is higher than .5.				

**Table 2 tbl2:** Spearman Correlation Between Parameters in BIM for Recognition Tasks and Other Metamemory Metrics/Models

Metrics	*P*_exp_	*M*_conf (sS1, rS1)_	*M*_conf (sS1, rS2)_	*M*_conf (sS2, rS1)_	*M*_conf (sS2, rS2)_
Gamma	−.001	.369	−.381	−.387	.404
AUROC	−.004	.376	−.382	−.385	.411
**Meta-** ***d*** **′**					
meta-*d*′	−.023	.360	−.370	−.346	.380
*M*_CrS1_	.223	.311	.007	.382	−.013
*SD*_CrS1_	−.690^a^	−.237	−.037	−.249	−.011
*M*_CrS2_	−.186	.012	−.337	.066	−.350
*SD*_CrS2_	−.720^a^	−.016	−.304	−.005	−.208
*Note*. ^a^ The absolute value of the correlation coefficient is higher than .5.					

**Table 3 tbl3:** Mean JOL Magnitude, Recall Performance and AUROC From Studies 1–3

Condition	Mean JOL magnitude	Recall performance	AUROC
**Study 1**			
Poststudy JOLs	46.61 (16.04)	.51 (.28)	.56 (.08)
Prestudy JOLs	41.97 (15.20)	.53 (.30)	.53 (.08)
**Study 2, experiment 1**			
Immediate	51.22 (22.14)	.46 (.22)	.55 (.09)
Delayed	47.01 (16.90)	.46 (.22)	.80 (.07)
**Study 2, experiment 2**			
Immediate	52.23 (23.02)	.47 (.21)	.57 (.10)
Delayed	41.53 (20.66)	.41 (.23)	.83 (.06)
**Study 2, experiment 3**			
Immediate	61.60 (22.31)	.45 (.20)	.52 (.13)
Delayed	40.56 (20.26)	.36 (.20)	.82 (.08)
**Study 3**			
Old	65.14 (17.45)	.30 (.13)	.50 (.10)
Young	70.05 (16.21)	.30 (.13)	.47 (.09)
*Note*. Standard deviations are reported in parentheses. Recall performance refers to the proportion of recalled word pairs in the memory test.			

**Table 4 tbl4:** Means (and Standard Deviations) of Fitted BIM Parameters From Studies 1–3

Condition	*P*_exp_	*M*_conf_	μ_*m*_	ρ
**Study 1**				
Poststudy JOLs	.30 (.13)	.47 (.16)	.02 (.89)	.22 (.37)
Prestudy JOLs	.24 (.11)	.42 (.15)	.01 (.88)	.00 (.43)
**Study 2, experiment 1**				
Immediate	.46 (.13)	.51 (.22)	−.14 (.69)	.21 (.30)
Delayed	.68 (.21)	.48 (.18)	−.12 (.80)	.80 (.16)
**Study 2, experiment 2**				
Immediate	.51 (.16)	.52 (.23)	−.07 (.60)	.32 (.38)
Delayed	.78 (.17)	.41 (.21)	−.26 (.63)	.84 (.15)
**Study 2, experiment 3**				
Immediate	.45 (.12)	.61 (.22)	−.17 (.62)	.17 (.30)
Delayed	.86 (.14)	.40 (.20)	−.49 (.68)	.85 (.13)
**Study 3**				
Old	.38 (.16)	.65 (.17)	−.55 (.41)	.07 (.30)
Young	.40 (.16)	.70 (.16)	−.55 (.41)	.01 (.32)
*Note*. Standard deviations are reported in parentheses.				

## Footnotes

1 In SDRM, the objective memory strength distribution is called the distribution of memory strength underlying recall, and the processing experience distribution is called the distribution of memory strength underlying confidence judgments (Jang et al., 2012).

2 We thank David E. Huber for raising these important points about SDRM.

3 In a recall task, people give prospective confidence ratings before they take the memory test, and their prospective confidence simply represents the probability that each item will be correctly recalled in the future test, or the posterior probability that the memory strength will be higher than the recall criterion at test. In contrast, when people rate retrospective confidence for each trial in a recall task, they have typically already generated an answer. However, people are usually not completely sure whether their answer is correct or not even if an answer has been generated. Thus, their retrospective confidence represents the probability that each item has been correctly recalled, which should be equal to the posterior probability that the estimated memory strength was higher than the recall criterion in the memory test.

4 Our parameter recovery analysis reveals that when we freely estimate the standard deviation of the noise (σ_noise_) in reported confidence, this parameter cannot be successfully recovered, suggesting that σ_noise_ may be nonidentifiable. Thus, in order to make the model identifiable, we need to set σ_noise_ to an arbitrary value. Following similar assumptions made in a previous study (Fleming et al., 2018), we set σ_noise_ to .025, which allows for a reasonable range of noise for reported confidence. It is likely that the true value for σ_noise_ may differ from .025 in real data sets. However, we are typically not interested in whether σ_noise_ is accurately estimated. Instead, we are more concerned with the other parameters in BIM (*P*_exp_, *M*_conf_, μ_m_, and ρ; see below). To investigate whether these parameters can be accurately estimated when the assumption about σ_noise_ is inaccurate, we performed a parameter recovery analysis with 1,000 simulated data sets from a recall task, where each data set contained 10 trials. When simulating each data set, we randomly sampled σ_noise_ from a range of .005–.1 (which allows an imprecision in reported confidence of approximately ±1%–±20%), and other parameters from the same range as in the parameter recovery analyses below. Here, we set the upper bound of σ_noise_ to .1 because larger noise values suggest random usage of the confidence scale and thus may be unlikely. Then, we constrained σ_noise_ in BIM to .025 and fit the model to simulated data sets. Results showed that all of the four free parameters in BIM could be successfully recovered (*r*_s_ > .75) even when the assumption about σ_noise_ was inaccurate, suggesting that setting σ_noise_ as .025 does not affect the estimation of other parameters.

5 We fix the criteria on the 0–1 *continuous scale* of posterior probability because the model is nonidentifiable when these criteria are allowed to vary. As discussed in this section, the (*n* − 1) criteria on the 0–1 *continuous scale* of posterior probability can be transformed into (*n* − 1) confidence criteria on the distribution of processing experience. If the criteria on the 0–1 *continuous scale* for posterior probability are allowed to vary, then the (*n* − 1) confidence criteria on processing experience distribution are decided by (*n* + 1) free parameters, including σ_*l*_, μ_*b*_ and the (*n* − 1) criteria on the 0–1 scale. Thus, the parameters are redundant and the model is nonidentifiable.

6 BIM is not suitable for fitting confidence data on a 2-point rating scale (i.e., binary scale). This is because the confidence criteria on the processing experience distribution in BIM are determined by two parameters, including *P*_exp_ and μ_*b*_ (although μ_b_ is nonidentifiable and instead we estimate *M*_conf_). Thus, we need at least two criteria (i.e., no less than 3 points on the confidence rating scale) to make the parameters *P*_exp_ and *M*_conf_ identifiable.

7 For mathematical simplicity, here we combine BIM with equal-variance SDT to explain how people rate their retrospective confidence about memory performance in a recognition task. It is possible to combine BIM with unequal-variance SDT to explain the confidence ratings in more realistic memory tasks, in which the variance for the objective memory strength (and perhaps also for the subjective processing experience) may be different between S1 and S2 stimuli (although we leave this extension to future study).

8 We used Spearman correlation rather than Pearson correlation because there were extreme values in fitted parameters such as the confidence criteria.

9 We divided JOLs into 7 bins to make sure none of the criteria for the bins was an integer between 0 and 100 such that no JOLs lay just at the edge of bins.

10 In Studies 1–3, we used *t*-tests to compare the BIM parameters between within-subject conditions when there were no missing data and linear mixed effect model when missing data occurred. The *t* values and partial eta-squared (effect size) from linear mixed effect models are reported. See Hu et al. (2019) for details on building the linear mixed effect models. For consistency in effect size, we report both Cohen’s *d* and partial eta-squared as effect size for classical *t*-tests.

11 As in Study 1, we removed the parameters μ_*m*_ and ρ in the immediate or delayed JOL condition for the participants with the same memory performance for all trials. We also removed the parameters *P*_exp_ and ρ for the participants with the same JOL value for all trials, because during model development we found that the estimation of these parameters is inaccurate when all trials have the same confidence rating.

12 We randomly generated the memory performance (0 or 1) for each trial, and then fitted the restricted BIM to simulated data of confidence ratings and memory performance.
