# Supplementary material for: A Bayesian Inference Model for Metamemory
Source: Psychol Rev. 2021 May 27;128(5):824–55. doi: 10.1037/rev0000270 (PMC9006386; doi:10.1037/rev0000270)

**Supplemental Materials**

**S1. Mean of the confidence distribution**

In BIM, we can calculate the mean of the confidence distribution. According to the assumption of BIM, reported confidence on a 0-1 continuous scale is the sum of the predicted confidence rating based on the posterior distribution of estimated memory strength *m̂* and a noise term with a mean of 0. Thus, the mean of reported confidence is equal to the sum of the means for predicted confidence and noise:

| $M_{conf}=E\left( predicted conf \right)+E\left( noise \right)$ | (1) |
| --- | --- |

in which the mean of noise is 0. Thus, the mean of reported confidence *M_conf_* is the same as the mean of predicted confidence based on the posterior distribution of estimated memory strength. Here we first discuss *M_conf_* in BIM for recall tasks, in which the predicted confidence is the posterior probability that estimated memory strength *m̂* is higher than 0, i.e. *P* (*m̂* > 0 | *e*).

To calculate mean confidence, we need to first mathematically characterize the confidence distribution. According to BIM, the posterior distribution of memory strength *m̂* comes from a Bayesian inference based on the processing experience *e*:

| $f\left( \hat{m}\vert e \right)=\frac{f\left( e\vert\hat{m} \right)f\left( \hat{m} \right)}{f\left( e \right)}$ | (2) |
| --- | --- |

in which the prior distribution is a normal distribution with a mean of *μ_b_* and a standard deviation of 1:

| $\hat{m}\sim N\left( \mu_{b},1^{2} \right)$ | (3) |
| --- | --- |

and the likelihood function is the density of another normal distribution with a mean of *m̂* and a standard deviation of *σ_l_*:

| $e\vert\hat{m}\sim N\left( \hat{m},{\sigma_{l}}^{2} \right)$ | (4) |
| --- | --- |

The normal prior distribution is the conjugate prior of the normal distribution, and the posterior distribution is another normal distribution (Murphy, 2007). We can then calculate the mean and variance of the posterior distribution based on the conjugate prior:

| $E\left( \hat{m} \vert e \right)=\frac{e+{\sigma_{l}}^{2}\mu_{b}}{1+{\sigma_{l}}^{2}}$ | (5) |
| --- | --- |
| $Var\left( \hat{m} \vert e \right)=\frac{{\sigma_{l}}^{2}}{1+{\sigma_{l}}^{2}}$ | (6) |

We can calculate the predicted confidence rating based on the posterior distribution:

| $predicted conf=P\left( \hat{m}>0 \vert e \right)=\Phi\left( \frac{E\left( \hat{m} \vert e \right)}{\sqrt{Var\left( \hat{m} \vert e \right)}} \right)=\Phi\left( \frac{e+{\sigma_{l}}^{2}\mu_{b}}{\sigma_{l}\sqrt{1+{\sigma_{l}}^{2}}} \right)$ | (7) |
| --- | --- |

in which Φ is the cumulative density function of the standard normal distribution.

According to BIM, the processing experience *e* is derived from the processing experience distribution with a mean of *μ_e_* and a standard deviation of 1:

| $e\sim N\left( \mu_{e},1 \right)$ | (8) |
| --- | --- |

Let *e* = *x_0_* + *μ_e_*, in which *x_0_* is distributed as a standard normal distribution. Then the predicted confidence is equal to:

| $predicted conf=\Phi\left( \frac{x_{0}+\mu_{e}+{\sigma_{l}}^{2}\mu_{b}}{\sigma_{l}\sqrt{1+{\sigma_{l}}^{2}}} \right)$ $=\Phi\left( \frac{1}{\sigma_{l}\sqrt{1+{\sigma_{l}}^{2}}}x_{0}+\frac{\frac{\mu_{e}}{\sigma_{l}}+\mu_{b}\sigma_{l}}{\sqrt{1+{\sigma_{l}}^{2}}} \right)$ | (9) |
| --- | --- |

Define two new parameters *A* and *B*:

| $A=\frac{1}{\sigma_{l}\sqrt{1+{\sigma_{l}}^{2}}}$ | (10) |
| --- | --- |
| $B=\frac{\frac{\mu_{e}}{\sigma_{l}}+\mu_{b}\sigma_{l}}{\sqrt{1+{\sigma_{l}}^{2}}}$ | (11) |

Then we obtain:

| $predicted conf=\Phi\left( Ax_{0}+B \right)$ | (12) |
| --- | --- |
| $x_{0}=\frac{\Phi^{-1}\left( predicted conf \right)-B}{A}$ | (13) |

in which Φ^-1^ is the inverse function of the cumulative density function for the standard normal distribution.

According to the probability integral transform, we know that (Angus, 2005):

| $P\left( \Phi\left( x_{0} \right)<x \right)=x$ | (14) |
| --- | --- |

Thus, the cumulative density function for the distribution of predicted confidence is:

| $F_{conf}\left( x \right)=P\left( predicted conf<x \right)=P\left( \Phi\left( Ax_{0}+B \right)<x \right)$ $=P\left( x_{0}<\frac{\Phi^{-1}\left( x \right)-B}{A} \right)$ $=P\left[ \Phi\left( x_{0} \right)<\Phi\left( \frac{\Phi^{-1}\left( x \right)-B}{A} \right) \right]=\Phi\left( \frac{\Phi^{-1}\left( x \right)-B}{A} \right)$ | (15) |
| --- | --- |

in which *x* is between 0 and 1.

We can then calculate the mean of the predicted confidence distribution:

| $E\left( predicted conf \right)=\int_{0}^{1} xf_{conf}\left( x \right)dx=\int_{0}^{1} xd\left[ F_{conf}\left( x \right) \right]$ $=\left\vert{1 \atop0}xF_{conf}\left( x \right) \right.-\int_{0}^{1} F_{conf}\left( x \right)dx$ $=1-\int_{0}^{1} \Phi\left( \frac{\Phi^{-1}\left( x \right)-B}{A} \right)dx$ | (16) |
| --- | --- |

Next we will prove that the second term of Equation (16) (i.e., the integration) can be computed based on the area under the receiver operating characteristic curve (AUC) in signal detection theory (Stanislaw & Todorov, 1999).

Suppose that in a signal detection process, the signal distribution has a mean of *d_1_* and a standard deviation of 1, and the noise distribution has a mean of 0 and a standard deviation of *σ_N_*. The response criterion is *c_1_* (note that the *d_1_* and *c_1_* discussed here are not the same as the parameters *d’* and *C* when BIM is fitted to data from recognition tasks). The hit rate (HR) and false alarm rate (FAR) can be calculated as:

| $HR=\Phi\left( d_{1}-c_{1} \right)$ | (17) |
| --- | --- |
| $FAR=\Phi\left( -c_{1}/\sigma_{N} \right)$ | (18) |

Eliminating *c_1_*, we get

| $\Phi^{-1}\left( HR \right)=\sigma_{N}\Phi^{-1}\left( FAR \right)+d_{1}$ | (19) |
| --- | --- |
| $HR=\Phi\left( \sigma_{N}\Phi^{-1}\left( FAR \right)+d_{1} \right)$ | (20) |

Then the AUC is equal to

| $AUC=\int_{0}^{1} HR\cdot d\left( FAR \right)=\int_{0}^{1} \Phi\left( \sigma_{N}\Phi^{-1}\left( FAR \right)+d_{1} \right)d\left( FAR \right)$ | (21) |
| --- | --- |

Let *d_1_* = -*B* / *A*, *σ_N_* = 1 / *A*. We can get

| $AUC=\int_{0}^{1} \Phi\left( \frac{\Phi^{-1}\left( FAR \right)-B}{A} \right)d\left( FAR \right)$ | (22) |
| --- | --- |

which is the same as the second term in Equation (16).

From previous work (Stanislaw & Todorov, 1999), we also know that

| $AUC=\Phi\left( \frac{d_{1}}{\sqrt{1+{\sigma_{N}}^{2}}} \right)=\Phi\left( \frac{-B}{\sqrt{1+A^{2}}} \right)$ | (23) |
| --- | --- |

Substituting Equations (22) and (23) above into Equation (16), we can get the mean of predicted confidence distribution:

| $E\left( predicted conf \right)=1-\Phi\left( \frac{-B}{\sqrt{1+A^{2}}} \right)=\Phi\left( \frac{B}{\sqrt{1+A^{2}}} \right)$ $=\Phi\left( \frac{\frac{\mu_{e}}{\sigma_{l}}+\mu_{b}\sigma_{l}}{\sqrt{\sigma_{l}^{2}+1/{\sigma_{l}^{2}}+1}} \right)$ | (24) |
| --- | --- |

which is also the mean of reported confidence (i.e., *M_conf_*).

We can then calculate the proportions for the contribution of processing experience (*P_exp_*) and prior beliefs (*P_belief_*) to confidence ratings based on *σ_l_* (see main text for details), and the product of these two proportions:

| $P_{exp}=\frac{1}{1+{\sigma_{l}}^{2}}=\frac{\frac{1}{\sigma_{l}}}{\frac{1}{\sigma_{l}}+\sigma_{l}}$ | (25) |
| --- | --- |
| $P_{belief}=\frac{{\sigma_{l}}^{2}}{1+{\sigma_{l}}^{2}}=\frac{\sigma_{l}}{\frac{1}{\sigma_{l}}+\sigma_{l}}$ | (26) |
| $P_{exp}P_{belief}=\frac{1}{\left( \frac{1}{\sigma_{l}}+\sigma_{l} \right)^{2}}$ | (27) |

Substituting Equations (25)-(27) into Equation (24), we can obtain:

| $M_{conf}=\Phi\left( \frac{\frac{\mu_{e}}{\sigma_{l}}+\mu_{b}\sigma_{l}}{\sqrt{\sigma_{l}^{2}+1/{\sigma_{l}^{2}}+1}} \right)$ $=\Phi\left[ \left( \mu_{e}\cdot\frac{\frac{1}{\sigma_{l}}}{\frac{1}{\sigma_{l}}+\sigma_{l}}+\mu_{b}\cdot\frac{\sigma_{l}}{\frac{1}{\sigma_{l}}+\sigma_{l}} \right)\sqrt{\frac{\left( \frac{1}{\sigma_{l}}+\sigma_{l} \right)^{2}}{\left( \frac{1}{\sigma_{l}}+\sigma_{l} \right)^{2}-1}} \right]$ $=\Phi\left( \frac{\mu_{e}P_{exp}+\mu_{b}P_{belief}}{\sqrt{1-P_{exp}P_{belief}}} \right)$ | (28) |
| --- | --- |

Equation (28) represents the parameter *M_conf_* in BIM for recall tasks. When we fit BIM to recognition tasks with retrospective confidence ratings, the confidence rating predicted by BIM is the posterior probability that memory strength *m̂* is lower than the Type I criterion *C* when people give an S1 response, and higher than *C* when they give an S2 response:

| $predicted {conf}_{rS1}=P\left( \hat{m}<C \vert e \right)=\Phi\left( \frac{C-E\left( \hat{m} \vert e \right)}{\sqrt{Var\left( \hat{m} \vert e \right)}} \right)$ $=\Phi\left( -\frac{1}{\sigma_{l}\sqrt{1+{\sigma_{l}}^{2}}}x_{0}-\frac{\frac{\mu_{e}}{\sigma_{l}}+\mu_{b}\sigma_{l}}{\sqrt{1+{\sigma_{l}}^{2}}}+\frac{C\sqrt{1+{\sigma_{l}}^{2}}}{\sigma_{l}} \right)$ | (29) |
| --- | --- |
| $predicted {conf}_{rS2}=P\left( \hat{m}>C \vert e \right)=\Phi\left( \frac{E\left( \hat{m} \vert e \right)-C}{\sqrt{Var\left( \hat{m} \vert e \right)}} \right)$ $=\Phi\left( \frac{1}{\sigma_{l}\sqrt{1+{\sigma_{l}}^{2}}}x_{0}+\frac{\frac{\mu_{e}}{\sigma_{l}}+\mu_{b}\sigma_{l}}{\sqrt{1+{\sigma_{l}}^{2}}}-\frac{C\sqrt{1+{\sigma_{l}}^{2}}}{\sigma_{l}} \right)$ | (30) |

Define two new parameters *A* and *B* (separately for S1 and S2 response):

| $A_{rS1}=-\frac{1}{\sigma_{l}\sqrt{1+{\sigma_{l}}^{2}}}$ | (31) |
| --- | --- |
| $A_{rS2}=\frac{1}{\sigma_{l}\sqrt{1+{\sigma_{l}}^{2}}}$ | (32) |
| $B_{rS1}=-\frac{\frac{\mu_{e}}{\sigma_{l}}+\mu_{b}\sigma_{l}}{\sqrt{1+{\sigma_{l}}^{2}}}+\frac{C\sqrt{1+{\sigma_{l}}^{2}}}{\sigma_{l}}$ | (33) |
| $B_{rS2}=\frac{\frac{\mu_{e}}{\sigma_{l}}+\mu_{b}\sigma_{l}}{\sqrt{1+{\sigma_{l}}^{2}}}-\frac{C\sqrt{1+{\sigma_{l}}^{2}}}{\sigma_{l}}$ | (34) |

In the main text, we have demonstrated that *μ_e_* is different between S1 and S2 stimulus, and *μ_b_* is different between S1 and S2 response. Thus, the value of parameter *B* is different across the 2 (stimulus: S1 vs. S2) × 2 (response: S1 vs. S2) conditions:

| $B_{sS1,rS1}=-\frac{\frac{\mu_{e\left( sS1 \right)}}{\sigma_{l}}+\mu_{b\left( rS1 \right)}\sigma_{l}}{\sqrt{1+{\sigma_{l}}^{2}}}+\frac{C\sqrt{1+{\sigma_{l}}^{2}}}{\sigma_{l}}$ | (35) |
| --- | --- |
| $B_{sS1,rS2}=\frac{\frac{\mu_{e\left( sS1 \right)}}{\sigma_{l}}+\mu_{b\left( rS2 \right)}\sigma_{l}}{\sqrt{1+{\sigma_{l}}^{2}}}-\frac{C\sqrt{1+{\sigma_{l}}^{2}}}{\sigma_{l}}$ | (36) |
| $B_{sS2,rS1}=-\frac{\frac{\mu_{e\left( sS2 \right)}}{\sigma_{l}}+\mu_{b\left( rS1 \right)}\sigma_{l}}{\sqrt{1+{\sigma_{l}}^{2}}}+\frac{C\sqrt{1+{\sigma_{l}}^{2}}}{\sigma_{l}}$ | (37) |
| $B_{sS2,rS2}=\frac{\frac{\mu_{e\left( sS2 \right)}}{\sigma_{l}}+\mu_{b\left( rS2 \right)}\sigma_{l}}{\sqrt{1+{\sigma_{l}}^{2}}}-\frac{C\sqrt{1+{\sigma_{l}}^{2}}}{\sigma_{l}}$ | (38) |

Then we can compute the parameter *M_conf_* separately for each of the four conditions:

| $M_{conf\left( sS1,rS1 \right)}=\Phi\left( \frac{B_{sS1,rS1}}{\sqrt{1+{A_{rS1}}^{2}}} \right)$ $=\Phi\left( -\frac{\mu_{e\left( sS1 \right)}P_{exp}+\mu_{b\left( rS1 \right)}P_{belief}-C}{\sqrt{1-P_{exp}P_{belief}}} \right)$ | (39) |
| --- | --- |
| $M_{conf\left( sS1,rS2 \right)}=\Phi\left( \frac{B_{sS1,rS2}}{\sqrt{1+{A_{rS2}}^{2}}} \right)$ $=\Phi\left( \frac{\mu_{e\left( sS1 \right)}P_{exp}+\mu_{b\left( rS2 \right)}P_{belief}-C}{\sqrt{1-P_{exp}P_{belief}}} \right)$ | (40) |
| $M_{conf\left( sS2,rS1 \right)}=\Phi\left( \frac{B_{sS2,rS1}}{\sqrt{1+{A_{rS1}}^{2}}} \right)$ $=\Phi\left( -\frac{\mu_{e\left( sS2 \right)}P_{exp}+\mu_{b\left( rS1 \right)}P_{belief}-C}{\sqrt{1-P_{exp}P_{belief}}} \right)$ | (41) |
| $M_{conf\left( sS2,rS2 \right)}=\Phi\left( \frac{B_{sS2,rS2}}{\sqrt{1+{A_{rS2}}^{2}}} \right)$ $=\Phi\left( \frac{\mu_{e\left( sS2 \right)}P_{exp}+\mu_{b\left( rS2 \right)}P_{belief}-C}{\sqrt{1-P_{exp}P_{belief}}} \right)$ | (42) |

**S2. Maximum likelihood estimation of BIM with confidence on continuous scale**

In this section, we show how to estimate the parameters in BIM with maximum likelihood estimation when people report confidence on a continuous scale from 0 to 1. From the assumptions of BIM and the calculations in Section S1 (see Equation (12)), we know that:

| $predicted conf=\Phi\left( Ax_{0}+B \right)$ | (43) |
| --- | --- |
| $reported conf\sim N\left( predicted conf,{0.025}^{2} \right)$ | (44) |

in which *x_0_* comes from a standard normal distribution (which is perfectly correlated with the processing experience distribution). Thus, the probability density of reported confidence given *x_0_* is:

| $f\left( reported conf \vert x_{0} \right)=f_{N}\left( reported conf \vert\mu=\Phi\left( Ax_{0}+B \right),\sigma=0.025 \right)$ | (45) |
| --- | --- |

in which *f_N_* is the probability density function of normal distribution. We should note that when BIM is fitted to data from recognition tasks with retrospective confidence ratings, the value of parameters *A* and *B* is different across the 2 (stimulus: S1 vs. S2) × 2 (response: S1 vs. S2) conditions (see Section S1).

On the other hand, BIM assumes that the memory performance for each trial depends on the comparison between objective memory strength and a criterion. We first discuss memory performance in BIM for recall tasks, in which the distribution of objective memory strength has a mean of *μ_m_* and a standard deviation of 1, and an item can be correctly recalled if its memory strength is higher than 0. We also know that the correlation between the distributions of processing experience and objective memory strength is *ρ*. Thus, the *x_0_* distribution and the objective memory strength distribution constructs a bivariate normal distribution with a correlation of *ρ*. The conditional distribution of objective memory strength given *x_0_* is:

| $f\left( m \vert x_{0} \right)=f_{N}\left( m \vert\mu=\mu_{m}+\rho x_{0},\sigma=\sqrt{1-\rho^{2}} \right)$ | (46) |
| --- | --- |

Thus, the probability of correct recall given *x_0_* is:

| $P\left( correct \vert x_{0} \right)= P\left( m>0 \vert x_{0} \right)=P_{N}\left( m>0 \vert\mu=\mu_{m}+\rho x_{0},\sigma=\sqrt{1-\rho^{2}} \right)$ | (47) |
| --- | --- |

in which *P_N_* represents the probability under a normal distribution.

Next, we discuss memory performance in BIM for recognition tasks, in which the mean of objective memory strength for S1 and S2 stimuli are -*d’*/2 and *d’*/2, respectively. People can give a correct S1 response when the objective memory strength for S1 stimulus is lower than the Type I criterion *C*, and a correct S2 response when the memory strength for S2 stimulus is higher than *C*. Thus, we can compute the probability of a correct response given *x_0_* separately for S1 and S2 stimuli:

| $P\left( correct \vert x_{0},sS1 \right)= P\left( m<C \vert x_{0},sS1 \right)$ $=P_{N}\left( m<C \vert\mu=-\frac{d'}{2}+\rho x_{0},\sigma=\sqrt{1-\rho^{2}} \right)$ | (48) |
| --- | --- |
| $P\left( correct \vert x_{0},sS2 \right)= P\left( m>C \vert x_{0},sS2 \right)$ $=P_{N}\left( m>C \vert\mu=\frac{d'}{2}+\rho x_{0},\sigma=\sqrt{1-\rho^{2}} \right)$ | (49) |

To calculate the likelihood for a trial with a certain confidence rating and memory performance (0 or 1), we need to integrate over *x_0_*:

| $L\left( reported conf,Mem \right)=\int_{-\infty}^{\infty} f\left( reported conf \vert x_{0} \right)\left\{ Mem\cdot P\left( correct \vert x_{0} \right) \right.$ $+\left. \left( 1-Mem \right)\left[ 1-P\left( correct \vert x_{0} \right) \right] \right\}f_{N}\left( x_{0} \right)dx_{0}$ | (50) |
| --- | --- |

in which *Mem* is the memory performance (0 or 1) obtained from empirical data.

Finally, we can compute the log likelihood *l* for a dataset with *n* trials and find the parameter values that maximize the log likelihood:

| $l=\sum_{i=1}^{n} \ln L\left( {reported conf}_{i},{Mem}_{i} \right)$ | (51) |
| --- | --- |

**S3. Tradeoff between the standard deviations and other parameters in BIM**

In BIM, we set the standard deviations to 1 for three separate distributions, including the distributions of objective memory strength (*σ_m_*), processing experience (*σ_e_*) and prior belief (*σ_b_*). These standard deviation parameters are constrained to make the model identifiable. In this section, we discuss how the change in these standard deviations can be traded off by other parameters in BIM when we allow the standard deviation parameters to vary.

We first explain the relationship between *σ_m_* and *μ_m_* (mean of the objective memory strength distribution) in BIM for recall tasks. In Section S2, we already know that the correlation between *x_0_* and the objective memory strength *m* is *ρ*. When *σ_m_* is allowed to vary, we can obtain the distribution of *m* given *x_0_*:

| $f\left( m \vert x_{0} \right)=f_{N}\left( m \vert\mu=\mu_{m}+\rho\sigma_{m}x_{0},\sigma=\sigma_{m}\sqrt{1-\rho^{2}} \right)$ | (52) |
| --- | --- |

The probability of correct recall for each trial given *x_0_* is the probability that *m* is higher than 0:

| $P\left( correct \vert x_{0} \right)= P\left( m>0 \vert x_{0} \right)$ $=P_{N}\left( m>0 \vert\mu=\mu_{m}+\rho\sigma_{m}x_{0},\sigma=\sigma_{m}\sqrt{1-\rho^{2}} \right)$ $=\Phi\left( \frac{\mu_{m}}{\sigma_{m}\sqrt{1-\rho^{2}}}+\frac{\rho x_{0}}{\sqrt{1-\rho^{2}}} \right)$ | (53) |
| --- | --- |

From Equation (53), we note that *σ_m_* is simply a scaling parameter for *μ_m_*. For example, when *σ_m_* is set to 2 rather than 1, we only need to multiply the value of *μ_m_* by 2 to make the model predictions of memory performance unchanged. For mathematical simplicity, we set *σ_m_* to 1 in the BIM described in the main text.

Similarly, in BIM for recognition tasks, *σ_m_* represents the standard deviation of objective memory strength for S1 and S2 stimuli, and is a scaling parameter for *d’* and *C* in signal detection theory.

We then discuss the tradeoff between *σ_e_*, *σ_b_* and other parameters in BIM. When *σ_e_* and *σ_b_* are allowed to vary, the mean and variance for the posterior distribution of estimated memory strength *m̂* are (similar to Equations (5) and (6)):

| $E\left( \hat{m} \vert e \right)=\frac{{\sigma_{b}}^{2}e+{\sigma_{l}}^{2}\mu_{b}}{{\sigma_{b}}^{2}+{\sigma_{l}}^{2}}=\frac{{\sigma_{b}}^{2}\left( \sigma_{e}x_{0}+\mu_{e} \right)+{\sigma_{l}}^{2}\mu_{b}}{{\sigma_{b}}^{2}+{\sigma_{l}}^{2}}$ | (54) |
| --- | --- |
| $Var\left( \hat{m} \vert e \right)=\frac{{\sigma_{l}}^{2}{\sigma_{b}}^{2}}{{\sigma_{b}}^{2}+{\sigma_{l}}^{2}}$ | (55) |

in which *x_0_* comes from a standard normal distribution and is perfectly correlated with the distribution of processing experience.

For BIM applied to recall tasks, the predicted confidence is:

| $predicted conf=P\left( \hat{m}>0 \vert e \right)$ $=\Phi\left( \frac{\sigma_{b}\sigma_{e}}{\sigma_{l}\sqrt{{\sigma_{b}}^{2}+{\sigma_{l}}^{2}}}x_{0}+\frac{{\sigma_{b}}^{2}\mu_{e}+{\sigma_{l}}^{2}\mu_{b}}{\sigma_{l}\sigma_{b}\sqrt{{\sigma_{b}}^{2}+{\sigma_{l}}^{2}}} \right)=\Phi\left( Ax_{0}+B \right)$ | (56) |
| --- | --- |

in which the parameters *A* and *B* are:

| $A=\frac{\sigma_{b}\sigma_{e}}{\sigma_{l}\sqrt{{\sigma_{b}}^{2}+{\sigma_{l}}^{2}}}$ | (57) |
| --- | --- |
| $B=\frac{{\sigma_{b}}^{2}\mu_{e}+{\sigma_{l}}^{2}\mu_{b}}{\sigma_{l}\sigma_{b}\sqrt{{\sigma_{b}}^{2}+{\sigma_{l}}^{2}}}$ | (58) |

For BIM applied to recognition asks, the predicted confidence for S1 and S2 response is:

| $predicted {conf}_{rS1}=P\left( \hat{m}<C \vert e \right)$ $=\Phi\left( -\frac{\sigma_{b}\sigma_{e}}{\sigma_{l}\sqrt{{\sigma_{b}}^{2}+{\sigma_{l}}^{2}}}x_{0}-\frac{{\sigma_{b}}^{2}\mu_{e}+{\sigma_{l}}^{2}\mu_{b}}{\sigma_{l}\sigma_{b}\sqrt{{\sigma_{b}}^{2}+{\sigma_{l}}^{2}}} \right.$ $\left. +\frac{C\sqrt{{\sigma_{b}}^{2}+{\sigma_{l}}^{2}}}{\sigma_{l}\sigma_{b}} \right)$ $=\Phi\left( A_{rS1}x_{0}+B_{rS1} \right)$ | (59) |
| --- | --- |
| $predicted {conf}_{rS2}=P\left( \hat{m}>C \vert e \right)$ $=\Phi\left( \frac{\sigma_{b}\sigma_{e}}{\sigma_{l}\sqrt{{\sigma_{b}}^{2}+{\sigma_{l}}^{2}}}x_{0}+\frac{{\sigma_{b}}^{2}\mu_{e}+{\sigma_{l}}^{2}\mu_{b}}{\sigma_{l}\sigma_{b}\sqrt{{\sigma_{b}}^{2}+{\sigma_{l}}^{2}}} \right.$ $\left. -\frac{C\sqrt{{\sigma_{b}}^{2}+{\sigma_{l}}^{2}}}{\sigma_{l}\sigma_{b}} \right)$ $=\Phi\left( A_{rS2}x_{0}+B_{rS2} \right)$ | (60) |

in which the parameters *A* and *B* for S1 and S2 response are:

| $A_{rS1}=-\frac{\sigma_{b}\sigma_{e}}{\sigma_{l}\sqrt{{\sigma_{b}}^{2}+{\sigma_{l}}^{2}}}$ | (61) |
| --- | --- |
| $A_{rS2}=\frac{\sigma_{b}\sigma_{e}}{\sigma_{l}\sqrt{{\sigma_{b}}^{2}+{\sigma_{l}}^{2}}}$ | (62) |
| $B_{rS1}=-\frac{{\sigma_{b}}^{2}\mu_{e}+{\sigma_{l}}^{2}\mu_{b}}{\sigma_{l}\sigma_{b}\sqrt{{\sigma_{b}}^{2}+{\sigma_{l}}^{2}}}+\frac{C\sqrt{{\sigma_{b}}^{2}+{\sigma_{l}}^{2}}}{\sigma_{l}\sigma_{b}}$ | (63) |
| $B_{rS2}=\frac{{\sigma_{b}}^{2}\mu_{e}+{\sigma_{l}}^{2}\mu_{b}}{\sigma_{l}\sigma_{b}\sqrt{{\sigma_{b}}^{2}+{\sigma_{l}}^{2}}}-\frac{C\sqrt{{\sigma_{b}}^{2}+{\sigma_{l}}^{2}}}{\sigma_{l}\sigma_{b}}$ | (64) |

The parameters *A* and *B* can be seen as the scale and location parameters for *x_0_*, and the model predictions on confidence ratings are only determined by *A* and *B*. In addition, *A* is determined by parameters *σ_e_*, *σ_b_* and *σ_l_* (standard deviation of the likelihood function in Bayesian inference), and *B* is determined by *σ_b_*, *σ_l_*, *μ_e_* (mean of the processing experience distribution) and *μ_b_* (mean of the prior belief distribution). Thus, a change in *σ_e_* and *σ_b_* can be mimicked by a change in *σ_l_*, *μ_e_* and *μ_b_* to leave *A* and *B* unchanged, leading to unchanged model predictions on confidence ratings (we should note that the values of A and B are unrelated to the parameters *μ_m_* and *ρ* in BIM).

We know that the parameters *μ_e_* and *μ_b_* in BIM are not identifiable, and instead we estimate *M_conf_*. When *A* and *B* remain the same, the value of *M_conf_* is not affected by the change in *σ_e_* and *σ_b_*. This is because *M_conf_* can be computed as (see also Equation (24)):

| $M_{conf}=\Phi\left( \frac{B}{\sqrt{1+A^{2}}} \right)$ | (65) |
| --- | --- |

Thus, the value of *M_conf_* is unique when *A* and *B* are given regardless of the values of *σ_e_* and *σ_b_*.

Next, we explain how a change in *σ_e_* and *σ_b_* can be mimicked by a change in *σ_l_*. We already know that the parameter *A* is determined by *σ_e_*, *σ_b_* and *σ_l_* (see Equations (57), (61) and (62)). When *σ_e_* is equal to *σ_b_*, these two parameters can be seen as scaling parameters for *σ_l_*. For example, when *σ_e_* and *σ_b_* are set to 2 rather than 1, we only need to multiply the value of *σ_l_* by 2 to leave parameter *A* unchanged. When *σ_e_* and *σ_b_* are unequal, we can still adjust the value of *σ_l_* to mimic the effect of these two parameters on the value of *A*. For example, increasing the value of either *σ_e_* or *σ_b_* can be mimicked by decreasing *σ_l_*. However, in this case, *σ_e_* and *σ_b_* are not simply the scaling parameters for *σ_l_*.

One specific case obtains when we fix the value of *σ_e_* and allow *σ_b_* to vary. The standard deviation of the prior belief distribution represents the certainty of prior beliefs, and *σ_b_* is higher when people are more uncertain of their beliefs about overall memory ability. From the discussion above, we know that when the standard deviation of the likelihood function (*σ_l_*) is decreased, this effect on model predictions of confidence ratings can be mimicked by increasing belief uncertainty (and vice versa). In other words, if we fit BIM (in which both *σ_e_* and *σ_b_* are constrained to 1) to empirical datasets in two experimental conditions and find that the fitted value of *σ_l_* in Condition 1 is lower than Condition 2 (or the fitted value of *P_exp_* in Condition 1 is higher than Condition 2; see Equation (25)), there are two possibilities for this difference: (a) participants in Condition 1 assume there is a more close relationship between processing experience and memory strength (i.e., *σ_l_* in Condition 1 is indeed lower than Condition 2), or (b) participants in Condition 1 are actually more uncertain of their beliefs about their memory ability (i.e., *σ_b_* in Condition 1 is higher than Condition 2).

In the main text, we have discussed an extension of BIM which accounts for the updating of beliefs about memory ability during the metamemory process. Results from data simulation suggest that *σ_b_* decreases across trials in this extended BIM, suggesting that people become more certain about their beliefs about memory ability during belief updating. The extended BIM cannot be directly fitted to data because *σ_b_* is allowed to vary across trials, which makes the parameters redundant and the model non-identifiable. However, if we fit the restricted version of BIM (in which *σ_b_* is constrained to 1) to simulated data, we can see that the fitted value of *σ_l_* increases across trials (or fitted *P_exp_* decreases across trials) even when the true value of *σ_l_* in the extended BIM does not change. The increase of fitted *σ_l_* in the restricted BIM does not indicate that participants assume processing experience is less closely distributed around memory strength in later trials. Instead, it is because the decrease of belief uncertainty (*σ_b_*) across trials reduces the variance of the confidence distribution, which can be mimicked by increasing *σ_l_* in the restricted BIM.

**S4. Maximum likelihood estimation of BIM with confidence on discrete scale**

In this section, we show how to estimate the parameters in BIM with maximum likelihood estimation when people report confidence on a *n*-point discrete scale.

We first discuss the likelihood function for BIM applied to recall tasks. In the main text, we have demonstrated that the criteria on 0-1 continuous confidence scale (*C_conf_*) can be transformed into the confidence criteria on the processing experience distribution (*C_e_*):

| $\begin{matrix} C_{conf\left( i \right)}=\Phi\left( \frac{C_{e\left( i \right)}+{\sigma_{l}}^{2}\mu_{b}}{\sigma_{l}\sqrt{1+{\sigma_{l}}^{2}}} \right) & 1\leq i\leq n-1 \end{matrix}$ | (66) |
| --- | --- |

Let *C_e(i)_* = *C_i_* + *μ_e_*, in which the *C_i_*s are the criteria on the distribution of *x_0_*, a standard normal distribution perfectly correlated with the distribution of processing experience (see Sections S1 and S2 for more details about *x_0_*). Then the *C_conf_*s can be transformed into confidence criteria on the distribution of *x_0_*:

| $\begin{matrix} C_{conf\left( i \right)}=\Phi\left( \frac{C_{i}+\mu_{e}+{\sigma_{l}}^{2}\mu_{b}}{\sigma_{l}\sqrt{1+{\sigma_{l}}^{2}}} \right)=\Phi\left( AC_{i}+B \right) & 1\leq i\leq n-1 \end{matrix}$ | (67) |
| --- | --- |
| $\begin{matrix} C_{i}=\frac{\Phi^{-1}\left( C_{conf\left( i \right)} \right)-B}{A} & 1\leq i\leq n-1 \end{matrix}$ | (68) |

in which the parameters *A* and *B* are the same as those in Section S1 (see Equations (10)-(11)).

BIM also assumes that the objective memory strength *m* comes from a distribution of *μ_m_* with a recall criterion of 0. The probability of correct recall can be computed as:

| $P_{N}\left( m>0 \vert\mu=\mu_{m},\sigma=1 \right)=P_{N}\left( m>-\mu_{m} \vert\mu=0,\sigma=1 \right)$ | (69) |
| --- | --- |

Thus, the distribution of objective memory strength can be seen as a standard normal distribution with a recall criterion of -*μ_m_*.

We also know that the correlation between processing experience and objective memory strength is *ρ*. Thus, the *x_0_* distribution and the transformed distribution of objective memory strength constructs a standard bivariate normal distribution with a correlation of *ρ*. We can then partition the area under this standard bivariate normal distribution to calculate the probability of each confidence bin for recalled and unrecalled items. Define the cumulative density function of a standard bivariate normal distribution with correlation coefficient *ρ* as *F (x, y, ρ)*. Then we can get:

| $P_{unrecalled,conf=1}=P\left( m<-\mu_{m},x_{0}<C_{1} \right)=F\left( -\mu_{m},C_{1},\rho\right)$ | (70) |
| --- | --- |
| $P_{recalled,conf=1}=P\left( m>-\mu_{m},x_{0}<C_{1} \right)=\Phi\left( C_{1} \right)-F\left( -\mu_{m},C_{1},\rho\right)$ | (71) |
| $P_{unrecalled,conf=i\left( 1<i<n \right)}=P\left( m<-\mu_{m},{C_{i-1}<x}_{0}<C_{i} \right)$ $=F\left( -\mu_{m},C_{i},\rho\right)-F\left( -\mu_{m},C_{i-1},\rho\right)$ | (72) |
| $P_{recalled,conf=i\left( 1<i<n \right)}=P\left( m>-\mu_{m},{C_{i-1}<x}_{0}<C_{i} \right)$ $=\left[ \Phi\left( C_{i} \right)-F\left( -\mu_{m},C_{i},\rho\right) \right]-\left[ \Phi\left( C_{i-1} \right)-F\left( -\mu_{m},C_{i-1},\rho\right) \right]$ | (73) |
| $P_{unrecalled,conf=n}=P\left( m<-\mu_{m},x_{0}>C_{n-1} \right)$ $=\Phi\left( -\mu_{m} \right)-F\left( -\mu_{m},C_{n-1},\rho\right)$ | (74) |
| $P_{recalled,conf=n}=P\left( m>-\mu_{m},x_{0}>C_{n-1} \right)$ $=1-\Phi\left( C_{n-1} \right)-\left[ \Phi\left( -\mu_{m} \right)-F\left( -\mu_{m},C_{n-1},\rho\right) \right]$ | (75) |

Finally, we can calculate the log likelihood of the data and use maximum likelihood estimation to estimate the parameters in BIM:

| $l=\sum_{i=1}^{n} N_{recalled,conf=i}\ln\left( P_{recalled,conf=i} \right)$ $+\sum_{i=1}^{n} N_{unrecalled,conf=i}\ln\left( P_{unrecalled,conf=i} \right)$ | (76) |
| --- | --- |

in which *N* represents the number of trials in each confidence category for recalled and unrecalled conditions.

The likelihood function for BIM applied to recognition tasks is similar to that for BIM applied to recall tasks except for two differences. First, the mean for the objective memory strength distribution is -*d’*/2 for S1 stimuli and *d’*/2 for S2 stimuli, and memory performance depends on the comparison between objective memory strength and the Type I criterion *C* in signal detection theory (rather than 0). A trial with an S1 stimulus can only be correctly answered when objective memory strength *m* is lower than *C*, and a trial with an S2 stimulus can be correctly answered when *m* is higher than *C*. Second, the parameters *A* and *B* are different across the 2 (stimulus: S1 vs. S2) × 2 (response: S1 vs. S2) conditions (see Equations (31)-(38) in Section S1).

**S5. Using simulated data from SDRM and the meta-d’ model to examine the relationship between BIM and these models**

In this section, we simulated data from both SDRM and the meta-d’ model to examine the relationship between the parameters in BIM and these two models. We first used SDRM to simulate confidence ratings (on a 7-point scale) and memory performance (0 or 1) from a recall test, and compared the parameters in BIM and SDRM fitted to the simulated datasets. We randomly sampled 1,000 different sets of parameters in SDRM from the following ranges for each parameter: [-0.9 0.9] for *ρ*, [-2 2] for *C_M_*, and [-2 2] for the mean of all confidence criteria. In addition, we assumed that the distances between adjacent confidence criteria were equal and randomly sampled from the range [0.1 1]. In order to make SDRM identifiable, we also set *σ_M_* and *σ_C_* to 0 to remove the variation of recall and confidence criteria across trials. For each parameter set, we simulated data of one participant completing 500 trials of confidence rating and recall test, and then fit BIM and SDRM to each simulated dataset.

The correlation coefficients between fitted parameters in BIM and SDRM for recall tasks are shown in Table S1. Parameter *P_exp_* in BIM was negatively correlated with the standard deviation of confidence criteria in SDRM. Parameter *M_conf_* in BIM was negatively correlated with the mean confidence criteria in SDRM. Parameter *μ_m_* in BIM was negatively correlated with the recall criterion *C_M_* in SDRM. There was also a large positive correlation between the parameter *ρ* in the two models. These results are consistent with those reported in the main text.

Next, we used the meta-d’ model to simulate data of confidence ratings and performance in a recognition test, and compared the parameters in BIM and the meta-d’ model fitted to the simulated datasets. We randomly sampled 1,000 different sets of parameters in the meta-d’ model from the following ranges for each parameter: [-3 3] for Type I *d’*, [-3 3] for meta-*d’*, [-1 0] for the highest confidence criterion following an S1 response, and [0 1] for the lowest confidence criterion following an S2 response. In addition, we assumed that the distance between adjacent confidence criteria within S1 or S2 response was equal, which was randomly sampled from the range [0.04 0.6]. We also set the Type I criterion *C* to 0. For each parameter set, we simulated the data of 250 trials for S1 stimuli and 250 trials for S2 stimuli, and then fit BIM and the meta-d’ model to each simulated dataset. When fitting the meta-d’ model to simulated datasets, we applied padding correction to ensure all of the parameters in the meta-d’ model could be successfully estimated. In addition, we did not analyze the parameter *ρ* in BIM for recognition tasks because it is difficult to estimate the true value of *ρ*.

The correlation coefficients between fitted parameters in BIM and the meta-d’ model for recognition tasks are shown in Table S2. Parameter *P_exp_* in BIM showed a large correlation with the standard deviation of confidence criteria in the meta-d’ model. The mean confidence criteria in the meta-d’ model for S1 responses was largely correlated with the two *M_conf_* parameters in BIM for S1 responses (*M_conf (sS1, rS1)_* and *M_conf (sS2, rS1)_*). Similarly, the mean confidence criteria in the meta-d’ model for S2 responses was largely correlated with the two *M_conf_* parameters in BIM for S2 responses (*M_conf (sS1, rS2)_* and *M_conf (sS2, rS2)_*). In addition, neither *P_exp_* nor any of the *M_conf_* showed large correlations with meta-*d’*. These results are similar to those reported in the main text.

**S6. Computing the JOLs in Study 3**

In this section, we demonstrate how to directly compute the JOL for each trial in Study 3 by applying Bayesian inference without model fitting. According to the assumptions of BIM, people integrate their prior beliefs and processing experience via Bayesian inference to evaluate their memory performance. To compute the value of a JOL in each trial via Bayesian inference, we need to know (a) the processing experience in each trial, (b) participants’ prior beliefs about memory, and (c) how much experience and beliefs contribute to the metamemory process.

We assumed that the processing experience utilized in the two JOLs for young and old adults in each trial was the same, because the two JOLs were made for the same word. Thus, we could estimate the processing experience in each trial by fitting BIM to the JOL data in one experimental condition (e.g., young adult), and then use this estimated processing experience to calculate the JOL in each trial for the other condition (e.g., old adult).

In Section S2, we demonstrated that the likelihood function for BIM with continuous confidence ratings is calculated based on integration over the distribution of *x_0_*, which is perfectly correlated with the processing experience distribution (see also Equation (50)):

| $L\left( reported conf,Mem \right)=\int_{-\infty}^{\infty} f\left( reported conf \vert x_{0} \right)\left\{ Mem\cdot P\left( correct \vert x_{0} \right) \right.$ $+\left. \left( 1-Mem \right)\left[ 1-P\left( correct \vert x_{0} \right) \right] \right\}f_{N}\left( x_{0} \right)dx_{0}$ | (77) |
| --- | --- |

Thus, after we fitted BIM to the data in the young adult condition and estimated the parameters in BIM (*P_exp_*_,_ *M_conf_*, *μ_m_* and *ρ*), we could estimate the value of *x_0_* for each trial, which should maximize the likelihood function:

| $x_{0\left( i \right)}=\underset{x_{0}}{argmax}f\left( {reported conf}_{i} \vert x_{0} \right)\left\{ {Mem}_{i}\cdot P\left( correct \vert x_{0} \right) \right.$ $+\left. \left( 1-{Mem}_{i} \right)\left[ 1-P\left( correct \vert x_{0} \right) \right] \right\}$ | (78) |
| --- | --- |

in which *i* represents each trial.

Next, we could use the equation *e* = *x_0_* + *μ_e_* to compute the processing experience *e* for each trial in the young adult condition (see Section S1). The mean of the processing experience distribution (*μ_e_*) for young adult can be computed based on the following equation for the parameter *M_conf_* (see also Equation (28)):

| $M_{conf}=\Phi\left( \frac{\mu_{e}P_{exp}+\mu_{b}P_{belief}}{\sqrt{1-P_{exp}P_{belief}}} \right)=\Phi\left[ \frac{\mu_{e}P_{exp}+\mu_{b}\left( 1-P_{exp} \right)}{\sqrt{1-P_{exp}\left( 1-P_{exp} \right)}} \right]$ | (79) |
| --- | --- |

The fitted values of the parameters *P_exp_* and *M_conf_* were known after we fitted BIM to the data in the young adult condition. Thus, we needed to know the mean of the prior belief distribution (*μ_b_*) for the young adult condition in order to compute the value of *μ_e_*_._ BIM assumes that the prior belief distribution has a standard deviation of 1, and the probability that the subjective memory strength *m̂* is higher than the recall criterion 0 in the prior belief distribution represents participants’ prior belief about the proportion of recalled trials at test. This prior belief about recall proportion could be obtained from the belief-based global prediction each participant made before the learning phase, and the *μ_b_* in the young adult condition could then be computed based on the proportion that *m̂* was higher than 0 in the prior belief distribution for young adult.

After obtaining the fitted processing experience *e* in each trial from the young adult condition, we could compute the predicted value of JOL for the old adult condition in each trial based on this processing experience (see also Equation (7)):

| $predicted conf=P\left( \hat{m}>0 \vert e \right)=\Phi\left( \frac{e+{\sigma_{l}}^{2}\mu_{b}}{\sigma_{l}\sqrt{1+{\sigma_{l}}^{2}}} \right)$ | (80) |
| --- | --- |

Before computing the JOLs in the old adult condition, we still needed to know (a) the mean of prior belief distribution (*μ_b_*) for the old adult condition, and (b) the standard deviation of the likelihood function in the Bayesian inference (*σ_l_*) for the old adult condition, which determines how much processing experience and prior beliefs contributed to JOLs for old adults. The *μ_b_* for the old adult condition could be obtained from the belief-based global prediction each participant made before the learning phase. *σ_l_* was computed from *P_exp_*, which could be obtained through Equation (79) based on the parameters *M_conf_*, *μ_e_* and *μ_b_* in the old adult condition. Here we approximated the value of *M_conf_* in the old adult condition using the mean JOLs for old adult in all trials within each participant. In addition, *μ_e_* for the old adult condition should be equal to that for the young adult condition.

After we obtained the processing experience in each trial estimated from the young adult condition, and the parameters *μ_b_* and *σ_l_* in the old adult condition for each participant, we could directly compute the JOL for the old adult condition in each trial without fitting BIM to JOL data in this condition. Similarly, we could compute the JOL for the young adult condition in each trial based on the processing experience estimated from the old adult condition. Then we examined the correlation between the computed and true JOLs to see whether the true value of JOLs could be predicted by our Bayesian inference computation.

**References**

Angus, J. E. (2005). The Probability Integral Transform and Related Results. *SIAM Review*. https://doi.org/10.1137/1036146

Murphy, K. P. (2007). Conjugate Bayesian Analysis of the Gaussian Distribution. *Def*. https://doi.org/10.1.1.126.4603‎

Stanislaw, H., & Todorov, N. (1999). Calculation of signal detection theory measures. *Behavior Research Methods, Instruments, and Computers*. https://doi.org/10.3758/BF03207704

Table S1

*Spearman correlation between parameters in BIM and SDRM for recall tasks*

|  | P_exp_ | M_conf_ | μ_m_ | ρ |
| --- | --- | --- | --- | --- |
| **SDRM** |  |  |  |  |
| ρ | -0.006 | 0.007 | -0.007 | 0.974^a^ |
| M_Cconf_ | -0.010 | -0.879^a^ | 0.040 | -0.017 |
| SD_Cconf_ | -0.757^a^ | -0.002 | -0.007 | 0.022 |
| C_M_ | 0.026 | 0.061 | -0.994^a^ | -0.001 |

^a^ The absolute value of the correlation coefficient is higher than 0.5.

Table S2

*Spearman correlation between parameters in BIM and the meta-d’ model for recognition tasks*

|  | P_exp_ | M_conf (sS1, rS1)_ | M_conf (sS1, rS2)_ | M_conf (sS2, rS1)_ | M_conf (sS2, rS2)_ |
| --- | --- | --- | --- | --- | --- |
| **Meta-d’** |  |  |  |  |  |
| meta-d’ | -0.031 | 0.423 | -0.450 | -0.476 | 0.427 |
| M_CrS1_ | 0.367 | 0.523^a^ | 0.002 | 0.521^a^ | -0.019 |
| SD_CrS1_ | -0.596^a^ | -0.408 | -0.079 | -0.386 | -0.072 |
| M_CrS2_ | -0.356 | 0.044 | -0.505^a^ | 0.006 | -0.502^a^ |
| SD_CrS2_ | -0.565^a^ | -0.006 | -0.371 | -0.072 | -0.343 |

^a^ The absolute value of the correlation coefficient is higher than 0.5.

Table S3

*The fitted value of M_conf_ in Study 4*

|  | M_conf (sS1, rS1)_ | M_conf (sS1, rS2)_ | M_conf (sS2, rS1)_ | M_conf (sS2, rS2)_ |
| --- | --- | --- | --- | --- |
| **Experimental group** |  |  |  |  |
| Words, pre-training session | 0.66 (0.11) | 0.43 (0.26) | 0.43 (0.24) | 0.69 (0.12) |
| Words, post-training session | 0.78 (0.10) | 0.55 (0.23) | 0.58 (0.22) | 0.76 (0.12) |
| Shapes, pre-training session | 0.64 (0.15) | 0.52 (0.24) | 0.41 (0.25) | 0.65 (0.14) |
| Shapes, post-training session | 0.71 (0.13) | 0.43 (0.22) | 0.43 (0.20) | 0.71 (0.13) |
| **Control group** |  |  |  |  |
| Words, pre-training session | 0.67 (0.12) | 0.41 (0.28) | 0.38 (0.26) | 0.68 (0.13) |
| Words, post-training session | 0.65 (0.19) | 0.48 (0.29) | 0.44 (0.26) | 0.68 (0.17) |
| Shapes, pre-training session | 0.58 (0.15) | 0.38 (0.25) | 0.36 (0.23) | 0.60 (0.12) |
| Shapes, post-training session | 0.56 (0.16) | 0.38 (0.24) | 0.37 (0.24) | 0.57 (0.18) |

*Notes*. Standard deviations are reported in parentheses.


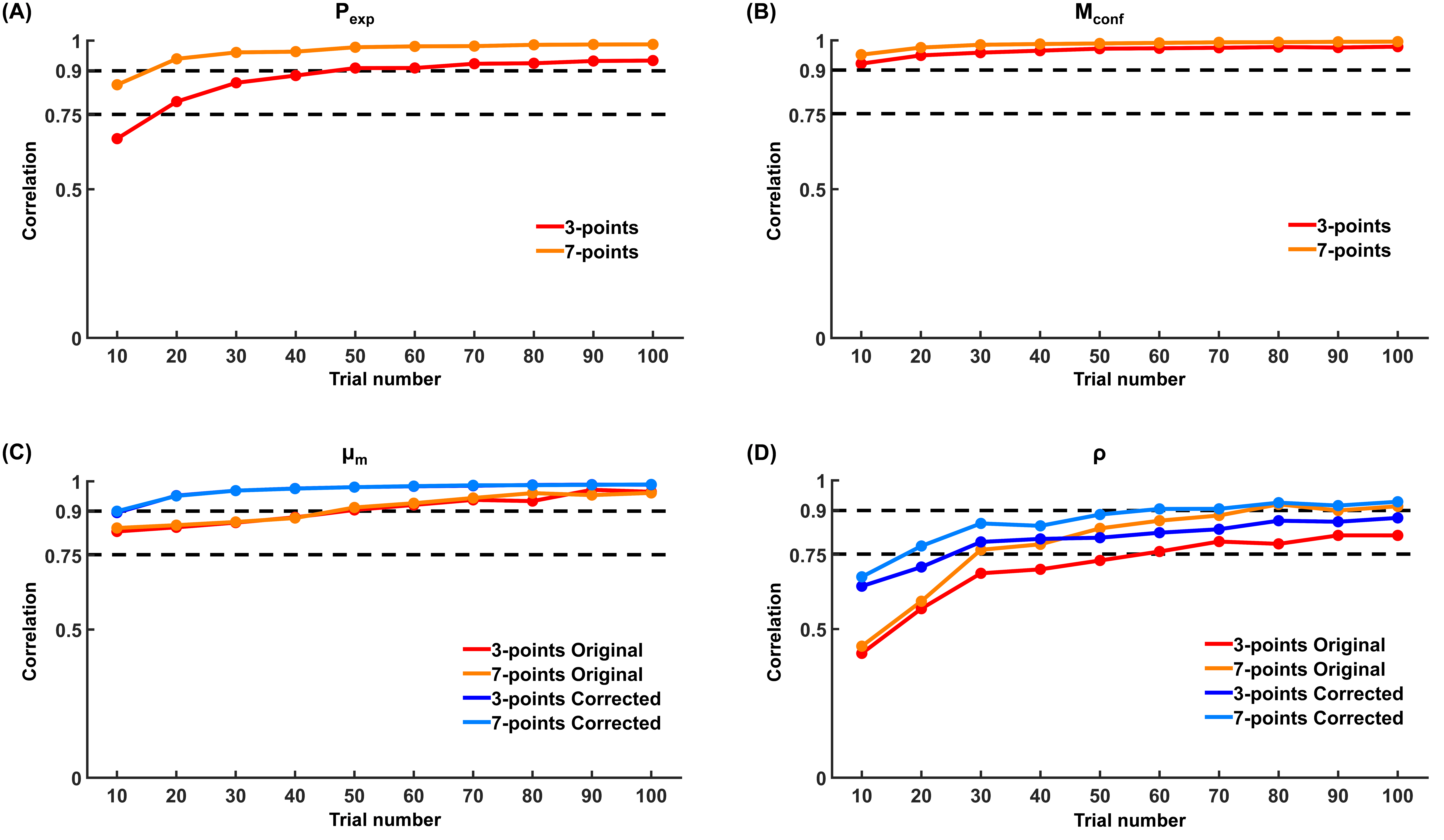
*Figure S1*. Results of parameter recovery for *P_exp_* (A), *M_conf_* (B), *μ_m_* (C) and *ρ* (D) in BIM for recall tasks with discrete confidence (on a 3-point or 7-point scale) as a function of the number of trials. The two dashed lines represent that the correlation between true and fitted parameter values is 0.75 and 0.9, respectively. The original correlation (for all simulated datasets) and corrected correlation (after we removed the datasets with the same performance in all trials) are shown for the parameters *μ_m_* and *ρ*. The correlation for *μ_m_* is very similar for the 3-point and 7-point scales, and the lines for 3-point and 7-point scales are largely overlapped in (C).

*Figure S2*. Results of parameter recovery for *P_exp_* (A), *M_conf_* in the 2 (Stimulus: S1 vs. S2) × 2 (Response: S1 vs. S2) conditions (B-E), and *ρ* (F) in BIM for recognition tasks with discrete confidence (on a 3-point or 7-point scale), as a function of the number of trials. The two dashed lines represent that the correlation between true and fitted parameter values is 0.75 and 0.9, respectively.
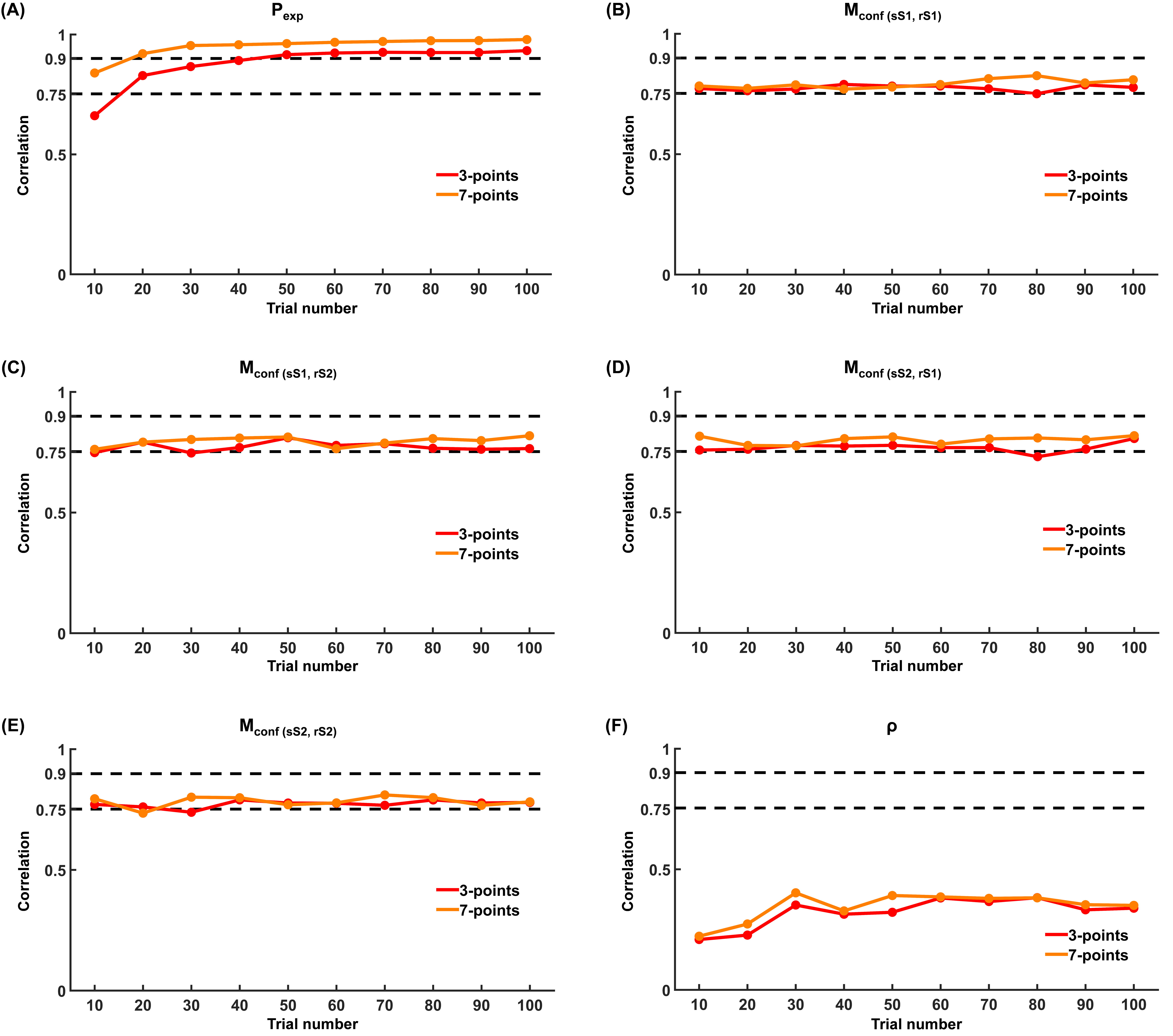

Supplement: Supplementary file 1 [file REV-2019-1431_Suppl.docx]
